# Heritable transcriptional defects from aberrations of nuclear architecture

**DOI:** 10.1038/s41586-023-06157-7

**Published:** 2023-06-07

**Authors:** Stamatis Papathanasiou, Nikos A. Mynhier, Shiwei Liu, Gregory Brunette, Ema Stokasimov, Etai Jacob, Lanting Li, Caroline Comenho, Bas van Steensel, Jason D. Buenrostro, Cheng-Zhong Zhang, David Pellman

**Affiliations:** 1 Department of Cell Biology, Blavatnik Institute, Harvard Medical School, Boston, MA USA; 2 Department of Pediatric Oncology, Dana-Farber Cancer Institute, Boston, MA USA; 3 Single-Cell Sequencing Program, Dana-Farber Cancer Institute, Boston, MA USA; 4 Department of Data Sciences, Dana-Farber Cancer Institute, Boston, MA USA; 5 Department of Biomedical Informatics, Blavatnik Institute, Harvard Medical School, Boston, MA USA; 6 Broad Institute of MIT and Harvard, Cambridge, MA USA; 7 Department of Stem Cell and Regenerative Biology, Harvard University, Cambridge, MA USA; 8 Gene Regulation Observatory, Broad Institute of MIT and Harvard, Cambridge, MA USA; 9 Division of Gene Regulation and Oncode Institute, The Netherlands Cancer Institute, Amsterdam, The Netherlands; 10 Howard Hughes Medical Institute, Chevy Chase, MD USA; 11 Present Address: Institute of Molecular Biology, Mainz, Germany; 12 Present Address: Department of Chemistry and Chemical Biology, Harvard University, Cambridge, MA USA; 13 Present Address: AstraZeneca, Waltham, MA USA

**Keywords:** Cancer genomics, Tumour heterogeneity, Chromosome segregation, Cellular imaging, Chromosomes

## Abstract

Transcriptional heterogeneity due to plasticity of the epigenetic state of chromatin contributes to tumour evolution, metastasis and drug resistance^1–3^. However, the mechanisms that cause this epigenetic variation are incompletely understood. Here we identify micronuclei and chromosome bridges, aberrations in the nucleus common in cancer^4,5^, as sources of heritable transcriptional suppression. Using a combination of approaches, including long-term live-cell imaging and same-cell single-cell RNA sequencing (Look-Seq2), we identified reductions in gene expression in chromosomes from micronuclei. With heterogeneous penetrance, these changes in gene expression can be heritable even after the chromosome from the micronucleus has been re-incorporated into a normal daughter cell nucleus. Concomitantly, micronuclear chromosomes acquire aberrant epigenetic chromatin marks. These defects may persist as variably reduced chromatin accessibility and reduced gene expression after clonal expansion from single cells. Persistent transcriptional repression is strongly associated with, and may be explained by, markedly long-lived DNA damage. Epigenetic alterations in transcription may therefore be inherently coupled to chromosomal instability and aberrations in nuclear architecture.

## Abstract (web summary)

Micronuclei, which are common features of nuclei in cancer cells, can generate heritable sources of transcriptional suppression, a finding that establishes an inherent relationship between chromosomal instability and variation in chromatin state and gene expression.

## Main

Nuclear atypia, which encompasses aberrations in nuclear size and morphology, is a hallmark feature of many tumours that is commonly used to assign tumour grade and predict patient prognosis^4,6,7^. Recently, our group and others demonstrated that structural abnormalities of the nucleus—micronuclei or chromosome bridges—can lead to various simple and complex chromosomal rearrangements, including chromothripsis^8–11^. This process is an extensive form of chromosome fragmentation and rearrangement that is common in cancer^12–14^. Although the role of nuclear abnormalities in the generation of genetic instability is now appreciated, other consequences of nuclear atypia have been little studied. For example, although micronuclei can have transcription defects and altered chromatin marks^15–17^, the functional consequences of these alterations remain unclear.

## Transcriptome analysis by Look-Seq2

Micronuclei form from mis-segregation of intact chromosomes or acentric chromosome fragments. In the first cell cycle after the formation of the micronucleus (hereafter termed generation 1), >50% of micronuclei undergo nuclear envelope (NE) rupture and acquire DNA damage^15,18,19^, which is partly explained by a pathological form of DNA base excision repair^20^. There is a second wave of DNA damage that can occur on any of these chromosomes when the cell enters mitosis, even if the NE of the micronucleus remains intact until mitotic entry^11^. After cell division, the micronuclear chromosome (MN chromosome) can remain in the cytoplasm and reform a micronucleus, be re-integrated en bloc into one daughter cell nucleus or have fragments re-incorporated into both daughter nuclei^8,18^. End joining of chromosome fragments in daughter nuclei generates chromothripsis^12,21^.

**Extended Data Fig. 1:**
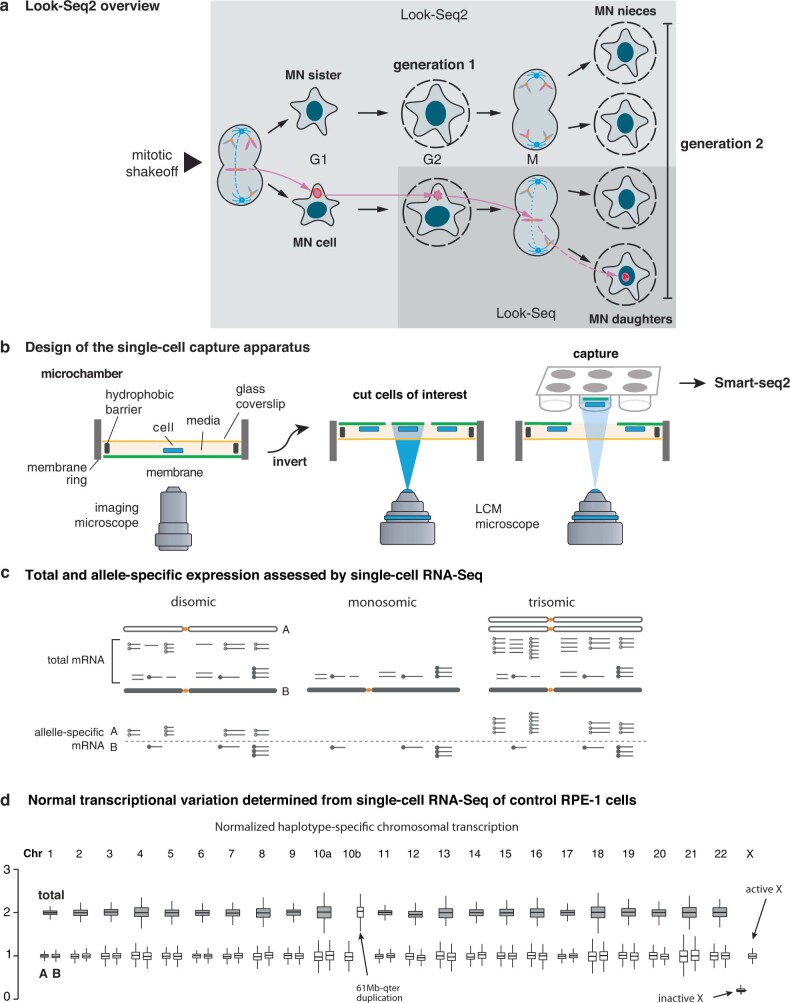
Overview of experimental and analytical workflows. (a) Scheme of Look-Seq2. There are two key improvements compared to the original Look-Seq. First, live-cell imaging starts before the first cell division that leads to micronuclei; this enables tracking, isolation, and transcriptome analysis of both the MN cell and its sister cell (“generation 1”). Moreover, we can image cells over two cell divisions (“generation 2”) and analyze both the daughters of the MN cell (“MN daughters”) and the daughters of the MN sister cell (“MN nieces”). The second improvement is that single cells are isolated using a new capture strategy with minimal mechanical perturbation that is illustrated in (b). (b) Second generation experimental strategy for single-cell capture and sequencing. We adapted a previously developed LCM system (Palm Microbeam, Carl Zeiss) and re-designed the imaging and capture setup. The modifications enable the inversion of the membrane rings relative to the microscope objective. This allows medium to be present continuously throughout capture, which provides more time for the capture of family member cells. The setup is also compatible with laser catapulting into 96 well plates, which further increases throughput. See Methods for details. (c) Two measures of transcription yield from single-cell RNA-Seq data: (1) The total transcriptional yield is assessed by the transcripts per million (TPM) calculated from all RNA-Seq fragments overlapping with annotated coding regions. (2) The fraction of transcripts derived from each parental homologue is estimated from the counts of haplotype-specific sequencing reads. The haplotype-specific transcription yield is estimated by multiplying the total transcriptional yield by the haplotype fraction of transcripts. The transcription level of each gene in a single cell is further normalized by its mean in normal RPE-1 cells to obtain the normalized transcription of each gene. Details of the computational analysis are provided in Methods. (d) Normal range of transcriptional variation of each parental homologue derived from single-cell RNA-Seq data of control RPE-1 cells (*n* = 198; for Chr.12 *n* = 190 after excluding trisomies). Shown are the range of mean transcription of each chromosome (mean TPM ratio across all genes on a chromosome in each cell; shaded boxes) and the range of haplotype-specific transcription (mean haplotype-specific TPM ratio across all genes on a chromosome in each cell, open boxes) calculated from the total transcription and the haplotype fractions. Box plots indicate the 1st (bottom edge) and 3rd (top edge) quartiles and the median (horizontal line), with whiskers indicating 1.5x the interquartile range. The range of total transcriptional variation is used to estimate the range of normal *disomic* transcription (i.e., transcription of two copies of a chromosome, either from one copy of both parental homologues or from two copies of one homologue); the range of haplotype-specific transcriptional variation is used to estimate the range of normal transcription from each parental homologue. For the trisomic Chr.10q segment (61Mb-qter), the two haplotype-specific TPM ratios reflect the transcriptional output of the single-copy homologue (A) and the duplicated homologue (B); for Chr.X, the haplotype-specific TPM ratios reflect the transcriptional output of the active X (Xa) and the inactive X (Xi). For the 10q segment and Chr.X, the haplotype-specific TPM ratios are calculated by normalizing the TPM ratio of the intact 10q (A homologue) and Xa to 1. The duplicated 10q segment is appended to the q-terminus of the active X.

A direct assessment of the transcriptional consequences of micronucleation requires single-cell transcriptome analysis, which we performed with a modified method for live-imaging and single-cell whole-genome sequencing^8,11^ (Methods). We induced chromosome mis-segregation and generated micronucleated RPE-1 cells using a nocodazole-induced mitotic block and release procedure^8^. We assessed the loss of micronuclear NE integrity by live-cell imaging (Supplementary Videos 1 and 2). Micronucleated cells or their daughter cells were then isolated for transcriptome analysis^22^ (Extended Data Fig. 1a). Initially, we isolated cells using the approach we used for single-cell whole-genome sequencing^8,11^. However, for most of the experiments in this study, we developed an improved contact-free laser capture microdissection^23^ method (Extended Data Fig. 1b and Methods). The updated capture method is optimized for isolating cells with minimal perturbation and, because of an in-house fabricated culture chamber, it is also optimized for the isolation of daughter cells, sister cells or niece cells of the micronucleated cell. We refer to this method, using either type of cell capture technique, as Look-Seq2.

**Extended Data Fig. 2:**
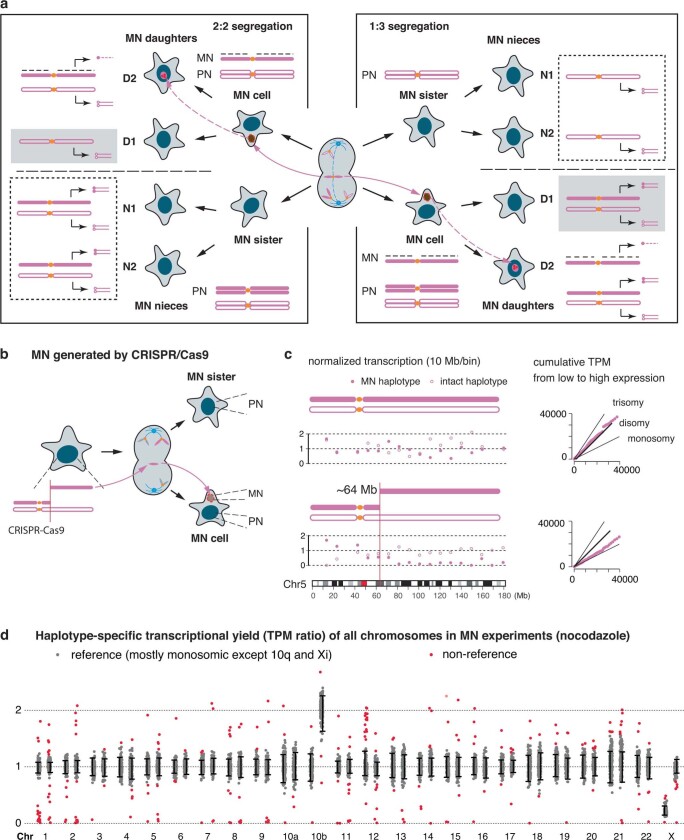
General strategy for the inference of haplotype-specific DNA copy number and chromosome mis-segregation events from single-cell RNA-Seq data. (a) Two segregation patterns of MN chromosomes (left: 2:2 segregation; right 1:3 segregation) generated by nocodazole-block-and-release and the predicted copy-number outcomes over two generations. MN chromatids (filled magenta) and chromatids of the other haplotype (open magenta) are represented in the same fashion as in Figs. 1 and 2. Under 2:2 segregation, the MN sister cell or the MN nieces (dashed boxes) should display bi-allelic disomic transcription but one of the two MN daughter cells (shaded boxes) should display mono-allelic transcription of the intact haplotype (open magenta) due to deficient replication of the MN chromatid; under 1:3 segregation, the MN sister cell or the MN nieces should display mono-allelic transcription from the intact haplotype (open magenta). The predicted monosomic transcription outcomes are used to identify micronuclear chromosomes and their segregation pattern in each experimental family. (b) and (c) Validation of the transcriptional outcomes of MN (“generation 1”) using an experimental strategy (b) of inducing MN with acentric Chr.5q fragments generated by CRISPR-Cas9 as reported in our recent study^25^. The data shown in (c) demonstrate the predicted transcriptional outcome when the micronucleus contains only one copy of Chr.5q fragment arm that most closely resembles the segregation patterns generated by nocodazole block-and-release. Two measures of gene transcription are shown: in the left plot, filled and open magenta circles are the normalized allelic expression of the broken and the intact haplotype in 10 Mb bins; on the right are the cumulative TPM (from low to high expression). The MN sister cell shows normal disomic transcription. In the MN cell, monoallelic transcription of the MN haplotype (filled circles) extending from near 64 Mb to the q-terminus indicates silencing of an acentric Chr.5 fragment partitioned into the micronucleus after Cas9-breaks generated at ~64 Mb. Reduced transcription of Chr.5 in the MN cell is also evident from the cumulative TPM plot on the right that shows a reduction in total transcription relative to normal disomic Chr.5. As the cumulative TPM plot is generated for all genes on Chr.5, it does not distinguish chromosome-wide transcriptional reduction from regional loss of transcription. (d) Identification of chromosomes with non-reference transcriptional states (red dots) in MN families (*n* = 173 cells) based on reference transcription distributions determined from control RPE-1 cells (Extended Data Fig. 1d). Based on the inferred DNA copy-number states of these chromosomes (assuming proportional transcriptional yield and DNA copy number), we further identify chromosomes with mis-segregation patterns consistent with the predicted outcomes in (a). Error bars represent normal range of transcription estimated based on the 5 % and 95 % values in control cells. Red dots represent chromosomes with significant deviations (Bonferroni corrected *P* <0.05, two-tailed *Z*-test; for 48 chromosomes including both homologues).

To assess micronucleation-induced transcriptional changes, we needed to identify the chromosome that was in the micronucleus, determine the copy number of this chromosome and then compare the transcriptional output of this chromosome to the expectation based on the DNA copy number (Extended Data Fig. 1c). These goals were accomplished using haplotype-resolved transcriptome analysis of Look-Seq2 data of the cell of interest combined with transcriptome analysis of its family members (Methods, Supplementary Table 1 and Extended Data Figs. 1 and 2). Haplotype-resolved transcriptome analysis correctly identified clonal 10q trisomy (based on a 2:1 allelic imbalance) and the low transcription output from the inactive X chromosome in female RPE-1 cells (Extended Data Fig. 1d).

**Fig. 1:**
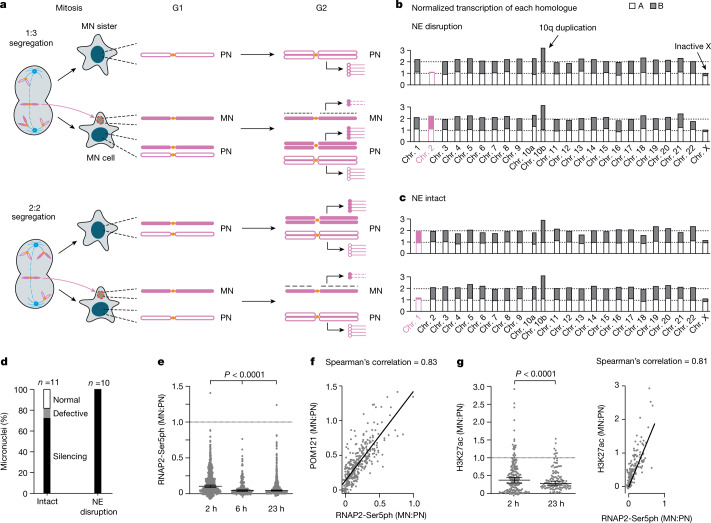
Transcription defects in newly generated micronuclei. **a**, Two patterns of chromosome segregation that generate a micronucleated cell (MN cell) and its sister (MN sister). Filled magenta shapes indicate the mis-segregated chromatid in the micronucleus (MN) and its sister chromatids in the primary nucleus (PN); open magenta shapes indicate sister chromatids of the other homologue. Top, a 1:3 mis-segregation generates monosomy in a MN sister cell and trisomy in a MN cell. Bottom, a 2:2 segregation generates disomy in both cells. In G2 (when cells were isolated), chromosomes in the primary nucleus are replicated but the MN chromatid is poorly replicated. Lollipops represent transcripts (open circles for transcripts from the normal homologue; filled circles for transcripts from the MN homologue; dashed lines for transcripts from the MN chromatid). **b**, Normalized transcription yield of each chromosome in a MN cell and sister cell pair after 1:3 mis-segregation. Filled and open bars indicate transcription from different homologues assessed by the parental haplotypes; filled magenta bars for the MN homologue (Chr.2B); open magenta bars for the normally segregated homologue (Chr.2A). Monosomic transcription of Chr.2B in the MN cell (bottom) is from the normally segregated chromatid in the primary nucleus and indicates near-complete silencing of the MN chromatid. **c**, Chromosome-wide silencing of an intact micronucleus generated by a 2:2 segregation, similar to **b**. The MN homologue is Chr.1B. **d**, Summary of transcription output in 21 MN cell–MN sister pairs, grouped by the status of MN nuclear envelope (NE) integrity. See also Supplementary Table 2 and Extended Data Fig. 3 for more information related to **b**–**d** **e**, RNAP2-Ser5ph signal (MN:PN ratios of background normalized fluorescent intensities) at the indicated time points after MN formation (left to right, *n* = 644, 212 and 605 from 2 or 3 experiments). Boxes indicate median ratio with a 95% confidence interval (CI), *P* values from two-tailed Mann–Whitney test. **f**, Correlation between micronuclei transcription (RNAP2-Ser5ph intensity) and nuclear pore complex density (POM121, 2 h after mitotic shake-off), (*n* = 334 from 3 experiments). Two-tailed Spearman’s correlation. **g**, Left, MN:PN ratios for H3K27ac (left to right, *n* = 187 and 118 from 2 experiments), analysed as in **e**. Right, correlation between H3K27ac and RNAP2-Ser5ph signals (2 h after shake-off; *n* = 187 from 2 experiments). Two-tailed Spearman’s correlation. Source Data

We next needed to identify the MN chromosome and determine its copy number, which sets the expectation for the normal transcription output of that chromosome. The identity of the MN chromosome was inferred from the pattern of mis-segregation, which we determined from the transcriptomes of the family members of the micronucleated cell. Because the family member cells have normal nuclei, their  transcription output is proportional to DNA copy number^24^. Once the chromosome content of the family members is known, the pattern of mis-segregation that generates the micronucleated cell can be deduced, which then enables the determination of the copy number of the chromosome in the micronucleus (Extended Data Fig. 2a and Methods). As an example, monosomic transcription in the sister of a micronucleated cell indicated that the micronucleated cell has to be trisomic for that chromosome (a 1:3 segregation; Fig. 1a). This solves the problem of assigning DNA copy number without making assumptions about whether the chromosome from the micronucleus is normally transcribed or not (Methods).

## Transcription defects in micronuclei

**Extended Data Fig. 3:**
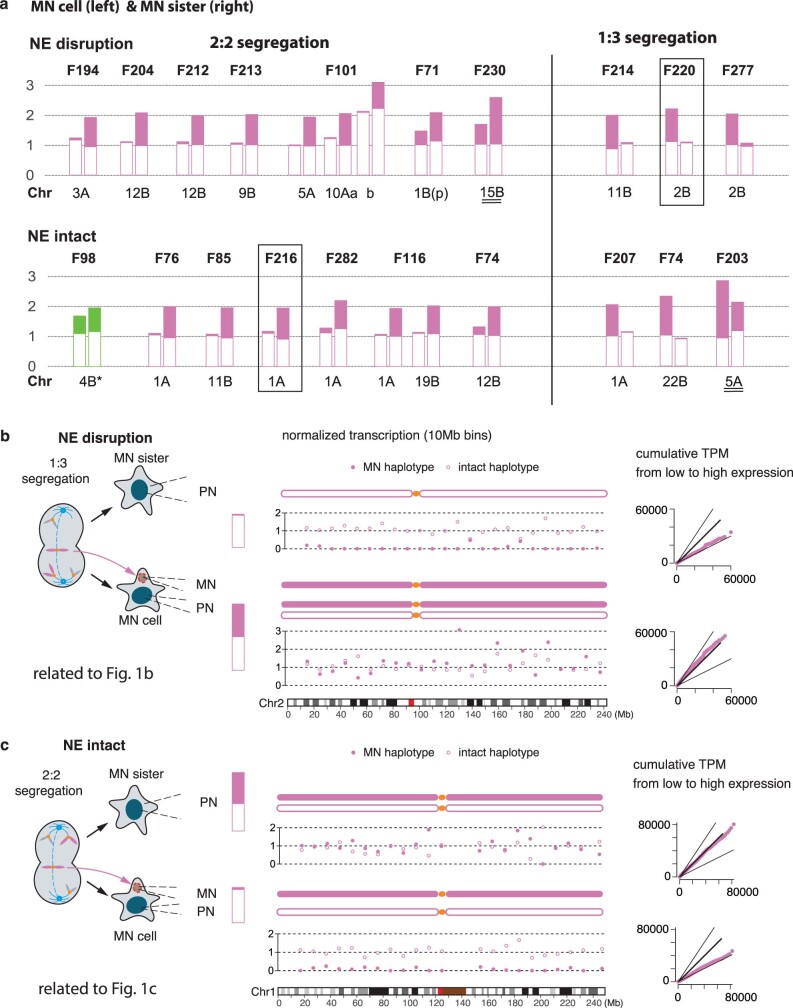
Additional data on the loss of transcription in newly generated (generation 1) MN. (a) Summary of the transcriptional yield of MN chromosomes in all generation 1 families. Each bar plot represents the average transcriptional yield of both homologues of the MN chromosome (filled for the micronuclear homologue that is annotated below each plot; open for the intact homologue) in the MN cell (left) and the MN sister (right) in each family. Two families with near identical transcription from all chromosomes (indicating normal transcription yield of the MN chromosome) are not shown. In family F98, the MN haplotype (Chr.4B, green) displays reduced transcription in the MN cell; in all the other families, the MN haplotype in the MN cell displayed near complete silencing as indicated by either near complete loss of transcription (2:2 segregation, left) or close to monosomic transcription (1:3 segregation, right) from the intact sister chromatid of the MN haplotype in the primary nucleus. In family F71, the transcriptional imbalance is restricted to the 1p arm with near complete silencing (see Supplementary Table 2). In family F230 and F203, the transcriptional pattern indicates an extra copy of the MN homologue that is shared between the MN cell and the MN sister reflecting a pre-existing duplication of the MN homologue (i.e., a pre-existing trisomy). The examples shown in panels **b** (F220) and **c** (F216) are highlighted. (b) Chromosome-wide transcriptional data of both Chr.2 haplotypes (left) in the MN family shown in Fig. 1b (F220) and plots of cumulative TPM from low to highly expressed genes (right) plots that validate the inference of monosomic and disomic Chr.2 transcription. (c) Chromosome-wide transcriptional data of both Chr.1 haplotypes in the MN family shown in Fig. 1c (F216) and cumulative TPM plots that validate the inference of monosomic and disomic Chr.1 transcription.

As an initial validation of Look-Seq2, we analysed micronuclei containing acentric 5q chromosomal arms generated by CRISPR–Cas9 cleavage^25^. We focused on micronuclei that had undergone NE rupture because NE rupture causes abrupt transcriptional silencing^15^. We identified the transcriptional defects expected from partitioning of Cas9-generated acentric fragments into micronuclei^25^ (Extended Data Fig. 2b). As further validation, we generated micronuclei by random whole chromosome mis-segregation and confirmed near-complete transcriptional silencing of chromosomes from micronuclei after NE rupture (Fig. 1b, Extended Data Fig. 3a,b and Supplementary Table 2).

**Extended Data Fig. 4:**
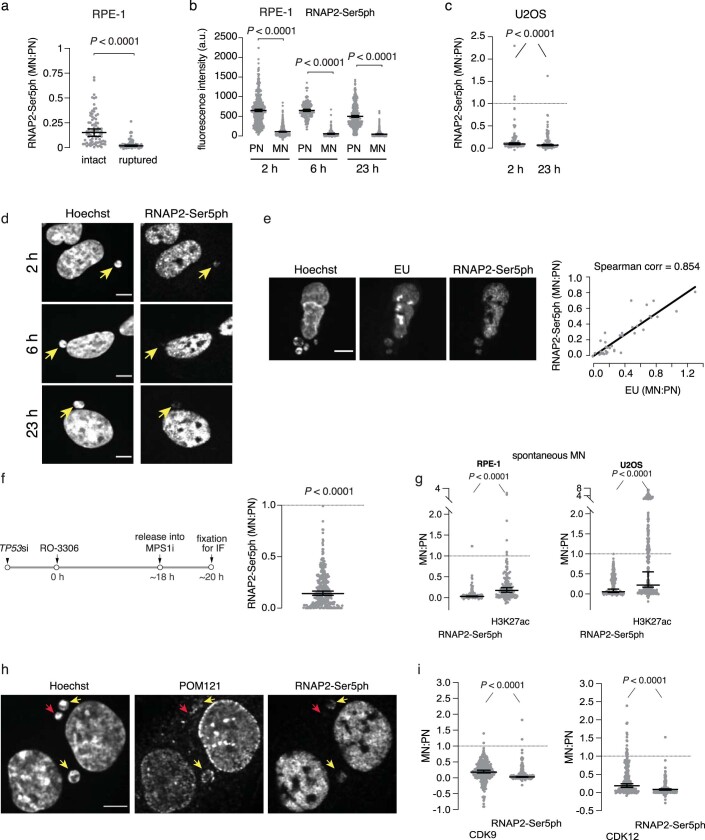
Transcription defects and chromatin modifications in newly formed (generation 1) micronuclei. (a) Transcription is present at a reduced level in intact MN and is nearly absent in ruptured MN. Data points are background normalized MN:PN fluorescence intensity (FI) ratios of RNAP2-Ser5ph at 23 h post mitotic shake-off. RFP-NLS levels were used to assign the micronuclei in the two groups (*n* = 83 and 82, left to right, from three experiments). Micronuclei with NLS ratios below 0.1 relative to the PN were considered ruptured and above 0.3 were considered intact. Median with 95% confidence interval (CI); Two-tailed Mann–Whitney test. (b) Data from Fig. 1e, but instead of the MN:PN FI ratios what is shown here are the background normalized intensity values at 2, 6 and 23 h post release from nocodazole and mitotic shake-off (*n* = 644 for 2 h, 212 for 6 h and 605 for 23 h, from two or three experiments). Median with 95% CI; Kruskal-Wallis with Dunn’s multiple comparisons test. (c) In U2OS cells, active transcription (RNAP2-Ser5ph) is also reduced in newly formed MN. Performed and analyzed as in Fig. 1e (*n* = 88 and 104, left to right, from two experiments). (d) Representative images from the data shown in Fig. 1e. Yellow arrows indicate micronuclei. Scale bars 5 µm. (e) Independent confirmation of the MN transcription defect by 30 min EU pulse labeling. Left, representative images of S/G2 cells with MN (generation 1). Right, correlation between RNAP2-Ser5ph and EU levels. Cells with varying levels of RNAP2-Ser5ph intensity were selected and then EU intensity levels were measured (*n* = 37, from one experiment). Note the strong EU signal in the nucleoli which lack RNAP2-Ser5ph, because rDNA is transcribed primarily by RNA polymerase I and RNA polymerase III. Two-tailed Spearman’s correlation. Scale bar 5 µm. (f) MN transcription defects verified in MN generated by G2 arrest with CDK1 inhibition, followed by release into an MPS1 inhibitor. This synchronization and MN induction method differs from the nocodazole block and release protocol primarily used in this study because it shortens rather than lengthens mitosis (excluding hypothetical artifacts from prolonged mitotic arrest). Left: scheme of the experiment. RPE-1 cells were analyzed 2 h after release from the G2 block (*n* = 334, from three experiments). Right: quantification and analysis of the results as in Fig. 1e. (g) Transcription and chromatin defects in spontaneously generated micronuclei. Decreased levels of RNAP2-Ser5ph and H3K27ac in spontaneous micronuclei of untreated RPE-1 (left) and U2OS cells (right). Performed and analyzed as in Fig. 1e (*n* = 134 and 295, left to right, from two experiments). (h) Representative images from data shown in Fig. 1f. Yellow arrows indicate micronuclei with nucleoporin signal (POM121) and transcription (RNAP2-Ser5ph). In contrast, red arrows indicate a micronucleus with decreased nucleoporin signal and much lower transcription signal. Note that we evaluated the specificity of POM121 staining by confocal microscopy, showing the typical nuclear pore complex dot-like pattern at the nuclear surface and the increased rim signal at the nuclear periphery by imaging a focal plane in the middle of the cell. Scale bar 5 µm. (i) Reduced accumulation of CDK9 and CDK12 in the micronuclei. The levels of CDK9, CDK12 and RNAP2-Ser5ph were analyzed in micronuclei 23 h post release from a nocodazole block followed by a mitotic shake-off. The experiment was performed and analyzed as in Fig. 1e (*n* = 285 and 291, left to right, from two experiments). An analysis of intact micronuclei also showed the defective accumulation of both CDK9 and CDK12 (MN:PN ratios: 0.35 for CDK9 and 0.09 for RNAP2-Ser5ph;  *n* = 111, *P* < 0.0001; 0.28 and 0.08 MN:PN for CDK12 and RNAP2-Ser5ph groups, respectively, *n* = 102, *P* < 0.0001; from two experiments). Source Data

We further used Look-Seq2 to assess transcription before NE rupture (generation 1) and found that most intact micronuclei exhibited significant transcriptional suppression (Fig. 1c and Extended Data Fig. 3c). Among 11 intact micronuclei, 2 were inferred to have normal transcription, 1 showed a partial defect and the rest showed significantly reduced transcription or near-complete transcriptional silencing (Fig. 1d, Extended Data Fig. 3a and Supplementary Table 2). Defective transcription from both intact and ruptured micronuclei was confirmed by fluorescence intensity (FI) measurements for a marker of active, phosphorylated RNA polymerase II (RNAP2-Ser5ph; Extended Data Fig. 4). The transcription defect of intact micronuclei was evident from the beginning of interphase (Fig. 1e and Extended Data Fig. 4b–d), and the degree of RNAP2-Ser5ph loss was positively correlated to the extent of the defect in nuclear pore complex assembly (Fig. 1f and Extended Data Fig. 4h). Our previous studies demonstrated that the defect in assembly of the nuclear pore complex is itself correlated with micronuclear defects in nuclear import^26,27^. Consistent with the idea that micronuclei lack the normal complement of transcription machinery proteins, CDK9 and CDK12, which are both required for transcription elongation, exhibited reduced recruitment to micronuclei (Extended Data Fig. 4i). Together, these data demonstrate that almost all newly generated micronuclei—ruptured or intact—exhibit defective transcription.

**Extended Data Fig. 5:**
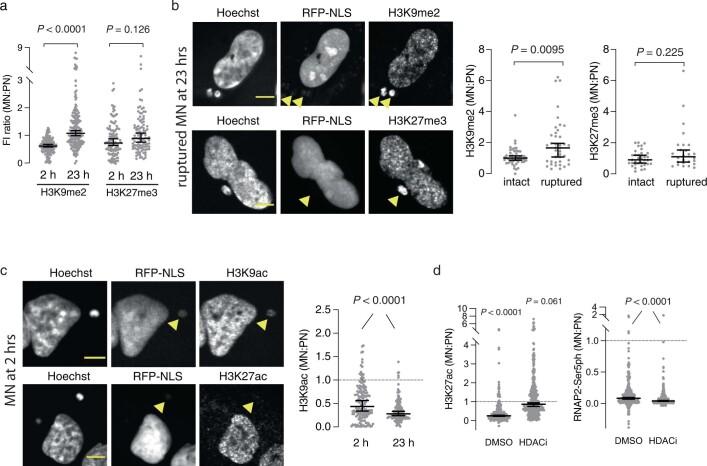
Epigenetic alterations in micronuclei. (a) Modest increase of repressive chromatin marks in a subset of late S/G2 MN. Performed and analyzed as in Extended Data Fig. 4a (*n* = 129, 179, 114 and 105, left to right, from two experiments for H3K9me2; from one or two experiments for H3K27me3). (b) Left, selected example images from (a) of cells with ruptured MN that show apparent enrichment for H3K9me2 and HK27me3 in the MN. Arrowheads: micronuclei lacking normal RFP-NLS accumulation. Right: related to (a) but comparing intact and ruptured MN for H3K9me2 (*n* = 48 and 40, left to right, from two experiments) and H3K27me3 (*n* = 35 and 26, left to right, from two experiments) at 23 h post mitotic shake-off. Performed and analyzed as in Extended Data Fig. 4a. Scale bars 5 µm. (c) Loss of H3K9ac and H3K27ac in MN at the indicated timepoint during interphase. Left: representative images of the indicated histone modifications at 2 h post mitotic shake-off. Right: quantification and analysis of data for H3K9ac as in Fig. 1e (*n* = 148 and 124, left to right, from two experiments). Scale bars 5 µm. (d) HDAC inhibition is not sufficient to rescue the transcription defect of chromosomes in micronuclei. Cells were analyzed after incubation with a pan-HDAC inhibitor (SAHA) for 23 h post release from a nocodazole block followed by a mitotic shake-off (*n* = 302 and 335, left to right, from three experiments for both H3K27ac and RNAP2-Ser5ph). For the intact micronuclei, a significant rescue of H3K27ac levels was also observed after HDACi treatment (0.36 and 0.87 MN:PN for H3K27ac in DMSO and HDACi groups, respectively, *P* < 0.0001) and this was also not detectably accompanied by rescue of the transcription defect (0.14 and 0.07 MN:PN for RNAP2-Ser5ph in DMSO and HDACi groups, respectively) (*n* = 165 and 131 for both H3K27ac and RNAP2-Ser5ph). Performed and analyzed as in Fig. 1e. All pairwise comparisons between DMSO and HDACi have *P* < 0.0001. For the intact MN, all pairwise comparisons between MN and PN have *P* < 0.0001, except for the HDACi of H3K27ac group that has *P* = 0.502. Source Data

The transcriptional defects in MN chromosomes correlated with alterations in epigenetic chromatin marks. There was a modest increase in the repressive marks histone 3 lysine 9 dimethylation (H3K9me2) and histone 3 lysine 27 trimethylation (H3K27me3) that accumulated on a subset of micronuclei with NE disruption late during interphase (Extended Data Fig. 5a,b), a result consistent with a previous report^16^. Moreover, micronuclei exhibited loss of the active chromatin marks histone 3 lysine 27 acetylation (H3K27ac) and histone 3 lysine 9 acetylation (H3K9ac)^15,16^ from the beginning of interphase, which correlated with reductions in the level of active RNAP2 (Fig. 1g and Extended Data Fig. 5c). This highly penetrant loss of H3K27ac is notable because recent studies have indicated that recovery of H3K27ac is essential for the normal reestablishment of transcription after mitosis^28–30^. Multiple factors probably contribute to the transcription defects of micronuclei because inhibition of HDACs partially restored H3K27ac, but it was not sufficient to rescue the levels of RNAP2-Ser5ph (Extended Data Fig. 5d).

In summary, both intact and ruptured micronuclei exhibit transcriptional defects and chromatin alterations. The correlated acquisition of altered chromatin states raised the possibility that the transcription defects could be inherited.

## Heritable transcription defects

**Fig. 2:**
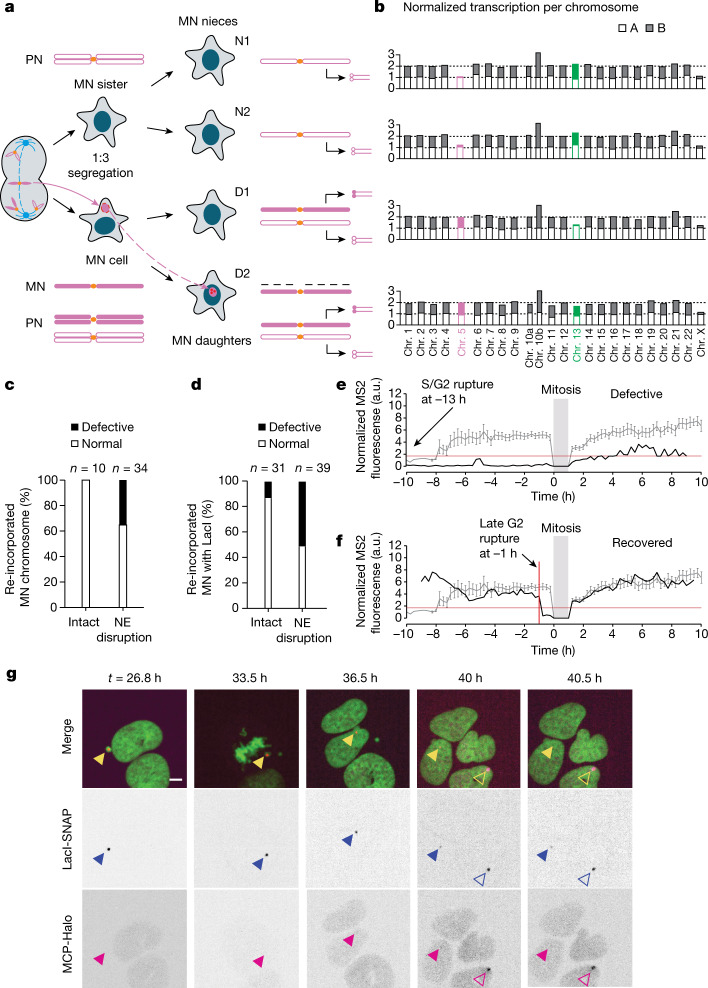
Variably penetrant memory of MN chromosome transcription compromise after re-incorporation into a normal nucleus. **a**, Example of copy number and transcriptional yield after two generations following a 1:3 mis-segregation in generation 1. The MN sister cell generates two monosomic MN nieces (N1 and N2), whereas the MN cell generates MN daughters (D1 and D2). Only one MN daughter is trisomic because the MN chromatid is poorly replicated. See Extended Data Fig. 2a for the outcome of 2:2 mis-segregations. **b**, Near-complete loss of transcription of a re-incorporated MN chromosome 5 (magenta) after a 1:3 mis-segregation in generation 1. Shown are the normalized transcription yields as in Fig. 1b. Chromosome 13 (green) underwent a 2:2 mis-segregation in generation 1 and displays transcription recovery after re-incorporation. See also Extended Data Fig. 7. **c**, Transcription output of 44 re-incorporated MN chromosomes from 37 families using Look-Seq2. Defective indicates a significant reduction in the transcriptional yield (Methods, Supplementary Table 2 and Extended Data Fig. 6). **d**, Transcription status of the U2OS 2-6-3 transcription reporter (*n* = 70 from 13 experiments). Defective indicates little or no visible MCP–Halo signal. **e**, Example of defective MN chromosome transcription after re-incorporation. Grey line indicates the mean and s.e.m. of the FI in controls (normal nuclei; *n* = 23 LacI reporters; Extended Data Fig. 8a). Red horizontal line indicates minimum detectable value in the controls. Black line indicates reporter transcription in a ruptured MN (no detectable generation 1 signal) that does not reach a normal level after re-incorporation (generation 2). **f**, Example of full transcription recovery, analysed as in **e**. Red vertical line indicates the time point of MN nuclear envelope rupture. **g**, Best single focal plane confocal images from a time-lapse series showing defective transcription after re-incorporation. Green, GFP–H2B; blue triangles, reporter locus; magenta triangles, MS2 reporter expression; open arrowheads, cell that enters the field, providing an adventitious MCP–Halo bleaching control. Scale bar, 5 µm. Source Data

**Extended Data Fig. 6:**
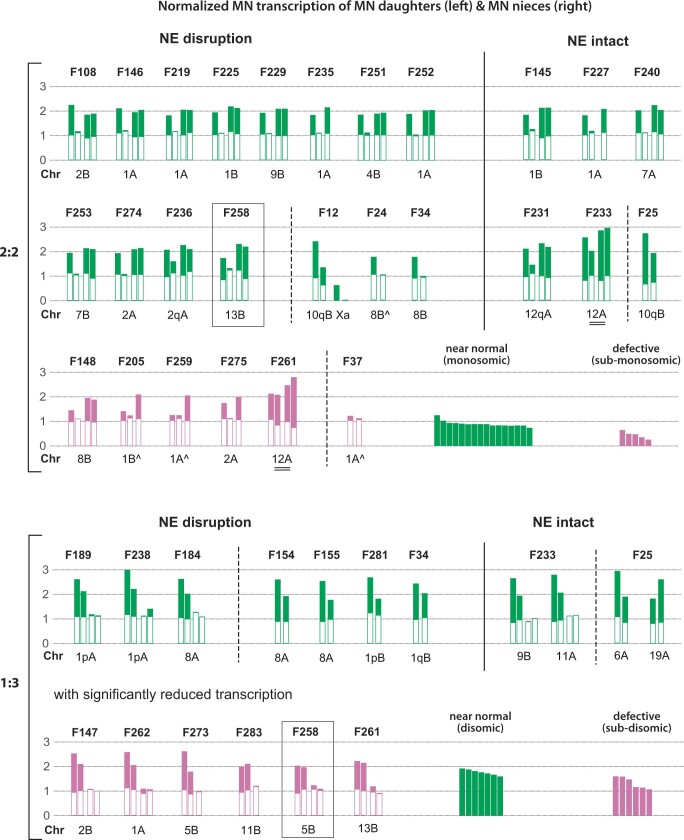
Summary of the transcriptional yield of reincorporated MN chromosomes in all generation 2 families. Each bar plot shows the transcriptional yield of both homologues of the MN chromosome (filled for the MN homologue that is annotated below each plot; open for the intact homologue) in the MN daughters (two on the left) and one or both MN nieces (on the right) in a family. Families are grouped based on the status of MN nuclear envelope integrity (left: NE disruption during generation 1 interphase; right: intact NE) and the segregation pattern of MN chromosomes (top, 2:2; bottom 1:3). Nine families (NE disruption: F12, F24, F34, F37, F154, F155, F281, F34; intact NE: F25) without MN nieces are shown separately from the remaining families with MN nieces. MN chromosomes with near normal transcriptional yield are shown in green: Under a 2:2 segregation, the MN haplotype displays a transcriptional ratio of 1:0 between the MN daughters and normal (monosomic) transcription in the MN nieces; under a 1:3 segregation, the MN haplotype displays a transcriptional ratio of 2:1 between the MN daughters and complete transcriptional loss in the MN nieces. (See Extended Data Fig. 2a for the segregation patterns.) MN chromosomes with significantly reduced transcription are shown in magenta. For these chromosomes, the MN haplotype shows a statistically significant lower transcription than normal transcription (monosomic transcription under 2:2 segregation and disomic transcription under 1:3 segregation, two-sided *z*-test). In families F24, F205, F259, and F37, we identified transcription of the MN haplotype in both daughter cells that is consistent with chromosome fragmentation; we combined the transcriptional yield in both MN daughters in these samples to assess the normality of transcription of reincorporated MN chromosomes. All MN chromosomes with deficient transcription are associated with NE disruption. The summary bar charts of normal and deficient transcription in 2:2 segregation samples and 1:3 segregation samples only include MNs with the predicted patterns of transcriptional imbalance. Deviations from the predicted patterns are explained below. In family F233 and F261, the presence of an extra copy of the Chr.12A homologue in all family members indicates a pre-existing duplication of Chr.12A that is a frequent alteration in RPE-1 cells. In family F12, we inferred the MN chromosome to be the active X (the transcription yield of the inactive X is not shown) that also contains a duplicated 10q segment. In seven families (F236, F231, F25, F189, F238, F281, F34), we inferred that the MNs contain only a chromosome arm; in family F238, we inferred the 1p arm was reincorporated into one MN daughter and the 1q arm was persistent in a MN niece cell based on live-cell imaging. We note that for MN chromosomes that underwent 1:3 segregations, the normality of transcription is assessed by comparing the level of MN haplotype-specific transcription to the level of total transcription of normal disomies (2). The proportional gain of transcription of duplicated homologue (2) is verified using observations from 18 spontaneous trisomies.

After cell division, around 40% of MN chromosomes are incorporated into newly formed daughter cell nuclei (generation 2; Fig. 2a). To determine whether the transcription defects in MN chromosomes can persist even in a normal daughter cell nuclear environment, we performed Look-Seq2 analysis on 37 pairs of daughter cells with re-incorporated MN chromosomes (generation 2, termed MN daughters). The average time interval from chromosome re-incorporation until cell isolation was 16 h, which substantially exceeded the time required for normal chromosomes to recover transcription after mitosis (about 90 min)^28–30^. We also sequenced one (7 out 37) or both daughters (22 out 37) of the MN sister cell (MN nieces). The MN nieces provide the same information about the segregation of the MN chromatid as the generation 1 sister cell and, when it was possible to isolate both nieces, provide the information in biological replicate (Extended Data Fig. 2a). Eight of the 37 MN daughter pairs were processed using our old capture method and lacked contemporaneous isolation of the nieces. We were nevertheless able to infer the transcription status of the re-incorporated MN chromosome based on patterns observed in the samples with MN nieces (Methods, Supplementary Table 2 and Extended Data Fig. 6).

**Extended Data Fig. 7:**
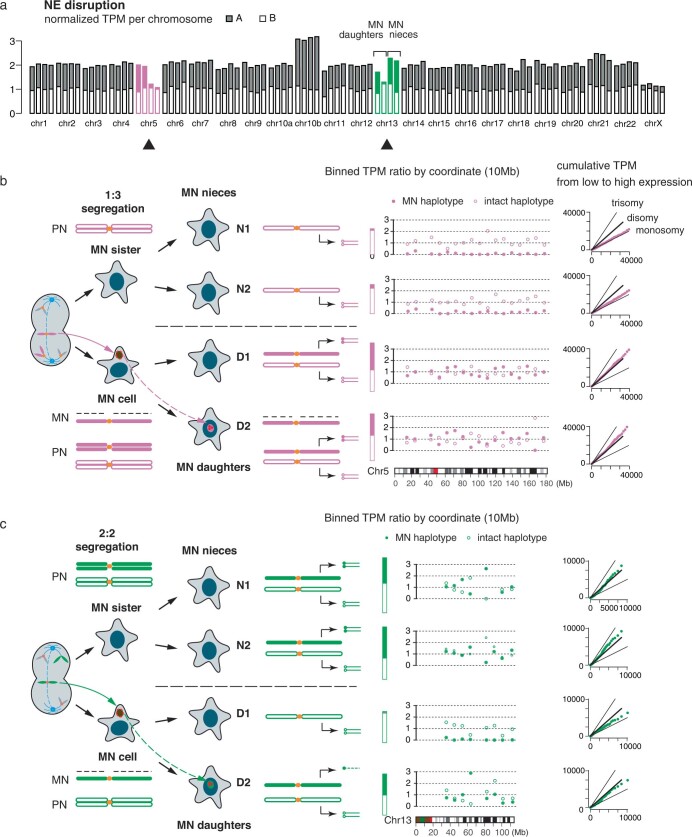
Complete data of the F258 family. Data are for the family shown in Fig. 2b. (a) Haplotype-specific chromosomal transcriptional ratios showing non-disomic transcription of Chr.5 (magenta) and Chr.13 (green). The first two cells are the MN daughters; the second two are MN nieces. Regional transcription data of Chr.5 and Chr.13 are shown in (b) and (c). (b) The segregation pattern, expected transcriptional yield, and observed transcriptional levels of Chr.5 in all four cells. The presence of monosomic expression in both nieces and disomic/biallelic expression in both MN daughters indicate a 1:3 segregation of Chr.5. As the two MN daughters both display close to disomic transcription but one or both of them have reincorporated the Chr.5 copy from the micronucleus, we conclude that the reincorporated Chr.5 is not actively transcribed. (c) The segregation pattern, expected transcriptional yield, and observed transcriptional data of Chr.13 in all four cells. In contrast to the pattern of Chr.5, the two nieces both display disomic/biallelic expression, and one MN daughter displays monosomic expression; this pattern establishes a 2:2 segregation of Chr.13. The presence of transcripts phased to the MN haplotype (filled green circles in the bottom cell) indicates transcription of the reincorporated Chr.13 in the bottom cell. We note that there is more regional transcription variation in Chr.13 than in Chr.5 that is partially due to the lower gene density on Chr.13.

There was heterogeneous transcriptional recovery of the MN chromosomes that were re-incorporated into daughter cell nuclei. Among 44 re-incorporated MN chromosomes, 12 (27%) exhibited a significant reduction or near-complete loss of transcription (Fig. 2b,c, Extended Data Fig. 6 and Supplementary Table 2). Reduced transcription cannot be explained by interspersed DNA losses associated with chromothripsis because transcriptional reduction seemed to be uniform across the chromosome (Extended Data Fig. 7). Moreover, we inferred reciprocal distributions in fragments of the MN chromosome into both MN daughters in four cases and calculated the transcriptional output of the re-incorporated MN chromosome as the combined transcription from both daughters. In 3 out of 4 cases, the combined transcription of re-incorporated fragments still showed a significant reduction (normalized transcriptional output of about 0.18–0.38).

These single-cell transcriptome data indicated that a subset of MN chromosomes acquire heritable transcription defects. The frequency of this defect was probably underestimated because we assessed transcription averaged across 10 Mb bins and were not able to detect transcriptional aberrations at the level of individual genes. We developed single-cell imaging approaches to both verify and further study these heritable defects in transcription.

## Visualization of nascent transcripts

**Extended Data Fig. 8:**
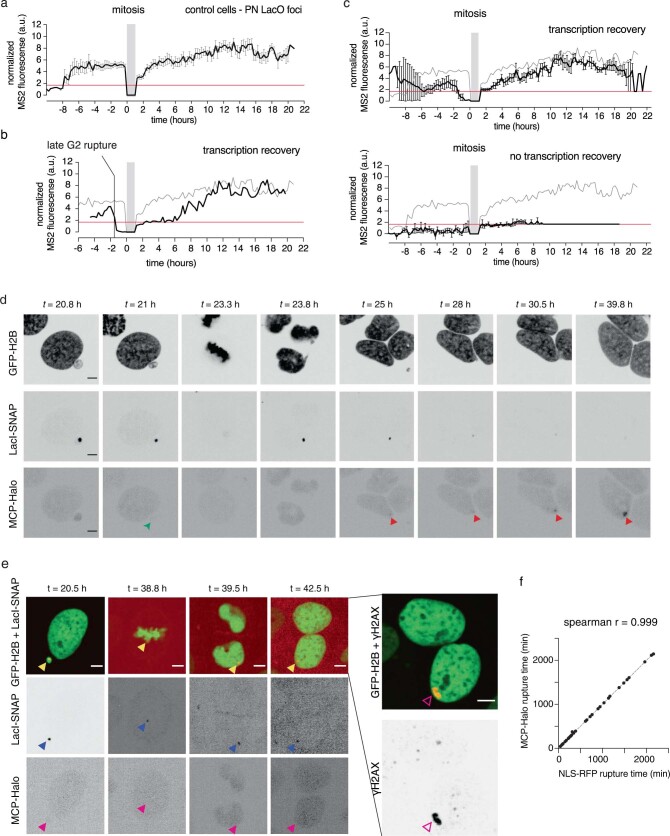
Analysis of nascent transcription from reincorporated micronuclei. (a) Control U2OS 2-6-3 reporters to assess nascent transcription of normally expressing reporters in the main nucleus. Normalized FI of MS2 signal (MCP-Halo) were measured from reporters that were in the main nucleus during both generation 1 and 2 (*n* = 23 LacI reporters). Grey bar: mitosis. Error bars: mean +/− SEM). Red line: minimum detectable normalized MS2 value of the controls (see Methods). (b) Example of a MN with late G2 rupture in generation 1 that recovered transcription after reincorporation into a daughter nucleus in generation 2. Performed and analyzed as in (a), above. Grey line: mean intensity of the control reporters in main nucleus. (c) Aggregated data of nascent transcription from reincorporated MN assessed by the U2OS 2-6-3 reporter, similar to (a) and Fig. 2e and f. Normalized FI of the MS2 signal (MCP-Halo) were measured from reporters that were in a MN in generation 1 and then incorporated into a daughter nucleus in generation 2. Top, a subset of cells with MN that ruptured during generation 1 interphase and then recovered transcription after reincorporation into a daughter nucleus in generation 2 (*n* = 7 analyzed out of 19 similar cases). Note that prior to mitosis there is variable MS2 signal because of variable MCP-Halo accumulation in intact micronuclei and because of variability in the timing of MN NE rupture. Bottom, aggregate data for a similar subset of samples where the MN ruptured during generation 1 interphase and then displayed a generation 2 transcription defect after reincorporation (black line, *n* = 9 analyzed out of 20 similar cases). Note: (1) for ease of visualization, error bars (mean +/− SEM) are shown only for the experimental samples, but not the controls; (2) when there was no detectable MS2 signal in an experimental sample, we assigned the minimal detectable normalized value in control cells (1.7, see Methods) to this sample (This explains the complete overlap between the black and red lines after the 10-hour timepoint). (d) Images from a timelapse series for the experiment in (b), above. Green arrowhead: MN rupture. Red arrowheads: MS2 expression from the reporter after reincorporation into a daughter nucleus in generation 2. Time: hours post release from the G2 block. Scale bars 5 µm. (e) Validation that MN-bodies originate from MN chromosomes, using same-cell live/fixed imaging. Left, images from a time-lapse series (U2OS 2-6-3 system, see Figs. 2e–g, 4d). A cell with a MN harboring Chr.1 (with the reporter integrated in Chr.1p, yellow arrowheads) was identified. The MN ruptured in the interphase that it was formed. After mitosis, the MN chromosome was reincorporated into a daughter cell PN (blue arrowheads, LacI-SNAP) but was not expressed (magenta arrowheads, MCP-Halo), even though it was in a normal nuclear environment. Right, at the end of the time-lapse imaging (t = 42.5 h) cells were fixed and the same cells were analyzed by immunofluorescence microscopy, revealing a large γH2AX-positive MN-body is at the location of the reincorporated MN chromosome identified by LacI-SNAP (open magenta arrowheads). Scale bars 5 µm. (f) Validation of the method to assess MN rupture with the U2OS 2-6-3 reporter system. For all experiments with this reporter, loss of the general nuclear MCP-Halo signal (MCP-Halo contains an NLS) was used to determine the time of MN NE rupture. We verified that MCP-Halo signal loss from MN corresponds to RFP-NLS by two-color live-cell imaging in U2OS 2-6-3 cells expressing both MCP-Halo and RFP-NLS (*n* = 40 from four experiments; two-tailed Spearman’s correlation). Source Data

We adapted the U2OS 2-6-3 nascent transcription reporter system^31^ to assess the transcriptional activity of re-incorporated MN chromosomes. The 2-6-3 transcription reporter construct contains lac operator arrays, which enabled visualization of the reporter locus on chromosome 1. The reporter also contains an inducible mRNA containing MS2 aptamers, which enabled visualization of inducible nascent transcripts. We induced random micronucleation in this cell line, and cells with micronuclei containing the chromosome 1 reporter were identified by imaging (under these conditions, the most frequently mis-segregated chromosome is chromosome 1 (refs. ^18,32^)). After the division of these micronucleated cells, we identified examples of chromosome 1 re-incorporation into daughter cells. Transcriptional activity of the reporter locus was assessed qualitatively by measuring the presence or absence of the MS2-containing transcript (Fig. 2d and Supplementary Video 3). We also quantitatively assessed transcriptional activity by measuring the FI of marked nascent transcripts using an automated time-lapse image analysis pipeline (Fig. 2e,f, Extended Data Fig. 8 and Methods).

Consistent with our Look-Seq2 data, imaging of nascent transcripts confirmed that a subset of re-incorporated MN chromosomes (24 out of 70 live-imaging movies following 2 generations) exhibited persistent defects in transcription (Fig. 2d,e,g and Extended Data Fig. 8). Furthermore, 83% (20 out of 24) of examples exhibiting a generation 2 transcriptional defect had undergone rupture of the micronucleus NE in generation 1, during the interphase of the previous cell cycle (Fig. 2d,e,g, Extended Data Fig. 8d and Supplementary Video 3).

## Transcription defects and DNA damage

Previous studies have shown that DNA damage responses can trigger transcriptional silencing^33–35^. We therefore considered the possibility that heritable defects in the transcription of MN chromosomes might be linked to DNA damage.

**Extended Data Fig. 9:**
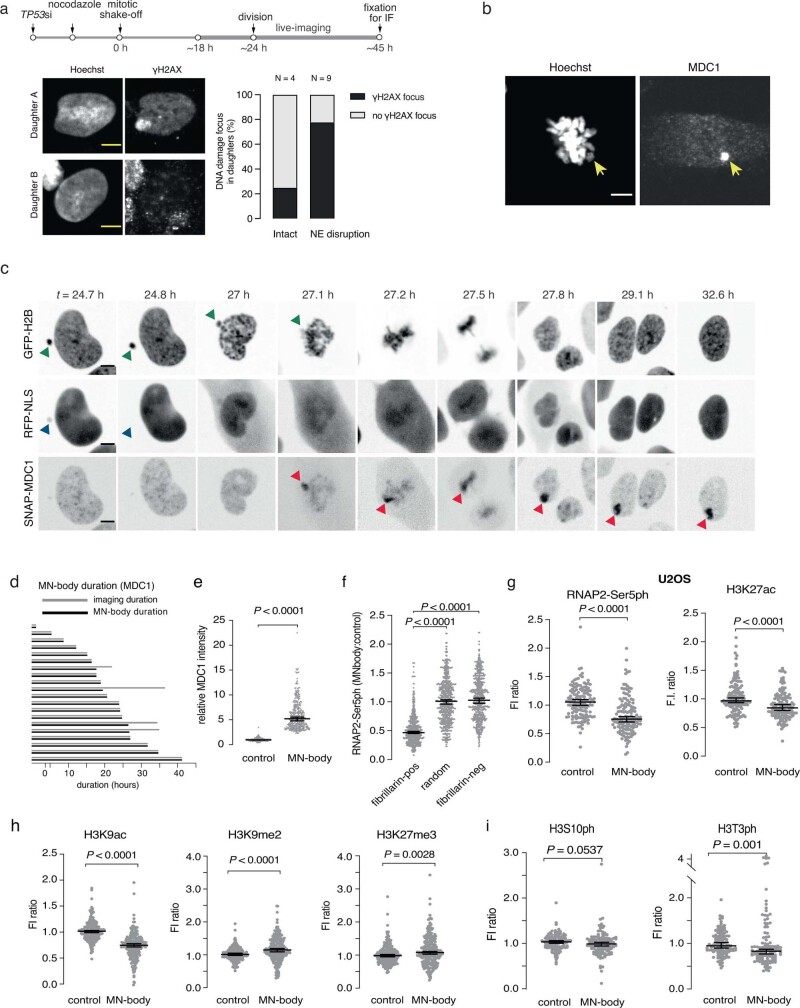
Characterization of MN-bodies. (a) Same-cell live-fixed experiment supporting the fixed imaging shown in Fig. 3a, b. Top, scheme of the experiment. MN were induced in RPE-1 cells and the MN fate was tracked with GFP-H2B and RFP-NLS (to visualize MN NE rupture in generation 1). After most cells progressed into generation 2, they were fixed and labeled to detect γH2AX. Bottom left: representative images of a daughter cell pair, one with and one without an MN-body. Bottom right: summary of 13 cell pairs tracked and analyzed by the same-cell live-fixed experiments (from two experiments). Scale bars 5 µm. (b) MDC1 accumulation on a mitotic chromosome in an RPE-1 cell that had a micronucleus in the prior interphase (generated by nocodazole block and release), shown by immunofluorescence staining of endogenous MDC1 (representative images from two experiments). Note that the micronuclear chromosome can be identified because it is decondensed, a known feature of mitotic micronuclear chromosomes. Scale bar 5 µm. (c) Images from a timelapse series tracking damaged MN chromosomes through cell division and MN-body formation. GFP-H2B: chromosomes; green arrowheads: MN chromosome; RFP-NLS: NE integrity; blue arrowheads: MN NE rupture; red arrowheads: SNAP-MDC1-marked MN DNA damage. Time: hours post release from the nocodazole block for MN induction. Scale bars 5 µm. (d) Durations of MN-bodies assessed by live-cell imaging of SNAP-MDC1 indicate MN-bodies persist throughout most of the generation 2 interphase. Each row shows the lifetime of a MN-body (black bar) and the duration of imaging (light grey bar). In all but four cases, the MN-bodies persisted until the end of the imaging (see Extended Data Fig. 9c for an example of a time lapse series). Note that analysis of the live-cell imaging experiments showed that 68% of cells with MN-bodies were derived from mother cells with a micronucleus that ruptured, 22% were derived from mother cells with intact micronuclei and 10% from non-micronucleated mother cells. (e) Distribution of signal intensities for MN-body by immunofluorescence staining for the endogenous MDC1. Performed and analyzed as in Fig. 3c (*n* = 341, from two experiments). Median with 95% CI. Two-tailed Mann–Whitney. (f) Determination of the background nuclear RNAP2-Ser5ph signal in nucleoli. We measured the background RNAP2-Ser5ph signal in nucleoli (fibrillarin positive), which should lack active RNA polymerase II, and in nuclear regions lacking nucleoli. These values were then normalized to the density of fluorescence intensity from a nuclear mask excluding the nucleoli. The detection of measurable RNAP2-Ser5ph signal in the nucleoli means that we likely underestimate the extent of RNAP2-Ser5ph signal loss in MN-bodies (see Methods; *n* = 650, from two experiments). Median with 95% CI. Kruskal-Wallis with Dunn’s multiple comparisons test. (g) Verification of low transcription and H3K27ac loss in MN-bodies in U2OS cells. Performed and analyzed as in Fig. 3c (*n* = 138, from two experiments). (h) Reduced H3K9ac (left) but not H3K9me2 (middle) or H3K27me3 (right) in MN-bodies. Performed and analyzed as in Fig. 3c (*n* = 222, 234 and 244, left to right, from two experiments). (i) H3S10ph and H3T3ph levels show no increase but a minor decrease in MN-bodies compared to the control. Performed and analyzed as in Fig. 3c (*n* = 130 left; *n* = 124 right, from two experiments). Source Data

As an initial test of this hypothesis, we used a correlated live-cell same-cell fixed imaging protocol^11,26^ to follow MN chromosomes through cell division, observed their re-incorporation into a normal nucleus and detected γH2AX-marked DNA damage by immunofluorescence imaging (Methods). Using live-cell imaging of GFP–H2B signals, we followed the division of 13 micronucleated cells that had re-incorporation of the MN chromosome because neither daughter cell had detectable micronuclei. In 8 out of 13 of these cell divisions, we observed large γH2AX-labelled subnuclear territories that were typically restricted to one of the two daughter nuclei (Extended Data Fig. 9a). We term these structures MN bodies.

To determine whether these γH2AX-labelled MN bodies are derived from re-incorporated MN chromosomes, we used live-cell imaging of the γH2AX-binding protein mediator of DNA damage checkpoint 1 (MDC1) fused to a tag that can be visualized with a fluorescent dye (SNAP-tag). The SNAP–MDC1 fusion protein was not visible on cytoplasmic MN chromosomes during interphase, presumably because it is sequestered in the main nucleus. However, after mitotic NE breakdown, some MN chromosomes were brightly labelled, which enabled us to track them from mitosis into the next interphase (Extended Data Fig. 9b,c and Supplementary Videos 4–6). After division, 31 out of 69 of these chromosomes were incorporated into normal daughter nuclei to become nuclear MN bodies (Extended Data Fig. 9c). We independently confirmed that damaged MN bodies originated from re-incorporated micronuclei using the U2OS 2-6-3 reporter system (Extended Data Fig. 8e). Notably, the DNA damage detected in MN bodies persisted for an extended period (average of >21 h; Extended Data Fig. 9d), longer than the normal time course of DNA double-strand break repair^36^.

**Fig. 3:**
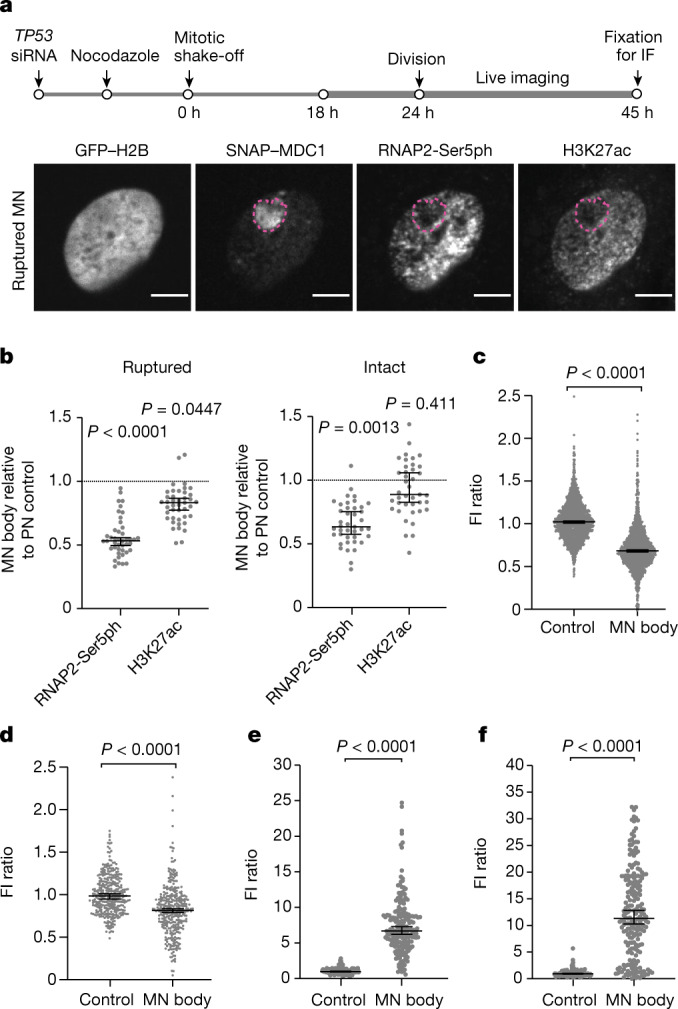
MN bodies exhibit transcription defects and extensive DNA damage. **a**, Defective transcription and H3K27ac in MN bodies. Top, scheme of the experiment (time points are approximate). IF, immunofluorescence imaging. Bottom, representative images of a daughter cell with a MN body from a ruptured micronucleus. Magenta dashed lines indicate a MN body with low RNAP2-Ser5ph and low H3K27ac levels. Scale bars, 5 µm. **b**, Aggregate data of relative MN body fluorescence intensities (FI) for RNAP2-Ser5ph and H3K27ac as in **a** (left to right, *n* = 43 and 41 from 8 experiments). Boxes are median with 95% CI; *P* values from two-tailed Mann–Whitney test comparing the FI ratio between MN and control PN region in the same cell. **c**, Decrease in RNAP2-Ser5ph in MN bodies verified by fixed imaging. Cells were fixed approximately 45 h after mitotic shake-off. MN bodies were identified on the basis of the endogenous MDC1 signal. Data points represent relative FI of RNAP2-Ser5ph in MN bodies against control regions (*n* = 1,447 from 12 experiments). Boxes are median with 95% CI; two-tailed Mann–Whitney test. **d**, Decrease in H3K27ac in MN bodies (*n* = 341 from 2 experiments). **e**, DNA damage in MN bodies. FI measurements of γH2AX intensity (94% of MN bodies were positive, >3 s.d. above the mean of the corresponding nuclear background; *n* = 195 from 2 experiments). **f**, 53BP1 accumulation within MN bodies as in **e** (82% of MN bodies were positive for 53BP1; *n* = 211, from two experiments). Analyses in **d**–**f** are similar to **c**. Source Data

Same-cell live-fixed imaging showed that damaged MN bodies exhibited reduced levels of both RNAP2-Ser5ph and H3K27ac (Fig. 3a–d and Extended Data Fig. 9e–g). MN bodies accumulated γH2AX and endogenous MDC1 as well as the DNA damage response protein 53BP1 (94% of MN bodies were positive for γH2AX and 82% were positive for 53BP1; Fig. 3e,f and Extended Data Fig. 9e). The formation of damaged MN bodies correlated with micronucleus rupture in the previous interphase. However, there were examples of MN bodies derived from micronuclei that remained intact until mitotic NE breakdown (25%, 7 out of 28 cases). In these latter examples, DNA damage was probably acquired during mitosis^11^.

We observed a small, but significant, increase in the repressive histone marks H3K9me2 and H3K27me3 in MN bodies (*P* < 0.0001 and *P* = 0.0028, respectively, two-tailed Mann–Whitney test, Extended Data Fig. 9h). The deficiency in active chromatin marks did not correlate with persistence of the mitotic chromatin marks H3S10ph or H3T3ph (Extended Data Fig. 9i). Therefore, the loss of H3K27ac and RNAP2-Ser5ph seem to be the primary features associated with heritable transcriptional defects of MN chromosomes.

**Fig. 4:**
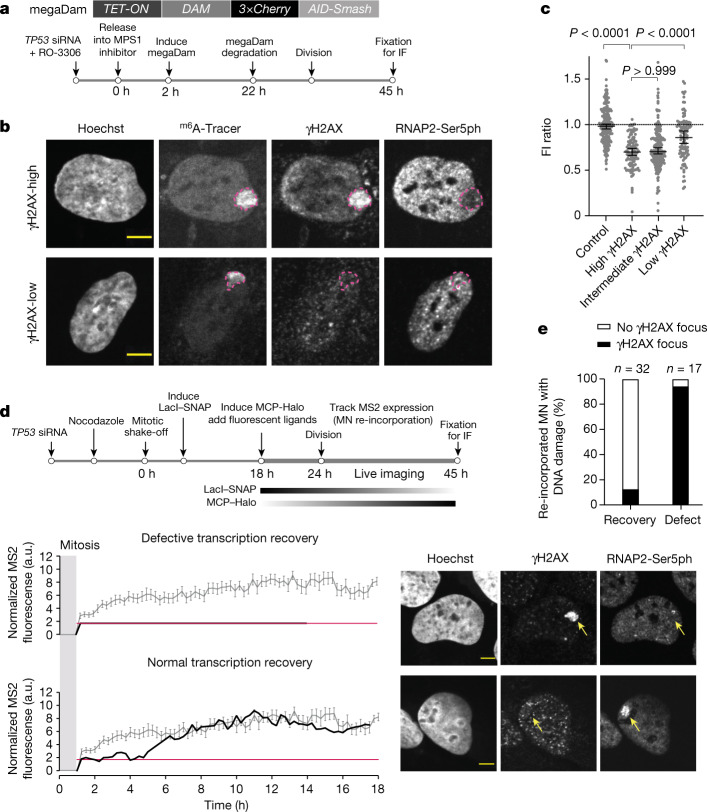
Damaged MN bodies are more likely to have persistent transcription defects. **a**, Transgenerational tracking of MN chromosome fate. Top, cartoon of the gene expressing megaDam. Bottom, scheme of manipulations that restrict DamMN expression to the first interphase when MN form. **b**, Representative images of re-incorporated MN with high (top) and low (bottom) DNA damage in MN bodies. Top, MN body (^m6^A-Tracer, dashed magenta outline) with high γH2AX signal (top quartile, see **c** on the right) but low RNAP2-Ser5ph labelling. Bottom, MN body with low γH2AX signal (bottom quartile) and normal RNAP2-Ser5ph labelling. **c**, Aggregate data of relative RNAP2-Ser5ph intensities in MN bodies with DNA damage levels (Extended Data Fig. 10e; left to right, *n* = 220, 111, 220 and 112 from 4 experiments). Boxes are median with 95% CI; Kruskal–Wallis with Dunn’s multiple comparisons. **d**, Correspondence between high damage level and low transcriptional activity for re-incorporated MN chromosome 1 using the U2OS 2-6-3 nascent transcription reporter. Top, scheme of the experiment. During live imaging, cells with the reporter in a micronucleus were identified by LacI–SNAP and followed through cell division to identify MN body formation. Transcription activity of the reporter was tracked in live cells (MCP–Halo foci) and, after re-incorporation, was scored for γH2AX-marked DNA damage. Bottom left, MS2 signal used to detect reporter transcription activity as in Fig. 2e (grey line indicates the mean and s.e.m. of FI in controls as shown in Extended Data Fig. 8a). Bottom right, after live imaging, cells were fixed to detect γH2AX and RNAP2-Ser5ph (which correlated with the MS2 signal). Shown are representative images. Yellow arrowheads indicate MN bodies. **e**, Summary of the transcriptional output and presence of damage for 49 re-incorporated chromosome 1 with the reporter. The correlation between transcription defect (Fig. 2d) and DNA damage is significant (11 experiments, *P* < 0.0001, two-sided Fisher’s exact test). Scale bars, 5 µm (**b**,**d**). Source Data

**Extended Data Fig. 10:**
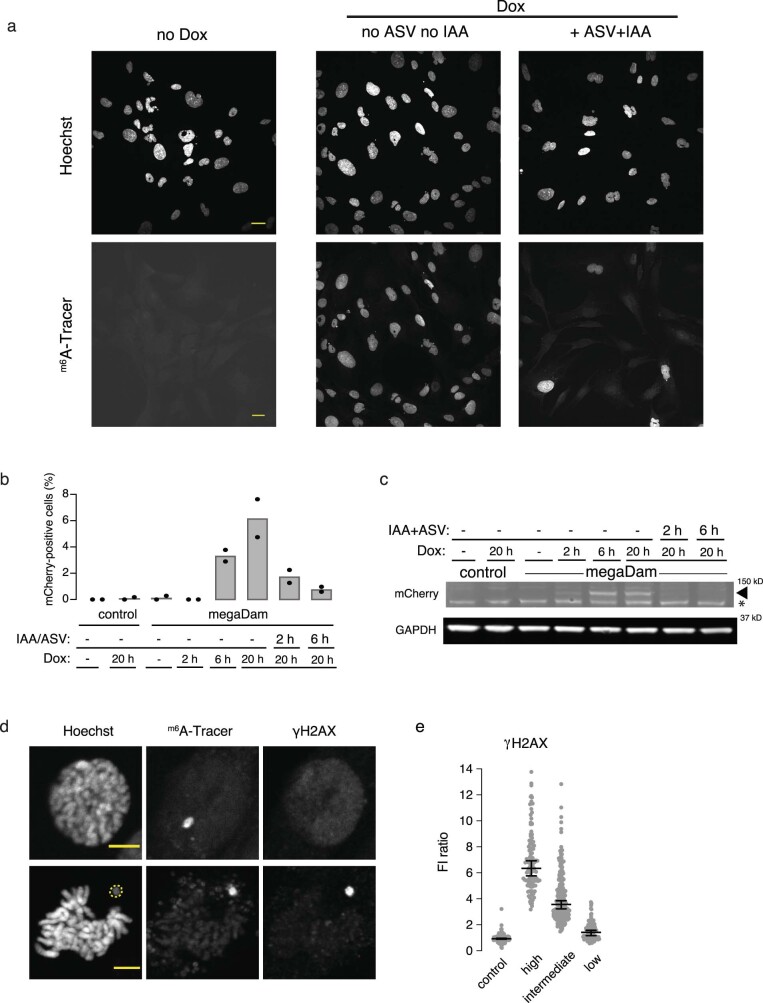
DamMN system characterization. (a) Validation of the DamMN system. Shown are representative single-focal plane confocal images of RPE-1 megaDam cells ~45 h post release from the CDK1-induced G2 block at the start of the experiment (see Fig. 4a and Methods). There is no ^m6^A DNA methylation if megaDam transcript is not induced (left, no Dox); if megaDam is not degraded prior to mitotic entry, all primary nuclei show ^m6^A DNA methylation because of labeling during mitosis (middle, Dox, no ASV no IAA); if megaDam is degraded prior to mitosis because of size exclusion through the NE by passive import, primary nuclei are mostly not ^m6^A methylated (right, Dox, +ASV +IAA). ^m6^A methylation is visualized with the ^m6^A-Tracer (see Fig. 4a and Methods; four experiments). Scale bars 20 µm. Note that even in the condition of degrading megaDam before mitosis (Dox, +ASV +IAA), many cells still show whole nucleus labeling with the m6A-Tracer. This could either result from cells that were in mitosis at the time of megaDam induction or from cells where nuclear exclusion of megaDam was not complete. (b) Efficient induction and degradation of megaDam. FACS analysis to detect mCherry-tagged megaDam. All samples are unsynchronized RPE-1cells with or without megaDam, with or without megaDam transcriptional induction or megaDam degradation for the indicated periods of time. The controls are RPE-1 cells lacking the megaDam construct showing no background autofluorescence without or with Dox treatment. Shown is the percentage of cells expressing mCherry (PE channel, from two experiments). (c) Western blot to detect megaDam for the indicated samples corresponding to the experiment shown in (b), above. Shown is a cropped image of a gel from the region at the megaDam molecular weight (~130 kDa). Note: the a-mCherry Ab detects non-specific background bands, but megaDam is readily distinguished from these background bands (two experiments). * indicates a background band. For gel source data, see Supplementary Fig. 1. (d) Specific labeling of MN chromosomes in mitotic cells (two examples) using the DamMN system. Top shows an MN chromosome in a prometaphase cell lacking DNA damage. Bottom shows an MN chromosome in a metaphase cell with DNA damage. Note that the MN chromosome is less condensed during mitosis, as has been previously described^62^. Performed as described in Fig. 4a. Yellow dashed line: an MN chromosome positive for γH2AX and m6Tracer (*n* = 4 experiments). Scale bars 5 µm. (e) Control for Fig. 4c showing the distribution of MN-body γH2AX FI units relative to the general nuclear background (lacking nucleoli). The MN-body region of interest corresponds to the ^m6^A-Tracer signal (see Methods). The γH2AX low MN-bodies were designated if the total area of γH2AX positive pixels (>3SD above background, see Methods) occupy less than 21% of the MN-body area (corresponding to the bottom quartile of γH2AX positive MN-bodies). The designation of γH2AX intermediate MN-bodies was between 21% and 65.7% of the MN-body area (the middle two quartiles), and γH2AX high was >65.7% of the MN-body area (the top quartile of MN-bodies) (left to right: *n* = 220, 111, 220 and 112, four experiments). Error bars: median with 95% CI. Source Data

The above data show that damaged chromosomes acquire transcriptional defects. However, they do not address whether it is primarily damaged chromosomes that acquire this defect, which would suggest that DNA damage and altered transcription could be mechanistically linked. Testing this association necessitated an imaging system that could track all MN chromosomes, irrespective of whether they are damaged or not. We therefore developed a chromatin tagging system that we refer to as DamMN. DamMN is based on the ability of DNA-adenine methyltransferase (Dam) to methylate adenine residues in DNA, which results in *N*^6^-methyladenine (^m6^A)^37^. An inducible Dam methyltransferase was fused to three tandem copies of mCherry, which restricted it to the cytoplasm because it lacks a nuclear localization signal and is larger than the size-exclusion limit for passive diffusion across nuclear pores (mega-Dam; Fig. 4a and Extended Data Fig. 10a–c). The fusion protein contained two tandem degrons that were used to induce mega-Dam degradation to restrict its expression to the interphase when micronuclei formed. In many G2/M synchronized cells, we could eliminate mega-Dam, which prevented adventitious labelling of all other chromosomes following mitotic NE breakdown (approximately 50% efficiency of specific labelling; Fig. 4a,b and Extended Data Fig. 10a–d).

Using DamMN, we identified MN chromosomes that formed MN bodies. The MN bodies from the top quartile of γH2AX labelling more frequently acquired heritable transcription defects than the MN bodies from the bottom quartile (Fig. 4b,c and Extended Data Fig. 10e). We confirmed this result using the U2OS 2-6-3 reporter system (Fig. 4d,e). Same-cell live-fixed imaging showed that 28 out of 32 cells that recovered transcription lacked detectable DNA damage after MN chromosome re-incorporation. By contrast, 16 out of 17 of the chromosomes that exhibited persistent transcriptional suppression exhibited extensive DNA damage. Therefore, DNA damage and heritable transcriptome defects of MN chromosomes may be mechanistically linked.

## Long-term effects of aberrant nuclei

**Fig. 5:**
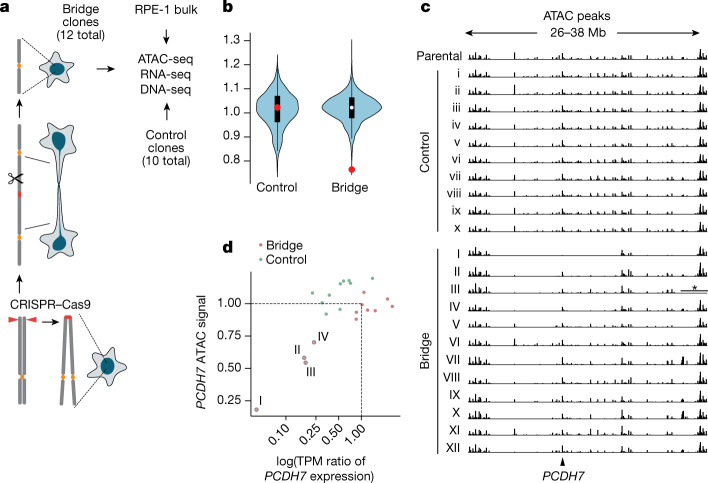
Long-term epigenetic and transcriptional consequences of exposure of a chromosome to the cytoplasm. **a**, Generation of RPE-1 clones with broken chromosome 4 and control RPE-1 clones. Chromosome 4 bridges were generated by sister chromatid fusion after CRISPR–Cas9 breakage at the subtelomeres of either 4p or 4q (left, bottom to top). ATAC-seq, RNA-seq and DNA-seq were performed on the bridge clones, control clones, and bulk (parental population) controls. **b**, Average ATAC signal variation in 10 Mb intervals in control (*n* = 10) and bridge clones (*n* = 12). White dots indicate the median; black boxes indicate first and third quantiles; red dots indicate a 10 Mb region on chromosome 4 (27–37 Mb) with significantly reduced chromatin accessibility in bridge clones. Regions with significantly reduced ATAC intensities in the control clones (fold change < 0.65) are from pericentric regions of chromosome 1, chromosome 9 and chromosome 15 with few ATAC peaks. **c**, Copy number normalized ATAC-seq peak intensities in chromosome 4 (26–38 Mb) in the parental clone, control clones and bridge clones. The region with an asterisk in bridge clone III has DNA copy number zero from homozygous deletion and is masked. See Extended Data Fig. 11 for haplotype-specific DNA copy number alterations and rearrangements in bridge clones. There is a significant reduction in the ATAC signal in 6 out of 12 clones (I–V and XII) (*P* < 0.0001, one-sided permutation test; Methods). Bridge clones are ordered by the level of *PCDH7* expression (ascending), the only gene with significant expression within this region. Arrow indicates the *PCDH7* promoter. **d**, Correlation between *PCDH7* expression (log-transformed transcripts per million ratio) and chromatin accessibility in the promoter and gene body of *PCDH7* (normalized ATAC peak intensity) in control (green dots) and bridge clones (purple dots). Four bridge clones displaying a reduction in both chromatin accessibility and gene expression are on the lower left labelled with sample identifiers.

**Extended Data Fig. 11:**
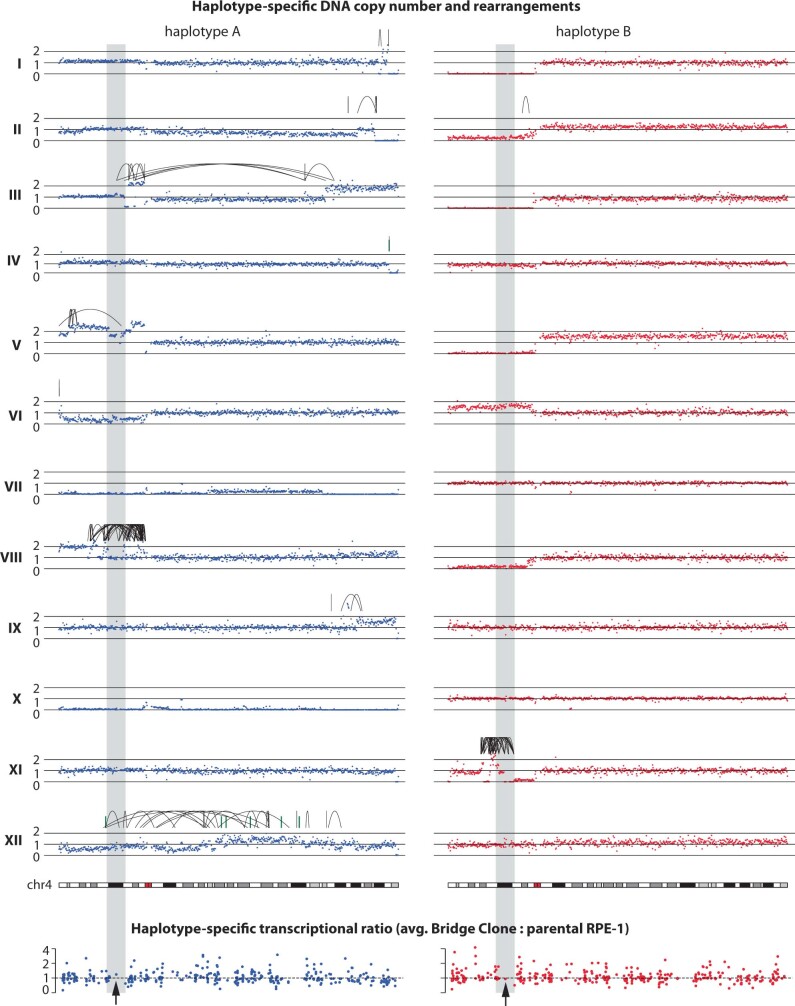
Haplotype-specific DNA copy number, rearrangements, and transcriptional levels of chromosome 4 in 12 bridge clones. Top: Haplotype-specific Chr.4 DNA copy number (250 kb bins) determined from newly generated DNA-Seq data (~5X mean sequencing depth) of the bridge clones. Black arcs represent intra-chromosomal rearrangements determined from previous whole-genome sequencing of the same bridge clones and subclones (10-60X mean depth)^11^. Rearrangements are phased to each homologue based on haplotype-specific copy-number changes at rearrangement breakpoints. The shaded box denotes the region of 27-37 Mb on Chr.4p with reduced ATAC-Seq signal as shown in Fig. 5c. We detected no clonal or subclonal breakpoints in this region (based on both DNA copy-number changepoints and rearrangement junctions) in bridge clones I, II, IV, VI, VII, IX, X. Bridge clone VIII and XI contain the most breakpoints within this region but display no significant change of ATAC density after normalization for copy-number variation. In bridge clone III, ATAC reduction is most prominent between 27 and 30.5 Mb and this region is far away from rearrangements that affect two segments between 32.19-32.23 Mb. Bridge clones V and XII both contain multiple copy-number and rearrangement breakpoints in this region and display modest but significant ATAC reduction (based on the permutation test). Bottom: Haplotype-specific gene transcription (TPM) ratio on Chr.4. Each dot represents the average TPM ratio of a single gene calculated from all 12 bridge clones, excluding samples with complete DNA deletion. Arrows point to the *PCDH7* gene residing in the region with reduced ATAC signal.

To assess potential long-term epigenetic consequences of nuclear aberrations, we analysed samples from a previously described clonal evolution experiment^11^. In this experiment, we had generated chromosome bridges through CRISPR–Cas9-engineered chromosome 4 sister-chromatid fusion^11^ (Fig. 5a). After live-cell imaging, we isolated 12 clones from cells that formed and then broke chromosome 4 bridges (hereafter termed bridge clones). Beneficial for our design, the broken chromosome 4 was preserved after clonal expansion, despite undergoing extensive downstream genetic evolution. We acquired detailed information about the copy number alterations, rearrangements and subclonal architecture of these populations, which was necessary to distinguish epigenetic or transcriptional changes from genetic changes associated with chromothripsis. The DNA copy number was confirmed by re-sequencing of these clones (Extended Data Fig. 11).

**Extended Data Fig. 12:**
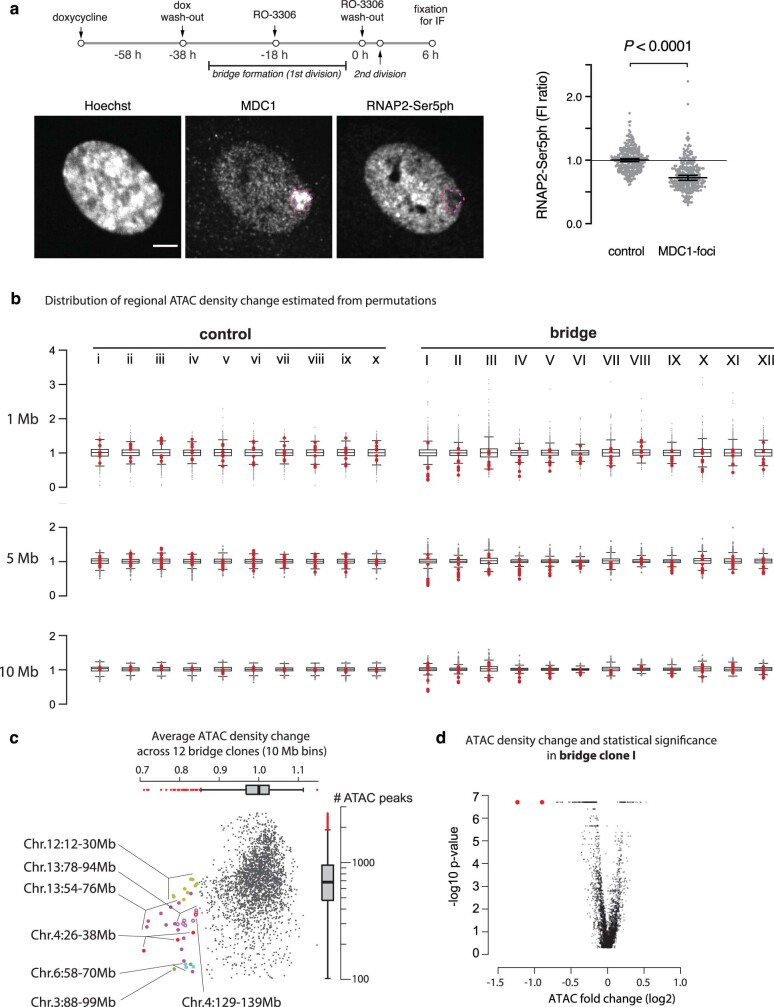
Long-term effects on chromatin and expression after chromosome bridge formation. (a) MN-body-like structures in the daughter cells after chromosome bridge formation. Top, schematic presentation of the experiment. Bottom left, representative images of immunofluorescence analysis for MDC1 and RNAP2-Ser5ph, showing MDC1-positive nuclear bodies (dashed magenta line) after chromosome bridge formation and cell division of RPE-1 cells expressing TRF2-DN (see Methods). We observed a high frequency of cells with MDC1-positive nuclear structures of varying size (~10%) that likely represent reincorporated chromosome bridges. This number is expected, since the frequency of chromosome bridge formation is ~30% per cell division under the conditions described in Methods^11^. Scale bar, 5 µm. Right, quantification of the RNAP2-Ser5ph levels in the MDC1-positive nuclear structures after bridge chromosome reincorporation compared to the PN control. Performed and analyzed as in Fig. 3c (*n* = 309, from two experiments). (b) Genome-wide ATAC signal variation in control (i-x, left) and bridge (I-XII, right) clones in 1 Mb (top), 5 Mb (middle), and 10 Mb (bottom) intervals. The ATAC change in each interval (1 Mb increment) is assessed by normalizing the observed total ATAC signal (only from peaks) in each interval by the mean ATAC density of the null distribution generated by random permutations of individual peaks (see Methods, *n* = 2637 of 1 Mb genomic intervals). Bins with less than 10 ATAC peaks/Mb are excluded. Box plots indicate the first (bottom edge) and third (top edge) quartiles and the median (horizontal line), with whiskers indicating 1.5x the interquartile range. In each plot, red dots represent bins overlapping with the region of 27-38 Mb of Chr.4 that displays the most significant ATAC reduction across all bridge clones (see below). (c) Average ATAC signal variation in 10 Mb intervals across all 12 bridge clones. We only consider 10 Mb regions with 100 or more peaks. As the calculation is performed on all 10 Mb intervals with 1 Mb increment, a single region with a significant reduction in ATAC signal may result in multiple 10 Mb intervals with significant ATAC reduction; these consecutive 10 Mb bins are merged. Bins with the most significant ATAC reduction (fold change < 0.8) mostly come from two regions: Red dots are from the 4p region (26-38 Mb) shown in Fig. 5; purple dots are from a region from Chr.13q (54-76 Mb). Among 10 Mb regions with ATAC signal < 0.85, two are from Chr.4 and Chr.13: Chr.4:129-139 Mb (red circles) and Chr.13:78-94 Mb (purple circles). The other regions with ATAC signal < 0.85 are likely to have a non-epigenetic origin: Two regions (Chr.3:88-99 Mb, green dots; Chr.6:58-70 Mb, blue dots) span centromeres and have low confidence; another region on Chr.12p (12-30 Mb, light green dots) shows a similar reduction in the control clones and the variation is likely related to 12p gain or uniparental disomy that are frequent subclonal alterations in RPE-1 cells. The significant reduction in ATAC signal in Chr.4 and Chr.13 is unlikely to reflect random technical variation as they are specific to the bridge clones. For the Chr.13 region, we do not exclude a biological source for this variation, for example, an unidentified trans signaling effect that is related to bridge formation, breakage, or downstream evolution. It is known that certain genomic regions display more intrinsic variability of ATAC-Seq signals^63^ and such regions may be more prone to effects from chromosome bridge formation or breakage. Box plots indicate the first (bottom edge) and third (top edge) quartiles and the median (horizontal line), with whiskers indicating 1.5x the interquartile range. (d) Scatter plot of the fold change of ATAC signal (log2 transformed) and the *P*-value of ATAC signal variation estimated from permutations in bridge Clone I (two-sided permutation test, up to 5 million permutations without additional adjustment; see Methods). The two red dots are both from Chr.4:27-38 Mb. The cap of p-value at 2 x 10^−7^ reflects 5 million permutations performed for each interval. Source Data

Chromosome bridges are functionally similar to micronuclei^11^. That is, micronuclei and chromosome bridges share the same defect in NE and nuclear pore complex assembly. Moreover, both can undergo NE membrane collapse and expose chromatin to the cytoplasm, and both cause chromothripsis through similar mechanisms^9,11^. We found that broken bridge chromosomes form MN-body-like structures with DNA damage and reduced RNAP2-Ser5ph levels (Extended Data Fig. 12a). In addition to shared functional defects, during clonal evolution, broken bridge chromosomes from one generation often form micronuclei in the next generation and vice versa^11,25^. This means that during downstream evolution, the broken bridge chromosome may be frequently trapped in a secondarily formed micronucleus.

We performed bulk assay for transposase-accessible chromatin with sequencing (ATAC-seq) and RNA sequencing (RNA-seq) analyses on 12 chromosome 4 bridge clones, the parental clone and 10 parental subclones (Fig. 5a and Methods). In general, the ATAC-seq profiles of both control and bridge clones exhibited little variation over 5–10 Mb of genomic intervals (Fig. 5b and Extended Data Fig. 12b) after normalization to the DNA copy number derived from the re-sequencing data (Extended Data Fig. 11). In the bridge clones, however, we identified a variably penetrant but significant reduction in the ATAC-seq signal within a 10 Mb region of chromosome 4p (*P* < 0.0001, one-sided permutation test; Fig. 5b,c and Extended Data Fig. 12c). RNA-seq analysis of the one, non-essential gene in this region, *PCDH7*, verified that the reduction in the ATAC-seq signal across *PCDH7* was associated with a corresponding reduction in its expression (Fig. 5c,d). In bridge clone I, which had the lowest *PCDH7* expression, this region exhibited the most significant and largest fold reduction in ATAC signal (Extended Data Fig. 12d and Methods). In addition to the chromosome 4p region, we identified several regions on other chromosomes with significant reductions in accessibility (Extended Data Fig. 12c).

Because the ATAC peak densities were normalized to the DNA copy number, the reduced ATAC signal on chromosome 4p is independent of DNA loss and therefore reflects reduced chromatin accessibility. The reduction in chromatin accessibility over the chromosome 4p region (27–37 Mb) also cannot be attributed to rearrangements. Three bridge clones with the most significant levels in ATAC signal reduction (clones I, II and IV) had no rearrangement breakpoints on chromosome 4p. Moreover, rearrangements in this region (27–37 Mb) in bridge clone III were restricted to a 30-kb interval (32.19–32.22 Mb) that was far away from the region of the most significant reduction in ATAC signal (Fig. 5c and Extended Data Fig. 11). In addition, bridge clones VIII and XI had the most breakpoints within or flanking the region of 27–37 Mb on chromosome 4p but did not display a significant reduction in ATAC signal or *PCDH7* expression.

The chromosome 4p region may have either been in a bridge or in a subsequently formed micronucleus. Consistent with this notion, the clones with reduced 4p chromatin accessibility either had a 4q-terminal deletion (clones I, II and IV) or had rearrangement breakpoints on both telomeric and centromeric sides of this region (clone III) (Extended Data Fig. 11). Furthermore, two clones (I and III) showed near-complete loss of the B homologue. This result indicated that the reduced accessibility and expression were both on the remaining, rearranged A homologue.

Together, these data suggest that chromatin state alterations acquired in chromosome bridges or micronuclei can, with variable penetrance, be propagated long-term. This effect can occur even in cell culture conditions that lack selection for specific epigenetic changes.

## Discussion

**Extended Data Fig. 13:**
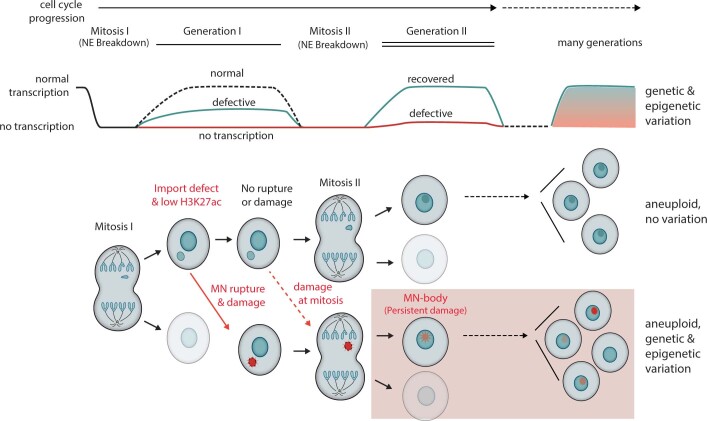
Transcriptional and epigenetic consequences of micronucleation (Model summarizing the results). Top, transcriptional outcomes of chromosomes transiently in micronuclei or chromosome bridges. Green line shows the transcriptional yield of chromosomes in MN without persistent DNA damage (generation 2). Red line shows the transcription yield of MN chromosomes with DNA damage that persists into generation 2. Bottom, cellular events leading to heritable transcription defects. Mitosis I: A cell with a lagging chromosome divides, generating the MN cell and its sister (shaded). Deficient nuclear import of MN prevents the establishment of H3K27 acetylation and causes significantly reduced or complete loss of transcription. If the MN nuclear envelope remains intact and the MN chromosome does not acquire DNA damage during mitosis (top), the MN chromosome can recover transcription after Mitosis II. If the MN chromosome acquires DNA damage either due to MN nuclear envelope rupture during interphase (bottom, red) or subsequently during mitosis (dashed arrow), the damaged chromosome may form MN-bodies with varying degrees of transcriptional silencing (bottom, with red MN-body in the primary nucleus) after Mitosis II. With partial penetrance, transcriptional silencing may persist for multiple generations, generating transcriptional heterogeneity that can be subject to selection.

We established that micronuclei, which are common features of cancer nuclear atypia, can generate heritable defects in transcription. These findings should have relevance for tumour evolution^1–3^ and for contexts during normal development in which micronucleation occurs^38^. We propose the following model for the acquisition of these heritable defects (Extended Data Fig. 13). When micronuclei form, even before NE rupture, they exhibit defects in post-mitotic transcriptional recovery along with variably reduced H3K27ac that probably results from defective nuclear import into micronuclei and the corresponding abnormal composition of the nucleoplasm^26,27^. The reduced levels of H3K27ac persist after micronuclear rupture. However, it can be reversed after the MN chromosome is re-incorporated into a daughter cell primary nucleus, unless the re-incorporated chromosome acquires extensive DNA damage. Persistent DNA damage may have a direct role in repressing transcription because previous work has established that DNA damage or abnormal DNA replication generates transcriptional silencing and/or epigenetic plasticity^33,39^.

There are notable similarities between MN bodies and previously described 53BP1 bodies^40–42^. 53BP1 bodies form during interphase when DNA damage or under-replicated DNA is carried over from the previous cell cycle. Similar to MN bodies, 53BP1 bodies show persistent DNA damage and accumulate a subset of damage response factors. Through incompletely understood mechanisms, 53BP1 bodies are thought to shield DNA lesions until they can be repaired later in the cell cycle^40^. Notably, 53BP1 bodies also exhibit transcriptional suppression, again for unclear reasons and through unknown mechanisms^41^.

There are several ways in which transcription and epigenetic variation from MN chromosomes could be translated into phenotypic variability and long-term epigenetic alterations. One possibility would be that the initial transcriptional alterations are stably and permanently propagated. However, this idea can be excluded because a substantial fraction of MN chromosomes restore transcription after re-incorporation into a normal nucleus. Therefore, the epigenetic alterations from cytoplasmic chromatin are dynamic, although lasting suppression may become fixed in a subset of cases. Indeed, our analysis of cell populations that evolved long-term after breakage of a chromosome 4 bridge identified a large, gene-poor region of chromosome 4p with heterogeneous suppression of chromatin accessibility and transcription. The preservation of altered chromatin only in a gene-poor region makes sense because the clonal expansion was done without selection for any epigenetic or transcriptional change. Without selection, random changes to the normal transcription programme of the cell from random epigenetic alteration of gene expression would compromise fitness, and cells with such alterations should be lost from the population during clonal growth^24^. Non-essential, gene-poor genomic regions are therefore most likely to preserve the footprint of epigenetic changes acquired from initial bridge formation and/or resulting micronucleation. In addition to direct effects on transcription, chromosome-wide transcription silencing may promote evolutionary adaptation indirectly through genetic mechanisms. For example, transcriptional suppression of a trisomic chromosome might allow cells undergoing chromothripsis or other genetic alterations to this chromosome to persist longer in the population, thereby increasing their chance of fixation.

In summary, our results suggest that chromosomal instability is inherently coupled to variation in chromatin state and gene expression through aberrations in the nucleus that are common in cancer.

## Methods

### Cell culture and cell line construction

Cells were cultured at 37 °C in 5% CO_2_ atmosphere with 100% humidity. Telomerase-immortalized RPE-1 retinal pigment epithelium cells (CRL-4000, American Type Culture Collection), U2OS osteosarcoma cells (HTB-96, American Type Culture Collection) and derivative cell lines were grown in DMEM/F12 (1:1) medium without phenol red (Gibco) supplemented with 10% FBS, 100 IU ml^−1^ penicillin and 100 μg ml^−1^ streptomycin. For cell lines with doxycycline-inducible constructs, tetracycline-free FBS (X&Y Cell Culture) was used.

Stable cells lines H2B–eGFP and TDRFP–NLS RPE-1, mRFP–H2B and eGFP–BAF RPE-1, mRFP–H2B RPE-1 and TDRFP–NLS U2OS were generated by transduction of RPE-1 or U2OS cells using lentivirus or retrovirus vectors carrying the genes of interest as previously described^26^. RPE-1 cells with transient expression of a dominant-negative variant of telomeric repeat-binding factor 2 (TRF2-DN)^43^ were treated as previously described^11^. RPE-1 clones derived from single cells with CRISPR–Cas9-mediated telomere loss on chromosome 4 (chromosome 4 bridge) and their derived clones were generated in a previous study^11^. Control parental RPE-1 subclones were generated by FACS and expansion in 96-well plates. The HDAC inhibitor vorinostat (SAHA, Sigma-Aldrich, SML0061) was used at 0.5 μM concentration, as described in the Extended Data Fig. 5d.

#### Generation of cells expressing SNAP-MDC1

The RPE-1 GFP-H2B RFP–NLS SNAP–MDC1 cell line (Fig. 3 and Extended Data Fig. 9) was generated by lentiviral transduction of the SNAP–MDC1-bearing lentiviral vector. This vector was generated by cloning a synthesized SNAPf fragment (sequence from pBS-TRE-SNAPf-WPRE; plasmid 104106, Addgene) with AgeI and BstBI restriction sites into the pLenti CMV/TO GFP-MDC1 (779-2) (plasmid 26285, Addgene, gift from E. Campeau; Genewiz) backbone, substituting SNAPf with eGFP at the N terminus of MDC1. Stably transduced cells were selected by FACS around 10 days after transduction for SNAP–MDC1 expression.

#### Generation of the modified U2OS 2-6-3 transcription system

Our modified U2OS 2-6-3 cells contain GFP–H2B, Cuo-LacI–SNAP and MS2–Halo (Figs. 2 and 4 and Extended Data Fig. 8). These cells were generated from the original U2OS 2-6-3 cells^31^ (gift from D. Spector). In brief, the 2-6-3 transgene consists of 256 tandem copies of the lac operator, which enables visualization of the transgene genomic locus, 96 tetracycline response elements (TREs) to control the reporter transgene and 24 MS2 translational operators (MS2 repeats) for the visualization of the reporter nascent transcript^31^. The 2-6-3 transgene was introduced into a single euchromatic locus on chromosome 1p36 (ref. ^31^). We modified the system as follows. We introduced a lentivirus with the coding sequence of LacI fused to SNAP, under the control of a cumate-inducible promoter. Independent control of LacI–SNAP and the MS2 reporter enabled the identification of the reporter in micronuclei in generation 1, followed by assessment of MS2-marked transcription in generation 2. We also stably introduced genes expressing LacI–SNAP, rtTA and MS2 coat protein (MCP) used for visualizing the MS2 aptamers.

Specifically, U2OS 2-6-3 cells were transduced with pLenti CMV rtTA3 Blast (w756-1, plasmid 26429, Addgene; gift from E. Campeau) for the expression of rtTA, a lentiviral vector, phage ubc nls ha 2×mcp HALO, for the expression of MCP–Halo (plasmid 64540 Addgene; gift from J. Chao) and lenti Cuo-LacI-SNAP, for the expression of LacI–SNAP. Our LacI–SNAP expression vector, CuO-LacI-NLS–SNAPf, contains the coding sequence for the SNAPf-Tag (sequence from pBS-TRE-SNAPf-WPRE; plasmid 104106, Addgene) followed in-frame with the coding sequence for LacI-NLS (sequence taken from Cherry-LacRep; plasmid 18985, Addgene). This sequence was subcloned into pCDH-EF1-CymR-T2A-Puro (QM200VA-1, System Biosciences SBI) using NheI and BstBI restriction sites. The final modified U2OS 2-6-3 cell line was obtained by selection for hygromycin resistance conferred by the 2-6-3 transgene and for blasticidin resistance conferred by the rtTA expression construct, followed by FACS to identify MCP–Halo and LacI–SNAP expression. Note that binding of LacI–SNAP to LacO was transiently inhibited by adding 1 mM isopropyl β-d-1-thiogalactopyranoside before FACS. Transient inhibition was done to avoid genetic instability from LacI binding to the Lac operators, which are a barrier to replication fork progression. Full maps of the constructs used are available upon request. All cell lines used in this study were monitored for mycoplasma contamination.

#### Generation of RPE-1 megaDam cells

The RPE-1 3×Cherry Dam AID Smash cell line (RPE-1 megaDam) (Fig. 4 and Extended Data Fig. 10) was generated by lentiviral transduction of the megaDam vector into a RPE-1 cell line that has a doxycycline-inducible transgene expressing the E3 ligase, OsTIR1, integrated at the ROSA26 locus^44^. The megaDam vector (Fig. 4a) was generated by synthesizing (Genewiz) a sequence containing three copies of mCherry (based on the sequence from pHAGE-EFS-N22p-3XRFPnls; plasmid 75387, Addgene) and the sequences encoding the mAID and SMASh degrons (from ref. ^44^). The Dam coding sequence was taken from TS52_pT_damonly (van Steensel lab). The sequence encoding the Dam–mCherry double-degron fusion was cloned and introduced into the lentiviral vector pCW57.1 (plasmid 41393, Addgene; gift from D. Root, Genewiz). A stably expressed RPE-1 megaDam cell line was obtained by puromycin selection.

### Cell cycle synchronization and methods to generate micronuclei or bridges

To synchronize cells and to generate micronuclei, most experiments in this study used a previously described nocodazole block and release protocol^8,11,26^ unless otherwise stated. In brief, approximately 15 h after *TP53* siRNA (Horizon Discovery) treatment, cells were treated with 100 ng ml^–1^ nocodazole for 6 h followed by a mitotic shake-off procedure. Alternatively (for Figs. 1f and  4a–c and Extended Data Figs. 4f and 10), cells were synchronized at the G2/M border with a treatment of 9 μM RO-3306 (MilliporeSigma), a CDK1 inhibitor, for 18 h. G2/M-arrested cells were next released into mitosis by washing five to seven times with medium, followed by addition of 1 μM NMS-P715 (MilliporeSigma) to impair chromosome segregation through inhibition of the MPS1 kinase^45^.

For the analysis of cells after bridge formation (Extended Data Fig. 12a), RPE-1 TRF2-DN cells were treated as previously described^11^. In brief, the cells were incubated in 0.1 μg ml^–1^ doxycycline (Millipore Sigma) for about 20 h to generate chromosome bridges. Bridges begin to form during cell divisions that occur at least 8 h after the washout of doxycycline. At 20 h after doxycycline washout, cells were synchronized in G2 using 9 μM RO-3306 (Millipore Sigma) for another 18 h. Finally, the cells were fixed and analysed 6 h after release from RO-3306, at the next interphase after bridge resolution and cell division. Data in Fig. 5 and Extended Data Figs. 11 and 12b–d were generated using RPE-1 clones derived in a previous study^11^.

### Detection of nascent transcripts marked with 5-ethynyl uridine

To detect nascent transcripts, cells were incubated for 30 min with 1 mM 5-ethynyl uridine, which was added approximately 23 h after mitotic shake-off from nocodazole release. Incorporation of 5-ethynyl uridine was detected using a Click-iT RNA Alexa Fluor 488 imaging kit according to the manufacturer’s instructions (ThermoFisher Scientific).

### Cas9 RNP transfection

The method for targeting a specific chromosome arm to a micronucleus in RPE-1 cells, complete characterization of the editing efficiency of the sgRNA used in this study and the frequency of generation of micronuclei harbouring the targeted chromosome have been previously described in detail^25^. In brief, a Trueguide Synthetic gRNA system (ThermoFisher Scientific) was used to generate the sgRNA for chromosome 5q with the sequence 5'-G*U*U*GGCCUCCCAAACCACUA-3' (asterisks indicate modified 2′-O-methyl bases with phosphorothioate linkages). RPE-1 GFP–H2B RFP–NLS cells were synchronized in G0 by serum starvation for 23 h and were then transfected with the Cas9–gRNA RNP complexes 22 h after release. Cell synchronization and transfection were performed on cells seeded onto MembraneRing 35 rings (415190-9142-000, Carl Zeiss), which enabled cell isolation by laser capture (see below). Live-cell imaging started 3–5 h after transfection, and cells with micronuclei and their siblings were followed until late G2 phase before cell capture for single-cell RNA-seq (scRNA-seq).

### Live-cell imaging

For the majority of the live-cell imaging experiments, images were collected on Nikon (Ti-E) or (Ti2) wide-field inverted microscopes equipped with Perfect Focus, an environmental enclosure to maintain cell culture conditions (37 °C and humidified 5% CO_2_), a ×20/0.75 NA Plan Apochromat Lambda objective (Nikon) or a ×40/0.95 NA Plan Apochromat Lambda objective, and a Zyla 4.2 sCMOS camera (Andor). At each time point, three 2-μm-spaced *Z*-focal plane image stacks were acquired. Live imaging by confocal microscopy (for the experiments with the adapted U2OS 2-6-3 and MDC1-expressing cells, see below) was performed at 15 min time intervals on a Ti2 inverted microscope fitted with a CSU-W1 spinning disk confocal head (Nikon). At each time point, three 2-μm-spaced *Z*-focal plane image stacks were acquired using a ×40/0.95 NA Plan Apochromat Lambda objective. The microscopes were controlled using Metamorph (v.7.10.2.240; Molecular Devices) or NIS Elements (v.4.30 AR or newer versions; Nikon Instruments).

#### Live-cell imaging for Look-Seq2 experiments

Imaging for Look-Seq2 experiments (Figs. 1 and 2 and Extended Data Figs. 1–3, 6 and 7) was performed using a wide-field inverted microscope and a ×20 objective (see above). Note that at this imaging resolution, we can confidently detect the presence of micronuclei and micronuclear rupture. RFP–NLS or GFP–BAF were used for the assessment of NE integrity of the generation 1 samples as previously described^26^.

We acknowledge, however, that with ×20 wide-field imaging, some events such as micronuclei of small size and borderline cases of NE rupture, fine bridges and rare cases of micronuclei-like structures connected to the PN may not be resolved.

#### Live-cell imaging of cells containing the U2OS 2-6-3 transcription reporter

For live-cell imaging of our modified U2OS 2-6-3 nascent transcript reporter cells (Figs. 2 and 4 and Extended Data Fig. 8), cells were seeded on 35-mm ibiTreat Grid-500 dishes (Ibidi) with a gridded imaging surface after mitotic shake-off. SNAP-tagged and Halo-tagged proteins were labelled using 250 nM JF549-cpSNAP-tag and 50 nM JF646-HaloTag ligands (Janelia Materials) for 15 min before the start of imaging. To induce LacI–SNAP expression, cumate (30 µg ml^–1^; System Biosciences) was added in the medium immediately after the mitotic shake-off step and washed out before imaging. Doxycycline (1 µg ml^–1^; MilliporeSigma) was used to induce expression of the MS2 transcription reporter approximately 2 h before the start of imaging and was maintained in the medium for the remainder of the experiment (Fig. 4d). Confocal imaging started 16–19 h after mitotic shake-off and was performed as described above for about 24 h or until most of the cells of interest had divided and could be imaged in the generation 2 cycle.

#### Live-cell imaging of MDC1-expressing cells

For the live-cell imaging experiments tracking damaged MN chromosomes marked by MDC1 (RPE-1 GFP–H2B RFP–NLS SNAP–MDC1 cells; Fig. 3 and Extended Data Fig. 9), cells were seeded in 35-mm ibiTreat dishes (ibidi) after mitotic shake-off and SNAP-tagged proteins were labelled using 250 nM JF646-SNAP-tag ligand (Janelia Materials) for 15 min before the start of imaging. Imaging was started 16–19 h after mitotic shake-off and images were collected at 15 min intervals with a ×40 objective and 2 × 2 image stitching. An exception was the experiments with high temporal resolution, for which images were collected at 6 min intervals (Extended Data Fig. 9c and Supplementary Video 4) and at 3 min intervals (Supplementary Videos 5 and 6) without image stitching.

### Capture of single-cells for Look-Seq2

The isolation of live cells for scRNA-seq was performed in two ways. Our initial experiments were performed in an analogous manner to our early Look-Seq procedure^8^ for single-cell whole-genome sequencing (which was subsequently refined in ref. ^11^). For these experiments, cell isolation was accomplished by trypsinization followed by FACS of single cells into 384-well μClear imaging plates (Greiner). Micronucleated cells were identified by imaging, and then after the division of these cells, daughter cells were isolated for scRNA-seq by trypsinization and replating into 384-well μClear plates after serial dilution. This procedure was used for a subset of the generation 2 experiments to assess the effect of MN chromosome re-incorporation into normal daughter nuclei (10 out of 127 of the total generation 2 samples; Supplementary Table 1). We subsequently developed the laser capture microdissection (LCM) procedure (Extended Data Fig. 1b, described below) and used this method to collect MN cells and MN sisters (generation 1) and MN daughters and MN nieces (the majority of generation 2 samples) for data presented in Figs. 1 and 2, Extended Data Figs. 1–3, 6 and 7 and Supplementary Tables 1 and 2. The main advantages of our modified system over previous LCM methods^23^ are as follows: (1) minimized cell stress (because the cells are kept in medium in the microchamber setup that prevents the culture to dry out) throughout capturing; (2) higher throughput; and (3) an enhanced ability to capture cell families (again because the cells are maintained in medium throughout capture of multiple cells).

#### Live-cell imaging for Look-Seq2 experiments

Cells were treated as described in ‘Cell cycle synchronization and methods to generate micronuclei or bridges’ for the induction of micronuclei. After mitotic shake-off, cells were handled as described below.

For experiments using the older cell capture method^8,11^, cells were plated into 384-well μClear imaging plates and imaged using wide-field fluorescence microscopy at time intervals of 15 min for up to 48 h or until the majority of cells had progressed through mitosis. For LCM capture experiments, cells were instead plated on MembraneRing 35 rings (hereafter membrane rings; 415190-9142-000, Carl Zeiss) (see details in ‘Development of the modified LCM capture method’ below) and imaging was performed at 15 min intervals as described above, with the difference that image stitching (2 × 2) was used to track mobile cells across different fields of view.

#### Development of the modified LCM capture method

We adapted a previously developed LCM system (Palm Microbeam, Carl Zeiss) and re-designed the capturing and imaging setup as described below and in Extended Data Fig. 1b. A custom-designed aluminium adapter was constructed (SeqTech) to enable imaging of cells plated on the membrane rings. We also custom-designed and 3D printed an adapter using VeroWhite material (opaque white Polyjet resin, SeqTech) to allow placement of the membrane rings in a flipped orientation on the Palm Microbeam LCM microscope with a DishHolder 50 CC (415101-2000-841, Carl Zeiss; Extended Data Fig. 1b). This enabled capturing of cells in a multi-well capture plate that could be placed close to the cells, which helped increase the capturing speed, efficiency and therefore the throughput of the method. The designs of the adapters are available upon request. The Look-Seq2 method is described in detail in a provisional patent^46^. A hydrophobic barrier was applied at the periphery of the surface of the membrane rings using an ImmEdge PaP Pen (Vector Laboratories) to prevent evaporation of the medium. Next, the cells were plated on the membrane rings.

At the time of cell capturing, the cells were supplemented with medium containing HEPES buffer. Next, the membrane rings were flipped upside down and positioned on the custom-made adapter after the application of a glass 20 mm glass coverslip (Neuvitro), which we refer to as the microchamber (Extended Data Fig. 1b). The cells in the microchamber were transferred to a Palm Zeiss LCM microscope on the custom-made adapter. Cells of interest were identified by extrapolation of the coordinates on the imaging microscope to the LCM microscope using a custom MatLab script and reference marks that were applied to the membrane rings. Snapshots of all imaging channels of the cells of interest were taken immediately before LCM to ensure accurate assessment of the micronuclear NE integrity and cell viability (from the maintenance of nuclear RFP–NLS). Cells of interest on small membrane surfaces were then catapulted into single wells in 5.5 μl of lysis buffer (see ‘Generation of scRNA-seq data’ below) in a 96-well capture plate (CapturePlate 96 (D), 415190-9151-000, Carl Zeiss). The cell lysates were quickly transferred to 96-well PCR plates (Eppendorf) by centrifugation and stored in −20 °C for cDNA library generation and scRNA-seq.

### Generation of scRNA-seq data

cDNA synthesis and amplification were performed using a modified protocol of the SMART-Seq v4 Ultra Low Input RNA kit for sequencing (Takara Bio). In brief, the manufacturer’s instructions were used with the following modifications: (1) 3 μl of RNAse inhibitor was added per 20 μl for the 10x reaction buffer solution; and (2) all the reaction volumes were decreased by half to maintain reactant stoichiometry. cDNA amplification of single cells was performed by PCR for 21 cycles and the amplified products were purified using AMPure XP paramagnetic beads (Beckman Coulter).

The quality and quantity of the amplified cDNA libraries were assessed using a dsDNA HS Assay kit on a Qubit fluorometer (ThermoFisher Scientific) and an Agilent High Sensitivity DNA kit on a 2100 Bioanalyzer system (Agilent Technologies). cDNA libraries with concentrations below 0.2 ng µl^–1^ and/or fragment size distributions not showing a peak at 2 kb as expected for the size distribution of full-length mRNAs were excluded from subsequent analyses. Sequencing libraries were generated by tagmentation using a Nextera XT DNA Library Preparation kit (Illumina) with minor modifications of the manufacturer’s instructions. In brief, 0.1–0.2 ng µl^–1^ of cDNA samples were used in one-quarter of the suggested volumes for all subsequent reactions. Barcodes from the Nextera XT Index kit v2 Sets AD (Illumina) were used for multiplexing, and the quality of RNA-seq libraries was assessed using a Qubit fluorometer (ThermoFisher Scientific) and a 2100 Bioanalyzer (Agilent Technologies). Sequencing was performed on MiSeq and HiSeq 2500 sequencing instruments (2× 100 bp) after quantity normalization and additional quality assessment of the individual libraries by low-pass sequencing on a MiSeq Nano flow cell.

### scRNA-seq data processing

The complete workflow of scRNA-seq data processing and downstream analysis was implemented as a snakemake pipeline (publicly available at https://github.com/chengzhongzhangDFCI/nature2023)^47^. Details of individual steps are described below.

#### Alignment and post-alignment processing of sequencing data

Sequencing reads were aligned using STAR (v.2.7.6a) (https://github.com/alexdobin/STAR) to the Gencode v.25 reference (–twoPassMode basic; –quantMode: TranscriptomeSAM and GeneCounts) and sorted by genomic coordinate. For post-alignment processing, we followed the best practice of GATK (https://gatk.broadinstitute.org/hc/en-us/articles/360035531192-RNAseq-short-variant-discovery-SNPs-Indels-), which included adding read group information and executing SplitNCigarReads (both using GATK v.4.1.9.0). We skipped duplicate removal as the estimated fractions of duplicated reads was below 5% for all libraries.

#### Quality assessment of scRNA-seq data

The STAR program outputs various alignment metrics of the RNA-seq data. We report the following information in Supplementary Table 1: the percentages of unmapped reads, multi-mapped reads, reads mapped to no features according to gene annotations, and reads mapped to multiple features according to gene annotations; the number of genes (transcripts) represented by at least 1, 5 or 10 reads; and the average number of reads covering each gene. The primary metric for removing low-quality scRNA-seq libraries was the number of genes covered by ≥5 reads. For control (untreated) RPE-1 cells isolated by FACS, Look-Seq or Look-Seq2 procedures, we excluded cells with <6,000 genes covered by ≥5 reads; for cells with or related to micronucleation, including MN cells, MN sisters, MN daughters and MN nieces, isolated by either Look-Seq and Look-Seq2, we excluded cells with <4,000 genes with 5 or more reads. A total of 464 cells were included in the final analysis, 434 of which were sequenced on HiSeq (Illumina) and another 30 by MiSeq (Illumina). All of these samples are listed in Supplementary Table 1 with annotations of the experimental setup.

### Single-cell gene expression analysis

#### Quantification of total gene expression

We calculated the TPM for each gene using RSEM (https://deweylab.github.io/RSEM/) with rsem-calculate-expression. We excluded genes with low expression (mean TPM in control cells ≤ 25) that displayed more cell-to-cell variability due to both transcriptional noise and technical variation.

#### Quantification of allele-specific expression

We assessed allelic gene expression based on allelic depths of RNA-seq reads at heterozygous sites (only single-nucleotide variants) calculated using the ASEReadCounter module of GATK (v.4.1.9.0). The list of heterozygous variants and the haplotype phase of variant genotypes on parental chromosomes were both taken from a previous study^48^. To calculate the average allelic fraction of transcripts of each gene, we first summed the total number of haplotype-specific (A or B) reads at all variant sites in coding (exonic) and untranslated regions and then calculated the fraction of haplotype-specific read coverage (*f*_A_ and *f*_B_; *f*_A_ + *f*_B_ = 1). The averaging of allelic coverage at the gene level helps to improve the accuracy of allelic fraction calculation when there are multiple variants in the transcribed sequence. To eliminate allelic gene expression bias in parental RPE-1 cells (for example, from imprinting), we only assessed allelic expression of genes with roughly equal allelic contributions from both parental homologues in control RPE-1 cells (average allele fraction in control cells is within the range of 0.3–0.7). Two chromosomes required special treatment. For chromosome X transcripts originating from one normally transcribed active X and one epigenetically silenced inactive X, we included all X-linked genes. To account for the presence of a duplicated copy of chromosome 10q (60.78 Mb-qter on GRCh38) that is translocated to the q-terminus of active X, we adjusted the normal range of allele fractions of the single-copy homologue in the trisomic 10q region to be 0.2*−*0.4. As we had previously determined the allelic identities of both the active X and the extra copy of chromosome 10q, we used the transcription of the active X and the normal (single-copy) chromosome 10 homologue as reference to calibrate the transcription of the inactive X and the trisomic 10q segment.

#### Quantification of transcriptional changes relative to normal disomic transcription

We assessed transcriptional changes using both the total transcriptional yield (measured by TPM) and the allelic fraction of transcripts from each gene (Extended Data Fig. 1c). For the total transcription yield, we calculated the transcription ratio by normalizing the TPM in each single cell by the mean TPM in control RPE-1 cells (listed in Supplementary Table 1). To mitigate variations in the TPM ratios derived from individual genes due to transcriptional noise, we calculated the average TPM ratio in 10 Mb genomic intervals for regional transcription analysis and across entire chromosomes or chromosome arms for chromosome (or arm)-level transcription analysis. As different genes show varying degrees of transcriptional variation, we performed a weighted average to attenuate the contributions of genes with more variability that is due to either transcriptional noise^49,50^ or technical variability^51,52^. This averaging strategy is described in the Supplementary Information. A similar strategy was used to estimate the average allelic fraction. We further introduced a scaling factor for TPM ratios in each cell to eliminate global changes to TPM values due to significant upregulation or downregulation of one or a few highly transcribed genes (Supplementary Information).

The normalized haplotype-specific transcription value was calculated by multiplying the average TPM ratio by the average allele fraction. For normal disomic transcription, the average TPM ratio is 1 and the average allelic fraction is 0.5, thus the average haplotype-specific transcription is 0.5. For monoallelic transcription that is due to DNA loss or complete epigenetic silencing, we expect the TPM ratio to be around 0.5 (assuming a linear relationship between gene transcription output and copy number^24^) and the silenced or lost chromosome to have allelic fraction 0; for trisomies with a 2:1 allelic ratio, we expect the TPM ratio to be 1.5. In both cases, the unaltered homologue will have close to normal allelic transcription (0.5) and serve as an intrinsic control for the altered homologue.

We note that experimental perturbations (for example, nocodazole treatment) can cause gene expression changes that are independent of chromosome-specific transcriptional changes on the MN chromosome. We found that the majority of these differentially expressed genes had both low transcription level and low transcriptional variability in control RPE-1 cells. These genes were excluded from the TPM and allelic fraction calculation by the total TPM cutoff (TPM > 25).

#### Quantification of normal transcriptional variation

We derived a reference distribution of normal transcription of each homologous chromosome from the haplotype-specific transcription in control RPE-1 cells (Extended Data Fig. 1d). These reference distributions were used to assess whether the observed average transcription of a chromosome in a RPE-1 cell is significantly different from normal transcription. Three chromosomes required special treatment. (1) RPE-1 cells frequently acquire alterations to chromosome 12, including trisomic 12, tetrasomic 12p or 12p uniparental disomy. We manually reviewed chromosome 12 transcriptional levels (of both homologues) in the control cells and removed those with chromosome 12 alterations. (2) RPE-1 cells share an extra copy of a 10q segment that is attached to Xa. To match the expression of the single-copy homologue to the mean expression of other autosomes, we multiplied the expression of both homologues by a factor of 1.5. (3) For chromosome X, we similarly multiplied the expression of both Xa and Xi by a constant factor (about 0.6) to match the mean expression of Xa to the mean expression of an autosome. The normalization of chromosome 10q expression and chromosome X expression only affects the visualization of transcriptional changes of individual chromosomes. It does not affect the assessment of whether the observed transcriptional change is within the normal range of variation, which was done separately for each chromosome.

#### Estimation of transcriptional changes due to chromosomal gain and loss

In addition to normal disomic transcription, we estimated the range of normal transcription of monosomies or trisomies to assess the normality of gene transcription after chromosome mis-segregation, micronucleation or re-incorporation of MN chromosomes. For monosomies, we estimated the residual fraction of transcripts from the deleted homologue based on the RNA-seq data of bona fide monosomies—when a pair of daughter cells showed approximately 0:2 allelic ratio. For trisomies, we estimated the range of normal trisomic transcription using three strategies.

First, assuming the average transcriptional yield from each parental homologue to be similar in both trisomies and disomies, we expected the total allelic transcription yield from two copies of the duplicated homologue in a trisomic cell to be similar to the total transcription yield from both homologues in disomic cells. Therefore, we compared the observed allelic transcription yield of the duplicated homologue to the distribution of total transcription (from both homologues) in control RPE-1 cells to assess the normality of transcription of the duplicated chromosome.

Second, assuming the transcription yield of the single-copy homologue is similar to the transcription yield from either copy of the duplicated homologue, we used the allelic ratio between the duplicated homologue and the single-copy homologue in trisomic cells to assess the normality of transcription of the duplicated chromosome. For this comparison, we used the allelic ratios of the trisomic 10q segment in control RPE-1 cells to assess the normality of transcription of spontaneous trisomies.

Finally, we used the transcription data of RPE-1 cells with de novo trisomies either induced by nocodazole treatment or generated spontaneously during cell culture. To identify bona fide trisomies, we used the following three criteria: (1) we required that there was approximately proportional changes in the chromosome-wide average TPM ratio (1.5); (2) we required that the transcriptional allele fractions were consistent with the DNA allelic fractions (1/3 or 2/3); (3) importantly, we required that each trisomy is either shared by a pair of sibling cells or accompanied by a monosomy in another sibling cell, thereby indicating a de novo mis-segregation event. The last requirement is equivalent to a biological replicate and should exclude random transcriptional variation that affects individual cells. Reference monosomies and trisomies are annotated in Supplementary Table 2 (from both generation 1 and generation 2 samples).

One advantage of using de novo trisomies as a reference is that the observed transcriptional changes are not affected by long-term adaptive changes that may occur in clonal trisomies (for example, 10q). We note that even in de novo trisomies, there is a slight decrease in the expression of each DNA copy, which resulted in a transcription ratio slightly lower than 1.5.

#### Classification of chromosomal transcription in single RPE-1 cells

We used haplotype-specific transcription to determine whether the observed transcription yield of each chromosome in a single cell is consistent with a normal RPE-1 genome or indicates gain or loss of transcription due to chromosome mis-segregation (including micronucleation). To classify the transcriptional copy-number state based on haplotype-specific transcription yield, we first compared the average haplotype-specific transcription of every chromosome in a RPE-1 cell to the normal range of transcription (‘reference’) derived from control RPE-1 cells (Extended Data Fig. 2d). The normal transcription distribution (Extended Data Fig. 1d) reflects transcriptional variation of a single chromosome and was calculated separately for each parental chromosome. For chromosomes in which transcription levels were outside the normal range (red dots in Extended Data Fig. 2d), we then compared the haplotype-specific transcription yield to normal disomic transcription or complete DNA loss (‘nullisomic’) to determine whether the transcriptional changes were consistent with whole-chromosome gain or loss. If the transcriptional level of a chromosome did not fall within normal ranges of monosomic (1), disomic (2) or nullisomic (0) transcription states, it was classified as intermediate (1+ or 1–). For the duplicated 10q segment or any chromosome inferred to be duplicated, we only assessed whether the transcriptional level was within the normal range of disomic transcription or displayed significantly reduced transcription.

To assess whether the observed transcription yield of a chromosome is within the range of normal monosomic or disomic transcription, we used two-tailed *z*-tests and considered deviations with Bonferroni-corrected *P* values of ≥0.05 to be non-significant. For the comparison against nullisomic transcription, we did not calculate the *P* value as the transcription yield should be strictly zero (that is, no variation); any deviation from zero reflects technical errors (phasing errors, amplification errors, sequencing errors, among others), for which we did not have sufficient data to estimate the null distribution. We classified a chromosome as being nullisomic if the normalized transcription yield was below 0.1 based on the observations of nullisomic chromosomes in bona fide monosomies. The classification of the transcriptional states of all chromosomes with non-disomic transcription in micronucleation-related cells is summarized in Supplementary Table 2.

#### Identification of mis-segregated chromosomes and chromosomes in micronuclei

We identified mis-segregated chromosomes based on changes in the total and haplotype-specific transcription in all sibling cells from each experiment (family). We first used allele-specific transcription to identify homologous chromosomes with transcription levels significantly deviating from normal (monosomic for that haplotype) transcription (summarized in Supplementary Table 2). We then considered both allelic and total transcription levels across all cells in each family (MN cell, MN sister or their daughters) to determine the integer DNA copy number states of chromosomes with non-monosomic transcription and the chromosome segregation pattern in the family. We also assessed whether the observed transcriptional variation is consistent with the expected outcome of micronucleation, micronucleation-independent mis-segregation that generates reciprocal loss and gain between sibling cells or random transcriptional noise.

The identification of chromosomes that were partitioned into micronuclei is based on matching the allelic imbalance and DNA copy number states of a chromosome in all sibling cells inferred from the transcriptome data to the expected outcomes of different mis-segregation or segregation patterns of the MN chromosome (Extended Data Fig. 2a). This inference automatically determines the parental haplotype of the MN chromatid. Notably, the pattern of micronucleus-related transcriptional changes can be identified independent of the transcription level of the MN chromatid either in the MN cell or in the MN daughter cell that has re-incorporated the MN chromatid. Therefore, the inference of the MN chromatid based on the predicted patterns of transcriptional changes does not affect the assessment of transcriptional normality of the MN chromatid either in a micronucleus or after re-incorporation.

We note that the most definitive features of micronucleus-related transcriptional changes are the loss of transcription of the MN chromatid, which occurs in two scenarios: (1) in the MN sister cell or its progeny (MN nieces) owing to mis-segregation of the MN chromatid; (2) in one of two MN daughter cells that is missing the incompletely replicated MN chromatid in the MN mother cell. In both scenarios, the inference of the MN chromosome relies on the detection of near-complete transcriptional loss in a non-MN cell (MN sister or MN nieces) or in one MN daughter cell that did not re-incorporate the MN chromatid. Therefore, the inference of the MN chromosome is insensitive to both spontaneous transcriptional variability in non-MN cells (as it relies on the detection of complete transcriptional loss) and potential transcriptional changes due to the presence (MN cell) or re-incorporation (MN daughter cell) of the MN chromosome.

We further note a few special cases. First, we identified two MN cell–MN sister pairs (F84 and F206) with no chromosome displaying significant deviations from the normal range of transcriptional variation. We inferred that the MN cell in these two families contained MN chromosomes that had undergone 2:2 segregation and had normal transcription output. Under these circumstances, the MN chromosome is transcribed like a normal chromosome and therefore ‘invisible’ based on the transcriptome data. We nonetheless cannot rule out other possibilities, for example, when the micronucleus contains an acentric chromosome arm (13p, 14p, 15p, 21p or 22p), the transcription output of which cannot be assessed by RNA-seq. The inference of normal MN transcription in these two families reflects a conservative estimate of transcriptional deficiency in micronuclei. Second, in family F71, we identified chromosome 18 to have a 3:1 transcriptional ratio between the MN cell and the MN sister cell. This ratio indicated that the MN cell contained an extra chromosome 18 (due to 3:1 mis-segregation) that is being transcribed to normal levels. The extra chromosome 18 copy could be either contained in the PN or partitioned in the micronucleus; we inferred the extra chromosome 18 copy to be in the PN because we identified chromosome 1p that displays the transcriptional pattern expected for a MN chromosome with defective transcription. Third, there were nine families of MN daughters for which we did not obtain MN niece cells (most of these were collected using the original Look-Seq method). For these cases, we inferred the identity of the MN chromosome by comparing the total and allelic transcriptional imbalance between the MN daughters to the transcriptional profiles of MN daughters for which both the MN chromosome and its segregation pattern can be directly inferred from the data of MN nieces. Specifically, when the re-incorporated MN chromosome displayed normal transcription, the MN daughters showed either about 3:2 or 2:1 transcriptional ratio, which reflected the presence of an extra, normally transcribing chromosome in one MN daughter; we used this information to infer normal transcription of re-incorporated MN when the MN daughters showed the same transcriptional ratios even when no MN niece is available. When the re-incorporated MN chromosome displayed deficient transcription with transcriptional yield *a*, the MN daughters would show transcriptional ratios of either 2 + *a*:2 or 1 + *a*:1. When *a* ≈ 0, the MN daughters showed identical transcription patterns; we can nonetheless conclude that the extra MN chromatid being present in either daughter cell produced no transcriptional output and therefore must be epigenetically silenced. We inferred family F254 to correspond to this scenario.

Finally, we noted that chromosome 18 and acrocentric chromosomes (chromosomes 13, 14, 15, 21 and 22) displayed more transcriptional variability than other chromosomes. The more pronounced variability of these chromosomes is obvious from the reference distributions. Such variation was generally not shared by sibling cells and/or is inconsistent with the patterns of transcriptional changes predicted by micronucleus-related or micronucleus-independent chromosome mis-segregation events. Therefore, the variable transcription of these chromosomes does not pose a problem for the identification of MN chromosomes.

#### Quantification of the transcriptional yield of MN chromosomes

After identifying the MN chromatid (both the chromosome identity and the parental haplotype), we estimated the transcriptional yield of the MN chromatid based on the haplotype-specific transcription yield. For MN cells (generation 1) of 2:2 segregation, they contained a single copy of the MN chromatid in the micronucleus, we therefore directly derived the transcriptional yield of the MN chromatid from the transcriptional yield of the MN haplotype. For MN cells having undergone 3:1 (MN cell: MN sister) mis-segregations, the haplotype-specific transcriptional yield of the MN haplotype represented the combined transcription output from both the MN chromatid and its intact sister chromatid in the PN. In this scenario, we compared the transcriptional yield of the MN haplotype to the transcriptional yield of reference disomic transcription levels to assess whether the MN chromatid displayed normal or deficient transcription.

For re-incorporated MNs, if the MN chromosome was inferred to have undergone a 2:2 segregation in generation 1, then the single-copy MN chromatid is distributed to one or both MN daughter cells. In this scenario, we estimated the transcription yield of the re-incorporated MN chromatid using the combined transcriptional yield of the MN haplotype in both MN daughters (this accounts for possible fragmentation and reciprocal distribution of fragments of the MN chromatid into both daughters). We then compared the transcription yield of the MN haplotype to the range of normal transcription of a single homologue to assess transcriptional normality or deficiency. If the MN chromosome was inferred to have undergone a 3:1 segregation in generation 1, then each MN daughter contained an extra, intact copy of the MN chromosome in addition to the re-incorporated MN chromatid. In this scenario, we compared the transcriptional yield of the MN haplotype in each MN daughter cell to the ranges of both monosomic transcription and disomic transcription to assess the normality or deficiency of transcription of the re-incorporated MN chromatid.

The data of normalized transcription ratios, inferred DNA copy number states and the transcriptional yields of MN chromatids and haplotypes are summarized in Supplementary Table 2.

### Same-cell correlative live-fixed imaging

For the same-cell correlative live-fixed imaging experiments using MDC1-expressing cells (Fig. 3a,b), cells were seeded on 35-mm ibiTreat Grid-500 dishes (Ibidi) with a gridded imaging surface. Live-cell imaging was performed using wide-field fluorescence microscope as described in the ‘Live-cell imaging’ section. At the end of live-cell imaging, cells were immediately fixed by incubation with methanol for 10 min at −20 °C. A snapshot of the last imaging frame including a differential interference contrast image was taken to visualize the grids of the coverslip dish. The grid coordinate information and the last snapshot of the time-lapse images were used to locate the cells of interest after fixation and indirect immunofluorescence imaging.

For experiments using the RPE-1 RFP–NLS GFP–H2B cells (Extended Data Fig. 9a) and the modified U2OS 263 cells (Fig. 4d,e), live-cell imaging was performed as described above. At the end of the live-cell imaging, cells were fixed by incubation with methanol for 10 min at −20 °C for RPE-1 RFP–NLS GFP–H2B cells or 4% paraformaldehyde for 20 min at room temperature (modified U2OS 263 cells). Cells of interest were located according to the grid coordinates for subsequent indirect immunofluorescence analysis.

### Indirect immunofluorescence and confocal microscopy of fixed cells

Cells were fixed and prepared for indirect immunofluorescence and confocal microscopy as previously described^11,26^.

Images were acquired on a Nikon Ti-E inverted microscope (Nikon) with a Yokogawa CSU-22 spinning disk confocal head with the Borealis modification or a Ti2 inverted microscope fitted with a CSU-W1 spinning disk. *Z*-stacks of 0.4–0.7 μm spacing were collected using a CoolSnap HQ2 CCD camera (Photometrics) or a Zyla 4.2 sCMOS camera (Andor) with a ×60/1.40 NA or a ×100/1.45 NA Plan Apochromat oil-immersion objective (Nikon).

The following antibodies were used for indirect immunofluorescence imaging: phospho γH2AX (Ser139) (Millipore, 05-636-I; 1:400); H3K27ac (Active Motif, 39133; 1:200); MDC1 (Abcam, ab11171; 1:1,000); MDC1 (Sigma-Aldrich, M2444; 1:1,000); phospho RNA PolII S5 (Millipore, MABE954, clone 1H4B6; 1:400); Cdk9 (Cell Signaling, 2316; 1:10); CDK12 (Abcam, ab246887; 1:400); 53BP1 (Santa Cruz, 22760S; 1:100); H3K27me3 (ThermoFisher Scientific, MA511198; 1:1,000); H3K9ac (Cell Signaling, 9649S; 1:400); H3K9me2 (Cell Signaling, 9753S; 1:400); POM121 (Proteintech, 15645-1-AP; 1:200); phospho H3T3 (Millipore, 07-424, 1:12,000); phospho H3S10 (Abcam, ab47297; 1:200); and fibrillarin (Abcam, ab4566; 1:500). Staining of Dam-methylated DNA in fixed cells was done using purified GFP-tagged ^m6^A-Tracer protein as previously described^53^.

### Image analysis of fixed-cell experiments

Two image analysis pipelines were used in this study. To characterize the transcriptional state and chromatin alterations in micronuclei (Fig. 1e–g and Extended Data Figs. 4 and 5), we used customized ImageJ/Fiji macros as previously described^26^. To characterize MN bodies or MN-body-like structures (Figs. 3 and 4 and Extended Data Figs. 9, 10 and 12), we used a Python-based analysis pipeline^47^ with additional preprocessing procedures performed using ImageJ/Fiji software. Both pipelines overall consisted of the following steps: (1) cells of interest were identified and their primary nuclei were segmented; (2) micronuclei or re-incorporated MN chromosomes (or chromosome bridges) were identified and segmented; (3) mean FI values for labelled proteins or DNA were quantified over the segmented regions of interest (ROIs).

#### Analysis of the transcription and chromatin alterations in micronuclei

Image analysis of micronuclei in immunofluorescence experiments were performed as previously described^26^.

#### Image segmentation and ROI identification

First, the three-dimension (*xyz*) images of primary nuclei and micronuclei were segmented using the Li or Otsu thresholding method in ImageJ/Fiji with the DNA (Hoechst) signal as input. Second, the nuclear segmentations were further refined using the ImageJ/Fiji functions Watershed and Erode to remove connecting pixels bordering abutting nuclei. Third, nuclear segmentations containing primary nuclei and micronuclei were manually selected as ROIs using the ImageJ/Fiji functions Wand Tool. ROIs from one single focal plane where primary nuclear and micronuclear DNA signal were in focus were manually selected and used for the following quantification.

#### FI quantification

The mean FI of labelled proteins or DNA was quantified over the selected ROIs from their corresponding microscope fluorescence channels. For quantification of nuclear proteins (for example, RNAP2-Ser5ph, RFP–NLS; Fig. 1e–g and Extended Data Fig. 4) or labelled DNA (Hoechst), the mean FI values were calculated for micronuclear ROIs and primary nuclear ROIs, respectively. These mean FI values were subtracted by the mean FI value of the non-nuclear background to obtain the background-subtracted mean FI. The background-subtracted mean FI of micronuclei were divided by the background-subtracted mean FI of the corresponding PN to obtain the MN/PN mean FI ratios. For quantification of histone modifications, including H3K27ac, H3K9ac, H3K9me2, H3K27me3 and γH2AX, the MN/PN mean FI ratios of these marks were further divided by the MN/PN mean FI ratio of DNA (Hoechst) to obtain the DNA-normalized FI ratios. To analyse micronuclei with intact or ruptured NE, micronuclei with MN/PN mean FI ratios of NLS below 0.1 relative to the PN were considered ruptured and above 0.3 were considered intact. Micronuclei with MN/PN FI ratios in between were excluded for analysis, as the assessment of NE integrity is not definitive.

In addition, the background-subtracted mean FI of RNAP2-Ser5ph are shown as exact FI values without normalizing to the background-subtracted mean FI of the corresponding PN (Extended Data Fig. 4b).

#### Analysis of generation 2 re-incorporated MN chromosomes

Analysis of incorporated MN chromosomes were performed primarily using an automated script written in Python^47^. Further details are available upon request.

#### Image segmentation and ROI identification

Step 1, all candidate primary nuclei within the three-dimensional images were identified and segmented either using the Li thresholding method with DNA (Hoechst) signal as the input or the Otsu or Li thresholding method with RNA Pol2S5 signal as the input. The nuclear segmentations were further refined using binary mask operations similar to the procedures described above for the micronuclei analysis pipeline.

Step 2, a smaller cropped three-dimensional image (*z*-stack) was generated for each segmented PN object to minimize variability in the fluorescence signal across the entire image. Only PN objects located within the middle 50% of our images were analysed to minimize the uneven illumination due to the large field of view of the camera (2,048 × 2,048 pixels). From these cropped three-dimension images, a single focal plane in which the MDC1 or ^m6^A-Tracer (hereafter m6T) signal was in focus was selected. This single focal plane was determined as the focal plane with the largest standard deviation (s.d.) in the FI distribution of all pixels (which is used as an estimator of the strongest overall signal) from the MDC1 or m6T channel. The cropped *xy* images and segmentations for each candidate PN objects were then analysed.

Step 3, primary nuclei that contained potential re-incorporated MN chromosomes were located using the presence of large foci of MDC1 or m6T. To identify MDC1 or m6T large foci for each candidate PN, the FI of all pixels within the corresponding nuclear segmentation were quantified to generate a nuclear FI distribution. Positive pixels were selected if their FI > 2 s.d. above the mean for the nuclear FI distribution. These positive pixels were subject to an area size filter (300 pixels) to remove small noise pixels so that only connected-positive pixels larger than the size filter were kept to generate the final ROIs for MDC1 and m6T. For nuclei with multiple valid MDC1 and m6T foci (for example, from two or more MN chromosomes), all foci were analysed together per each nucleus. Additionally, to increase detection accuracy of m6T foci from cells with a variable m6T expression, candidate nuclei of interest were manually screened using ImageJ/Fiji. The *xy* coordinates of these candidate nuclei were supplemented as additional inputs for the analysis pipeline and used for locating valid nuclei containing m6T foci according to the above criteria (FI > 2 s.d. and > 300 pixels) using Python.

Step 4, segmentations for other objects (nuclear or subnuclear structures) that were used for the analysis were generated. Specifically, the ROIs for the primary nuclei were defined by excluding the MDC1 and m6T segmentations as well as the nucleoli segmentations from the original nuclear segmentation (see step 1). The nucleoli segmentations were generated using the lower 10% of the primary nuclear FI distribution of RNAP2-Ser5ph. This 10% (percentile) cutoff was determined by comparing with the nucleoli segmentations using fibrillarin-positive signals (which are pixels for which FI > 3 s.d. above the mean for its total nuclear FI distribution; Extended Data Fig. 9f): the highest overlap with the nucleoli segmentations using the fibrillarin-positive signal was achieved using the lower 10% of the nuclear RNAP2-Ser5ph FI as the cutoff for nucleoli segmentations. ROIs or the γH2AX-positive areas were defined by γH2AX-positive pixels for which FI > 3 s.d. above the mean for the nuclear FI distribution. The ROI area occupancy ratio of the γH2AX-positive pixels within the m6T foci was used to define different levels of γH2AX in re-incorporated m6T micronuclei (Fig. 4c and Extended Data Fig. 10e).

Step 5, a randomized control ROI was segmented by randomly picking a smaller area (at a size similar to the MDC1 or m6T ROI) within the primary nuclear ROI generated above for each cell containing a MDC1 or m6T foci. The random picking process was performed using our Python-based analysis pipeline.

To validate the accuracy of MDC1 and m6T foci identification, the ROI segmentations of a random subset of cells were manually examined. The mis-identification rate of our automated pipeline using random subsets of cells was typically lower than 10%. Additionally, for the m6T dataset after quantification (see below), outliers were also manually examined. The mis-identification rate for the outliers of m6T dataset was 25%. These mis-identified m6T foci (*n* = 27) were mostly m6T-positive micronuclei immediately next to the primary nuclei and were distributed near-symmetrically at the top and bottom of the measurement distribution. These images were excluded during the analysis.

#### FI quantification

The mean FI of labelled proteins or DNA was quantified over the segmented ROIs above from their corresponding microscope channels. All mean FI values were then background subtracted by the corresponding mean FI of the non-nuclear background. The background-subtracted mean FI of MDC1 or m6T ROIs (see step 3 above) and the background-subtracted mean FI of the randomized control ROI (see step 5 above) were normalized to the background-subtracted mean FI of the corresponding primary nuclear ROI (see step 4 above) to obtain the normalized mean FI ratios of labelled proteins or DNA.

For quantification of histone modifications, including H3K27ac, H3K9ac, H3K9me2, H3K27me3, γH2AX, H3S10ph and H3T3ph, the normalized mean FI ratios of these marks were further divided by the normalized mean FI ratio of DNA (Hoechst) to obtain the DNA-normalized FI ratios. This controlled for signal enrichment due to chromosome compaction.

#### Analysis of re-incorporated fragments from chromosome bridge resolution

Quantification of RNAP2-Ser5ph of incorporated bridge segments after bridge resolution and cell division (Extended Data Fig. 12a) was performed in a similar manner to the re-incorporated micronuclei as described above, with ROIs for MN-body-like structures from bridge segments identified using MDC1-positive signal (FI > 2 s.d. and >300 pixels).

#### Analysis of incorporated MN chromosomes for the correlative live-fixed imaging

For quantification of RNAP2-Ser5ph and H3K27ac in incorporated MN chromosomes marked by MDC1 foci (Fig. 3a,b), the analysis was performed in a similar manner as described above except for the following differences: (1) The MN-body segmentations were manually drawn along the MDC1-enriched pixels; and (2) the control (or PN) segmentations were manually defined as a large PN region excluding nucleoli. ROIs for MN bodies and controls were manually selected over these segmentations for a single *Z*-plane where MN bodies were in focus. The mean FI MN body-to-control ratios were obtained by dividing the background-subtracted mean FI of the MN body ROIs to the background-subtracted mean FI of the control ROIs. Note that the time-lapse images were analysed manually to assign the re-incorporated daughters and rupture events, and the low sample size allowed for the manual quantification analysis. In addition, note that some daughter cells with MN bodies that were included in the analysis had new micronuclei in the generation 2 samples, independent of the detected re-incorporated MN chromosome.

Graphical data from the imaging analyses were plotted and statistical analyses were performed using GraphPad Prism (v.9.4.0; GraphPad Software).

### Analysis of live-cell imaging data for MS2-marked nascent transcription

To quantify the MS2-marked transcription level (Figs. 2e,f and 4d and Extended Data Fig. 8), an automated script written in Python was used with assists using ImageJ/Fiji.

#### Image segmentation and LacO and MS2 foci tracking

MN cells with the LacO and MS2 (LacO/MAS) reporter or control cells without MN were manually identified from the image series, and image series of interest were divided into three parts: generation 1 interphase, mitosis, and generation 2 interphase.

For time frames covering the generation 1 interphase (for both control cells and for MN cells), all primary nuclei were segmented using the cellpose package^54^, and all LacO/MS2 foci (in both MN and PN) were segmented using the Yen segmentation method^55^ for each time frame. The primary nuclei and LacI foci of interest in the first time point were identified by finding the object segmentation with the shortest distance to a user-provided *xy* centroid coordinate of the nucleus and the LacO/MS2 focus, respectively. For the following time points, the same nuclei and LacO/MS2 foci were automatically identified by finding the object segmentation for which the distance was the shortest to the identified nuclei and the LacO/MS2 foci segmentations from the previous time point (or time points). The identification of the LacO/MS2 foci and their corresponding primary nuclei was manually evaluated to assist the tracking of the correct LacO/MS2 foci. The *xy* centroid coordinates of the segmentations were used for estimating the object moving distance above.

For time points around mitosis, the nuclei and LacO/MS2 foci were manually tracked in ImageJ/Fiji to accurately identify the partitioning of LacO/MS2 foci into daughter cells during mitotic exit.

For time frames after mitosis (generation 2), daughter primary nuclei were segmented and tracked as described for the first interphase. For LacO/MS2 foci segmentation tracking, we used a combination of several criteria for technical reasons. Because long-term binding of LacI to LacO can impair DNA replication, we terminated LacI gene expression at around 18 h after mitotic shake-off. This led to a loss of LacI signal in a subset of daughter cells during the generation 2 interphase, particularly evident at later time points. For these cells that had lost the LacI signal, we quantified the FI distribution for the nuclear MS2 signal and identified positive MS2 pixels for which FI > 3 s.d. above the mean for the nuclear MS2 FI distribution. The enrichment of such nuclear MS2-positive foci (if present) was then used for the LacO/MS2 foci segmentation tracking for the subsequent time points. For time points in which LacI foci persisted, we tracked the LacO/MS2 foci as described for generation 1 interphase. If no LacO/MS2 foci could be identified during generation 2 interphase, time points were annotated as having no MS2 expression, and therefore no segmentation was performed.

Additionally, we manually examined the nuclei and LacO/MS2 foci tracking because the estimation of object movement using minimal centroid moving distance could lead to incorrect tracking when objects swap positions between time points. For these time points, additional *xy* pixel centroid coordinates for the nuclei and LacO or MS2 foci were obtained using ImageJ/Fiji and supplemented the automated object tracking.

#### Fl quantification for ROIs

ROIs for the LacO or MS2 foci and the corresponding PN were selected from their segmentation as described above. ROIs from one single focal plane where the LacI signal was in focus were used for FI quantification. Based on these ROIs, the mean FI of the MS2 signal for LacO/MS2 foci and matching PN pairs and of the non-nuclear background was quantified. The background-subtracted mean FI of LacO/MS2 foci was divided by the background-subtracted mean FI of the primary nuclear areas (excluding the LacO/MS2 foci) to obtain the normalized MS2 level.

For time points that were annotated as having no MS2 expression in generation 2, a value of 1.7 was assigned because this value is the minimal detectable normalized MS2 signal for the positive MS2 foci in the controls (see details below) for the purpose of plotting the graphs. To obtain this value, we analysed 23 control cells over two cell-cycles for which the LacO/MS2 focus was located within the PN for both generations 1 and 2. We quantified the normalized MS2 level during the generation 2 interphase for time points at which the cells had lost the LacI signal after we stopped LacI expression. These control cells maintained MS2 reporter transcription, and their LacO/MS2 foci were detected and segmented from MS2-positive pixels (FI > 3 s.d. above the mean for the nuclear MS2 FI distribution, as described above). The lowest of the normalized mean FI for all detected MS2-positive foci (*n* = 477) from all imaged time points above was 1.7, defining 1.7 as the minimum detectable mean MS2 signal in the control experiments. Therefore, 1.7 was used as the normalized MS2 signal when no positive MS2 foci could be detected. Note that this is a conservative estimate because the actual MS2 level can be lower as measured for some MN bodies in which the LacI signal was present and can be used for segmentation. In other words, this should underestimate the degree of MS2 signal loss for MN chromosomes that are in a normal generation 2 daughter cells.

### SDS–PAGE and western blotting

Lysis of RPE-1 Dam and control RPE-1 cells (Extended Data Fig. 10c) was performed after trypsinization and washes with PBS by adding an equal volume of a 2× lysis buffer (100 mM Tris-HCl pH 6.8, 4% SDS and 12% β-mercaptoethanol). Whole-cell lysates were denatured at 100 °C for 10 min, Laemmli–SDS sample buffer (Boston BioProducts) was added, and the samples were subjected to SDS–PAGE on NuPAGE 4–12% Bis-Tris gradient gels (Novex Life Technologies). The proteins were then transferred onto a nitrocellulose membrane (Millipore). The membranes were blocked using Odyssey blocking buffer (LI-COR) and were incubated with primary antibodies for 1 h at room temperature or overnight at 4 °C. The primary antibodies and dilutions used were anti-mCherry rabbit 1:1,000 (ab167453, Abcam) and anti-GAPDH mouse 1:5,000 (ab9485, Abcam). After washes with PBS-T, we incubated the membranes with the fluorescent secondary antibodies IRDye 680RD donkey anti-rabbit 1:5,000 (926-68073, LI-COR) and IRDye 800CW donkey anti-mouse 1:5,000 (926-32212, LI-COR) for 1 h at room temperature. Membranes were visualized using a ChemiDoc MP imaging system (Bio-Rad). Note that the images shown in Extended Data Fig. 10c were cropped to show the bands at the protein size. The full scan (uncropped) blots are shown in Supplementary Fig. 1.

### FACS

RPE-1 megaDam cells (see the section ‘Cell culture and cell line construction’) were analysed by FACS for mCherry expression using a LSR Fortessa flow cytometer (BD) (Extended Data Fig. 10b). Cells were stained with DAPI for dead-cell exclusion and live cells were analysed for their percentage of mCherry-positive cells (excluding autofluorescent cells by gating PE relative to FITC). Data were recorded using FACSDiva (v.8.0; BD) software, and FlowJo (v.10.7.1; BD) was used for data analysis. Examples of the gating strategy are shown in Supplementary Fig. 2.

### Genomic analysis of bridge clones

#### DNA sequencing

Genomic DNA was purified using a DNeasy Blood and Tissue kit (Qiagen) and was then fragmented on a Covaris M220 instrument according to the manufacturer’s protocol. Libraries were prepared using Swift S2 Acel reagents on a Beckman Coulter Biomek i7 liquid handling platform from approximately 200 ng of DNA with 14 cycles of PCR amplification. DNA libraries were quantified on a Qubit 2.0 Fluorometer (Life Technologies) and fragment size distributions were evaluated on a Agilent TapeStation 2200 (Agilent Technologies). Pooled libraries were further evaluated with low-pass sequencing on an Illumina MiSeq and then sequenced to approximately 5× mean genome coverage on an NovaSeq 6000 instrument (Illumina) with 2× 150 bp paired-end configuration in the Molecular Biology Core Facilities at Dana-Farber Cancer Institute. Haplotype-specific DNA copy number was calculated using the same workflow as previously described^11,48^. DNA rearrangements shown in Extended Data Fig. 11 were taken from previous analyses^11^.

#### RNA-seq

RNA extraction, library preparation and sequencing were conducted at Azenta Life Sciences. In brief, total RNA was extracted from fresh-frozen cell pellet samples using a RNeasy Plus Universal mini kit (Qiagen). RNA samples were quantified using a Qubit 2.0 Fluorometer (Life Technologies), and RNA integrity was evaluated using a TapeStation 4200 (Agilent Technologies). An ERCC RNA Spike-In Mix kit (4456740, ThermoFisher Scientific) was added (but not used) and sequencing libraries were prepared using a NEBNext Ultra RNA Library Prep kit for Illumina (NEB). The quality of the sequencing libraries were validated on a Agilent TapeStation (Agilent Technologies), and the concentration of the libraries were quantified using a Qubit Fluorometer and by quantitative PCR (KAPA Biosystems). The samples were sequenced on an Illumina instrument (4000 or equivalent) with 2× 150 bp paired-end configuration with an average of around 60 million reads per sample.

Bulk RNA-seq data were aligned using STAR (v.2.7.10a) with the same parameters as single-cell RNA-seq data processing. As the estimated fraction of duplicate reads in bulk RNA-seq data was above 5%, we followed all steps of post-alignment processing (including duplicate removal) as described in the best practice of GATK. All post-alignment processing was carried out using GATK (v.4.2.6.1). The remaining steps of RNA-seq data processing were identical to the processing of scRNA-seq data.

We first generated feature counts from analysis-ready RNA-seq bam files using featureCounts from Subread 2.0.1 (https://subread.sourceforge.net) and then calculated total TPM^47^. We performed a similar global TPM normalization step for each sample by scaling the TPM values by a constant factor to match the median expression of genes that are transcribed bi-allelically and have mean TPM between 1 and 1,000 (6,683 total). After global normalization, we calculated allelic transcription of each gene using the same procedure as for the single-cell transcriptome analysis. To assess the transcriptional yield of each gene copy, we further divided both the total and allelic transcriptional levels by the DNA copy number in 250 kb local intervals; the DNA copy number was determined from whole-genome DNA sequencing data generated on the same culture. To quantify transcriptional changes relative to normal transcription, we normalized the transcription yield (both total and allelic) in bridge and control clones by the transcriptional level in the parental RPE-1 sample. The final TPM ratio (gene level) was then used to assess transcriptional changes (both total and haplotype-specific) independent of copy-number variation.

#### ATAC-seq

The preparation of nuclei, transposition and amplification by PCR were performed as previously described^56^. In brief, cells were trypsinized and washed twice with PBS. Then, 10,000 cells in 5 μl PBS were transposed in 42.5 μl of transposition buffer (33 mM Tris acetate buffer, 66 mM potassium acetate, 10 mM magnesium acetate, 0.1% NP-40, 16% DMF, 0.004× protease inhibitor cocktail and ddH_2_O to 42.5 µl) and 2.5 μl of TDE1 Illumina Tn5 transposase. The transposition reaction was conducted for 30 min at 37 °C, followed immediately by DNA purification using a ZYMO DNA Clean and Concentrator 5 kit (Zymo Research). Cycle-determining quantitative PCR was conducted to amplify libraries and stop amplification before saturation. The amplified libraries were purified using a ZYMO DNA Clean and Concentrator 5 kit and quantified by using a Qubit 2.0 Fluorometer. The libraries were normalized and pooled based on quantitative PCR analysis and were subsequently sequenced on a NovaSeq S1 instrument (Illumina) with 2× 50 bp paired-end configuration or a NextSeq instrument (Illumina) with 2× 38 bp configuration at the Bauer Core Facility of Harvard University.

Reads were trimmed to remove adapter sequences and then aligned to hg38 using Bowtie2 (ref. ^57^) with the following parameters: -X2000–rg-id. Chromatin accessibility peak calling was conducted as previously described^58^. In brief, we first performed peak-calling on each sample using MACS2 (ref. ^59^) with the following parameters/options: –nomodel,–nolambda,–keep-dup all,–call-summits.

We then combined and merged overlapping peaks (within 400 bp) called from all samples to create a unique list of peaks (259,036 total). The fragment count within each peak was calculated using the getCounts function from chromvar^60^, and then normalized using the preprocessCore normalize.quantiles function^61^.

To assess changes in chromatin accessibility in the bridge clones, we first divided the quantile-normalized fragment count^60^ for every peak by the local DNA copy number (250 kb bins) to account for DNA gain or loss, which was almost exclusively restricted to chromosome 4.

To account for technical variation during library preparation, we applied a permutation approach to generate a reference ATAC profile for each individual clone based on the ATAC profiles in control clones. First, for each ATAC-seq peak, we generated a replicate set of 50 peaks with similar GC content and average accessibility in the control samples (ten control RPE-1 subclones) using the getBackgroundPeaks(<normalized.counts>, bias = <gc.bias>) command from chromvar^60^.

Here <gc.bias> was calculated for each peak region (300 bp) and <normalized.counts> denotes the ATAC fragment counts in the ten control subclone samples. Assuming the replicate peaks are subject to similar technical variation, we then used the ATAC-seq densities of replicate peaks as the null distribution for the peak of interest to perform intra-sample background normalization by random permutations.

During each permutation, we randomly selected 1 out of 50 replicate peaks for each peak in a given genomic interval to create a random reference ATAC profile. By generating a sufficient number of reference ATAC profiles through permutations, we could assess the statistical deviation of the observed ATAC profile in each genomic interval from the null distribution generated by permutations. To enable a sufficient number of permutations, we only considered intervals with at least 10 peaks per Mb (with a maximum of 50^10^ ≈ 9.8 × 10^16^ permutations). We performed around 10^6^ permutations for each interval (lower than the number of all possible permutations) to identify outliers with *P* values on the order of 10^−6^.

The above-described permutation sampling was performed on the fragment counts in each sample in 1, 5 or 10 Mb intervals. Based on the null distributions derived from random permutations, we then calculated the fold change of the observed ATAC density relative to the mean of the null distribution. The re-centered fold change of ATAC signal is shown in Extended Data Fig. 12b. We further estimated the likelihood of the observed average ATAC density of each interval in each sample based on the null distributions generated by permutations (an example is shown in Extended Data Fig. 12d). Shown in Figs. 5b and 12c are the average fold change of ATAC signals across all 12 bridge clones.

Our permutation sampling directly accounts for GC bias. It also accounts for non-uniform peak density across the genome. Additionally, in our analysis, we primarily focused on clonal or near-clonal changes that are more likely generated by the initial formation and resolution of bridges than subclonal changes that are more likely to have arisen downstream. We therefore focused on intervals with a significant reduction in the average ATAC fold change (<0.70).

### Reporting summary

Further information on research design is available in the Nature Portfolio Reporting Summary linked to this article.

## Online content

Any methods, additional references, Nature Portfolio reporting summaries, source data, extended data, supplementary information, acknowledgements, peer review information; details of author contributions and competing interests; and statements of data and code availability are available at 10.1038/s41586-023-06157-7.

## Supplementary information

Supplementary Information This file contains Supplementary Figs. 1–2 and the Supplementary Methods, it also contains descriptions for Supplementary Tables 1–2 and Videos 1–6 (files supplied separately).

Reporting Summary

Supplementary Table 1

Supplementary Table 2

Supplementary Video 1

Supplementary Video 2

Supplementary Video 3

Supplementary Video 4

Supplementary Video 5

Supplementary Video 6

## Data Availability

The authors declare that the data supporting the findings of this study are available within the paper and its supplementary information files. Sequencing data are available from the Sequencing Read Archive under BioProject identifiers PRJNA602546 and PRJNA867730. The raw data and all other datasets generated in this study are available from the corresponding authors upon reasonable request. Source data are provided with this paper.

## Source data

Source Data Fig. 1
Source Data Fig. 2
Source Data Fig. 3
Source Data Fig. 4
Source Data Extended Data Fig. 4
Source Data Extended Data Fig. 5
Source Data Extended Data Fig. 8
Source Data Extended Data Fig. 9
Source Data Extended Data Fig. 10
Source Data Extended Data Fig. 12

## Code availability

Scripts and pipelines used for all sequencing data analysis and for image analysis are available at the GitHub online repository (https://github.com/chengzhongzhangDFCI/nature2023)^47^.

## References

[1] Baylin, S. B. & Jones, P. A. Epigenetic determinants of cancer. *Cold Spring Harb. Perspect. Biol*. 10.1101/cshperspect.a019505 (2016). 10.1101/cshperspect.a019505 PMC5008069 27194046

[2] Flavahan, W. A., Gaskell, E. & Bernstein, B. E. Epigenetic plasticity and the hallmarks of cancer. *Science*10.1126/science.aal2380 (2017). 10.1126/science.aal2380 PMC5940341 28729483

[3] Feinberg AP, Koldobskiy MA, Gondor A (2016). Epigenetic modulators, modifiers and mediators in cancer aetiology and progression. Nat. Rev. Genet..

[4] Zink D, Fischer AH, Nickerson JA (2004). Nuclear structure in cancer cells. Nat. Rev. Cancer.

[5] Maciejowski J, Hatch EM (2020). Nuclear membrane rupture and its consequences. Annu. Rev. Cell Dev. Biol..

[6] de las Heras, J. I. & Schirmer, E. C. in *Cancer Biology and the Nuclear Envelope: Recent Advances May Elucidate Past Paradoxes* (eds Schirmer, E. C. & de las Heras, J. I.) 5–26 (Springer, 2014).

[7] Patey DH, Scarff RW (1928). The position of histology in the prognosis of carcinoma of the breast. Lancet.

[8] Zhang CZ (2015). Chromothripsis from DNA damage in micronuclei. Nature.

[9] Maciejowski J, Li Y, Bosco N, Campbell PJ, de Lange T (2015). Chromothripsis and kataegis induced by telomere crisis. Cell.

[10] Ly P (2019). Chromosome segregation errors generate a diverse spectrum of simple and complex genomic rearrangements. Nat. Genet..

[11] Umbreit, N. T. et al. Mechanisms generating cancer genome complexity from a single cell division error. *Science*10.1126/science.aba0712 (2020). 10.1126/science.aba0712 PMC7347108 32299917

[12] Stephens PJ (2011). Massive genomic rearrangement acquired in a single catastrophic event during cancer development. Cell.

[13] Cortes-Ciriano I (2020). Comprehensive analysis of chromothripsis in 2,658 human cancers using whole-genome sequencing. Nat. Genet..

[14] Mammel, A. E. & Hatch, E. M. Genome instability from nuclear catastrophe and DNA damage. *Semin. Cell Dev. Biol.*10.1016/j.semcdb.2021.03.021 (2021). 10.1016/j.semcdb.2021.03.021 PMC8494860 33839019

[15] Hatch EM, Fischer AH, Deerinck TJ, Hetzer MW (2013). Catastrophic nuclear envelope collapse in cancer cell micronuclei. Cell.

[16] Karoutas A (2019). The NSL complex maintains nuclear architecture stability via lamin A/C acetylation. Nat. Cell Biol..

[17] Hoffelder DR (2004). Resolution of anaphase bridges in cancer cells. Chromosoma.

[18] Crasta K (2012). DNA breaks and chromosome pulverization from errors in mitosis. Nature.

[19] Mohr L (2021). ER-directed TREX1 limits cGAS activation at micronuclei. Mol. Cell.

[20] Tang S, Stokasimov E, Cui Y, Pellman D (2022). Breakage of cytoplasmic chromosomes by pathological DNA base excision repair. Nature.

[21] Ly P (2017). Selective Y centromere inactivation triggers chromosome shattering in micronuclei and repair by non-homologous end joining. Nat. Cell Biol..

[22] Picelli S (2013). Smart-seq2 for sensitive full-length transcriptome profiling in single cells. Nat. Methods.

[23] Mackenzie KJ (2017). cGAS surveillance of micronuclei links genome instability to innate immunity. Nature.

[24] Santaguida S, Amon A (2015). Short- and long-term effects of chromosome mis-segregation and aneuploidy. Nat. Rev. Mol. Cell Biol..

[25] Leibowitz ML (2021). Chromothripsis as an on-target consequence of CRISPR–Cas9 genome editing. Nat. Genet..

[26] Liu S (2018). Nuclear envelope assembly defects link mitotic errors to chromothripsis. Nature.

[27] Liu S, Pellman D (2020). The coordination of nuclear envelope assembly and chromosome segregation in metazoans. Nucleus.

[28] Pelham-Webb B (2021). H3K27ac bookmarking promotes rapid post-mitotic activation of the pluripotent stem cell program without impacting 3D chromatin reorganization. Mol. Cell.

[29] Hsiung CC (2016). A hyperactive transcriptional state marks genome reactivation at the mitosis-G1 transition. Genes Dev..

[30] Kang H (2020). Dynamic regulation of histone modifications and long-range chromosomal interactions during postmitotic transcriptional reactivation. Genes Dev..

[31] Janicki SM (2004). From silencing to gene expression: real-time analysis in single cells. Cell.

[32] Klaasen SJ (2022). Nuclear chromosome locations dictate segregation error frequencies. Nature.

[33] Shanbhag NM, Rafalska-Metcalf IU, Balane-Bolivar C, Janicki SM, Greenberg RA (2010). ATM-dependent chromatin changes silence transcription in cis to DNA double-strand breaks. Cell.

[34] Pankotai T, Bonhomme C, Chen D, Soutoglou E (2012). DNAPKcs-dependent arrest of RNA polymerase II transcription in the presence of DNA breaks. Nat. Struct. Mol. Biol..

[35] Kim J, Sturgill D, Tran AD, Sinclair DA, Oberdoerffer P (2016). Controlled DNA double-strand break induction in mice reveals post-damage transcriptome stability. Nucleic Acids Res..

[36] Haber, J. *Genome Stability: DNA Repair and Recombination* (Taylor and Francis, 2013).

[37] Greil F, Moorman C, van Steensel B (2006). DamID: mapping of in vivo protein–genome interactions using tethered DNA adenine methyltransferase. Methods Enzymol..

[38] Smith JJ, Timoshevskiy VA, Saraceno C (2021). Programmed DNA elimination in vertebrates. Annu. Rev. Anim. Biosci..

[39] Sarkies P, Reams C, Simpson LJ, Sale JE (2010). Epigenetic instability due to defective replication of structured DNA. Mol. Cell.

[40] Lukas C (2011). 53BP1 nuclear bodies form around DNA lesions generated by mitotic transmission of chromosomes under replication stress. Nat. Cell Biol..

[41] Harrigan JA (2011). Replication stress induces 53BP1-containing OPT domains in G1 cells. J. Cell Biol..

[42] Spies J (2019). 53BP1 nuclear bodies enforce replication timing at under-replicated DNA to limit heritable DNA damage. Nat. Cell Biol..

[43] van Steensel B, Smogorzewska A, de Lange T (1998). TRF2 protects human telomeres from end-to-end fusions. Cell.

[44] Lemmens B (2018). DNA replication determines timing of mitosis by restricting CDK1 and PLK1 activation. Mol. Cell.

[45] Santaguida S (2017). Chromosome mis-segregation generates cell-cycle-arrested cells with complex karyotypes that are eliminated by the immune system. Dev. Cell.

[46] Papathanasiou, S. and Zhang, H. Systems and methods for capturing cells. Provisional patent PCT/US 2019/023696 (2019).

[47] Papathanasiou, S., Mynhier, N., Liu, S., Brunette, G. & Li, L. Source codes and selected intermediate and final data, https://github.com/chengzhongzhangDFCI/nature2023.git10.5281/zenodo.7792974 (2023).

[48] Tourdot RW, Brunette GJ, Pinto RA, Zhang CZ (2021). Determination of complete chromosomal haplotypes by bulk DNA sequencing. Genome Biol..

[49] Becskei A, Kaufmann BB, van Oudenaarden A (2005). Contributions of low molecule number and chromosomal positioning to stochastic gene expression. Nat. Genet..

[50] Reinius B, Sandberg R (2015). Random monoallelic expression of autosomal genes: stochastic transcription and allele-level regulation. Nat. Rev. Genet..

[51] Brennecke P (2013). Accounting for technical noise in single-cell RNA-seq experiments. Nat. Methods.

[52] Stegle O, Teichmann SA, Marioni JC (2015). Computational and analytical challenges in single-cell transcriptomics. Nat. Rev. Genet..

[53] van Schaik T, Vos M, Peric-Hupkes D, Hn Celie P, van Steensel B (2020). Cell cycle dynamics of lamina-associated DNA. EMBO Rep..

[54] Stringer C, Wang T, Michaelos M, Pachitariu M (2021). Cellpose: a generalist algorithm for cellular segmentation. Nat. Methods.

[55] Yen JC, Chang FJ, Chang S (1995). A new criterion for automatic multilevel thresholding. IEEE Trans. Image Process..

[56] Buenrostro JD, Wu B, Chang HY, Greenleaf WJ (2015). ATAC-seq: a method for assaying chromatin accessibility genome-wide. Curr. Protoc. Mol. Biol..

[57] Langmead B, Salzberg SL (2012). Fast gapped-read alignment with Bowtie 2. Nat. Methods.

[58] Kartha, V. K. et al. Functional inference of gene regulation using single-cell multi-omics. *Cell Genom*. 10.1016/j.xgen.2022.100166 (2022). 10.1016/j.xgen.2022.100166 PMC9534481 36204155

[59] Zhang Y (2008). Model-based analysis of ChIP-seq (MACS). Genome Biol..

[60] Schep AN, Wu B, Buenrostro JD, Greenleaf WJ (2017). chromVAR: inferring transcription-factor-associated accessibility from single-cell epigenomic data. Nat. Methods.

[61] Bolstad, B. preprocessCore: a collection of pre-processing functions. R package version 1.62.1 10.18129/B9.bioc.preprocessCore (2023).

[62] Rao, P. N. & Johnson, R. T. *Premature Chromosome Condensation* (Academic Press, 1982).

[63] Xu J (2017). Landscape of monoallelic DNA accessibility in mouse embryonic stem cells and neural progenitor cells. Nat. Genet..

