# Supplementary material for: Heritable transcriptional defects from aberrations of nuclear architecture
Source: Nature. 2023 Jun 7;619(7968):184–92. doi: 10.1038/s41586-023-06157-7 (PMC10322708; doi:10.1038/s41586-023-06157-7)
Supplement: Supplementary file 1 — This file contains Supplementary Figs. 1–2 and the Supplementary Methods, it also contains descriptions for Supplementary Tables 1–2 and Videos 1–6 (files supplied separately). [file 41586_2023_6157_MOESM1_ESM.pdf]

nature portfolio

https://doi.org/10.1038/s41586-023-06157-7

Supplementary information

Heritable transcriptional defects from aberrations of nuclear architecture

In the format provided by the authors and unedited

Heritable transcriptional defects from aberrations of nuclear architecture

Stamatis Papathanasiou[1,2,3#], Nikos A. Mynhier[1,2*], Shiwei Liu[2,4*], Gregory Brunette[1,2], Ema Stokasimov[1,2], Etai Jacob[5,6,7], Lanting Li[5,6,8], Caroline Comenho[9,10,11], Bas van Steensel[12], Jason D. Buenrostro[9,10,11], Cheng-Zhong Zhang[5,6,8#], David Pellman[1,2,5,9,13#]

Supplementary Information

Table of Contents:

1. Cover Sheet____1
2. Supplementary Figure 1 legend ____________________________2
3. Supplementary Figure 2 legend ____________________________2
4. Supplementary Table 1 legend_____________________________2-3
5. Supplementary Table 2 legend_____________________________3-6
6. Supplementary Videos 1-6 legends__________________________6-7
7. Supplementary Figure 1__________________________________8
8. Supplementary Figure 2__________________________________9
9. Method on the quantification of transcriptional variation_________10-11

Supplementary Figure 1. Gel source image.

Raw images of Western Blot gels of Extended Data Fig. 10c. Left, antibody for mCherry; right, antibody for GAPDH. GAPDH was used as loading control at the same gel as mCherry. Yellow boxes show the cropped regions presented in Extended Data Fig. 10c.

Supplementary Figure 2. FACS gating strategy.

Examples of the gating strategy for the FACS analysis experiment of the Extended Data Fig. 10b. RPE-1 megaDam cells were analyzed for mCherry expression (PE channel) and were stained with DAPI for dead cell exclusion. Live cells were then analyzed for their percentage of mCherry positive cells (PE-positive). Auto-fluorescent cells were excluded by gating the PE against the FITC channel. An example of a control sample (without Dox) is shown in (a) and an example of an experimental condition (20 h Dox, 2 h IAA/ASV) is shown in (b). Arrows denote the gating strategy.

Supplementary Table 1. Summary of single-cell RNA-Seq data.

Cells for which RNA-Seq data have been generated are grouped by the experimental design (Column A) and the identity of the ancestor cell (Column B: "Family ID"). Each family consists of cells descended from a single ancestor as identified by live cell imaging and each family member is assigned a unique ID (Column C: "Cell ID"). Columns D-L summarize various quality metrics of each single-cell library that are generated by STAR. Control RPE-1 cells (untreated, FACS, Look-Seq, and Look-Seq2) are included if they have > 6,000 genes with five or more reads; MN related cells (Look-Seq and Look-Seq2, generation 1 and generation 2) are included if they have > 4,000 genes with five or more reads. Column M displays the sequencing

platform on which the RNA-Seq data were generated. Column N reports which cells were used in our control cell panel throughout the computational analysis.

Supplementary Table 2. Summary of single-cell analysis of chromosomes with significant deviation from normal transcription determined using control RPE-1 cells.

Tab 1: Summary of MN chromosome transcription assessed from single-cell data of MN cell/MN sister pairs (generation 1). Column A and B display the family ID and the status of MN nuclear envelope integrity; Column C and D display the identity and parental haplotype of one or multiple chromosomes inferred to have been trapped in the micronucleus; Column E: The normalized transcription yield of the effected homolog of the MN chromosome; Column F: The total DNA copy number of the MN homolog in the MN cell; Column G and H: statistical assessment of the deviation of the observed transcription yield from the expected transcription yield of one normally transcribing chromosome (Column G) or two normally transcribing chromosomes (Column H). The MN chromosome (including its haplotype) and its DNA copy number are determined by a comparison of the observed pattern of transcriptional imbalance between the MN cell and the MN daughter cell and the predicted outcomes based on the segregation pattern of MN chromosomes. Details of this inference is provided in Tab 3. Column I shows the transcriptional status of the MN chromosome. In Family F84 and F206, we identified no chromosome with transcriptional imbalance; we concluded that the MN chromosome underwent 2:2 segregation and was normally transcribed in the MN cell. This is noted as an additional note in Column J. The transcription data of MN chromosomes (both homologs) in each family are shown in Extended Data Fig. 3a.

Tab 2: Summary of MN chromosome transcription assessed from single-cell data of MN daughters/MN nieces (generation 2). Column A and B are similar to Tab 1; Column C shows the number of MN nieces (NA when unavailable). Column D and E display the identity and haplotype of each chromosome inferred to have been partitioned in the micronucleus presented in the mother cell of MN daughters; Column F summarizes the DNA copy-number pattern of the MN chromosome in all family members. A vertical line separates MN daughters from nieces. For the inference of MN chromosome based on transcriptional imbalance in each family, see Tab 4. Columns G-K display the transcriptional yield of each reincorporated MN chromosome and the assessment of their transcriptional status (similar to Columns E-I in Tab 1). Additional notes for each family are again included, as Column L. The transcription data of MN chromosomes (both homologs) in each family are shown in Extended Data Fig. 6.

Tab 3: List of all chromosomes with non-reference transcription status in generation 1 families. Each family (Family ID, Column B) consists of a MN cell (Column D) and a MN sister cell (Column J); both the MN cell identity and the status of NE integrity of the micronucleus (Column A) are determined by live-cell imaging. Each row contains a chromosome with non-reference transcription (Column C; see Extended Data Fig. 2d) in either MN cell or MN sister cell. Columns E-I show the normalized total transcription yield (Column E), haplotype-specific transcription yield assessed by allelic TPM ratios (Column F and H), and the discrete transcriptional copy-number state of both haplotypes (Column G and I) inferred based on the haplotype-specific transcription yield in comparison to reference haplotype-specific transcription derived from control RPE-1 cells (Extended Data Fig. 1d). Columns K-O display the same data types as in Columns E-I for the MN sister cell. By comparing the DNA copy-number ratio between the MN cell and the MN sister for both haplotypes (Column P and Q) against the

segregation patterns associated with micronucleation (Extended Data Fig. 2a), we determine whether the chromosome was in the micronucleus (Column R) and the parental haplotype (Column S) of the MN chromosome. The DNA copy-number pattern of MN chromosomes are recapitulated in Column T. Additional notes are put in Column U and differences from the prior analysis are presented in Column V. When we inferred that only a chromosome arm displays transcriptional imbalance that is consistent with MN segregation, we recalculated the haplotype-specific transcription yield of the effected arm and the statistical assessment of the deviation of the observed transcription yield from the expectation based on the underlying DNA copy number (shown in Tab 1).

Our new allelic transcription analysis revealed more than three pre-existing chromosome/arm-level copy-number changes in two families, F73 and F79. We therefore excluded these two families from the final summary.

Tab 4: List of all chromosomes with non-reference transcription status in generation 2 families. Columns A-C are the same as in Tab 2; Columns D-I, J-O, P-U, V-AA each summarize the transcription yield of a potentially abnormally transcribing chromosome in MN daughters (first two groups) and in MN nieces (second two groups, when applicable). Columns AB and AC summarize the discrete transcriptional copy-number state of both haplotypes in all the cells, from which we determine whether the pattern of transcriptional imbalance indicates a relationship to micronucleation (Column AD) and the affected haplotype (Column AE). Columns AF recapitulates the DNA copy number of the MN chromosome in all family members. In Family 262, one MN niece cell (niece 1) displayed significant transcriptional changes in multiple chromosomes that are not shared by the other niece cell (niece 2) or reciprocal to transcriptional changes in the MN daughters; we excluded this niece cell in the inference of MN chromosome.

In both Tab 3 and Tab 4, chromosomes with non-reference transcription but inferred to be not related to micronucleation are shaded in gray. These include (1) pre-existing duplications that are shared by all family members; (2) mis-segregation events between family members (resulting in 0:2 copy number imbalance); and (3) other transcriptional variation patterns that do not match the predicted outcomes shown in Extended Data Fig. 2a.

Supplementary Videos 1 and 2. Examples of Look-Seq2 experiments.

The videos start during generation 1. An MN cell (GFP-H2B channel) and its sister are followed. The micronucleus ruptures (loss of RFP-NLS) late during the generation 1 interphase. Both cells divide, generating MN daughters and MN cell nieces in generation 2. The chromosome from the micronucleus is reincorporated into one of the daughters.

Supplementary Video 3. Live imaging of nascent transcripts after two chromosomes from micronuclei are reincorporated into daughter nuclei.

The reporter locus is visualized with LacI-SNAP; nascent transcription is visualized with MCP-Halo; and the loss of the general nuclear pool of MCP-Halo indicates rupture of the NE of both micronuclei. One reporter-marked chromosome recovers transcription whereas the other does not.

Supplementary Video 4. Formation of an MN-body marked by SNAP-MDC1 – example 1.

In generation 1, a micronucleus forms and undergoes NE rupture (loss of RFP-NLS). The damaged chromosome from the micronucleus is only decorated with SNAP-MDC1 during

mitosis. In generation 2, the SNAP-MDC1 marked chromosome is reincorporated into a daughter nucleus, forming a long-lived MN-body.

Supplementary Video 5. Formation of an MN-body marked by SNAP-MDC1 – example 2.

Similar to the Supplementary Video 4. Note that the MN has already undergone rupture at the start of the video.

Supplementary Video 6. Formation of an MN-body marked by SNAP-MDC1 – example 3.

Similar to the Supplementary Video 4. Note that the MN has already undergone rupture at the start of the video and that in this example only one of the two reincorporated micronuclei appear to form an MN-body.

Supplementary Figure 1

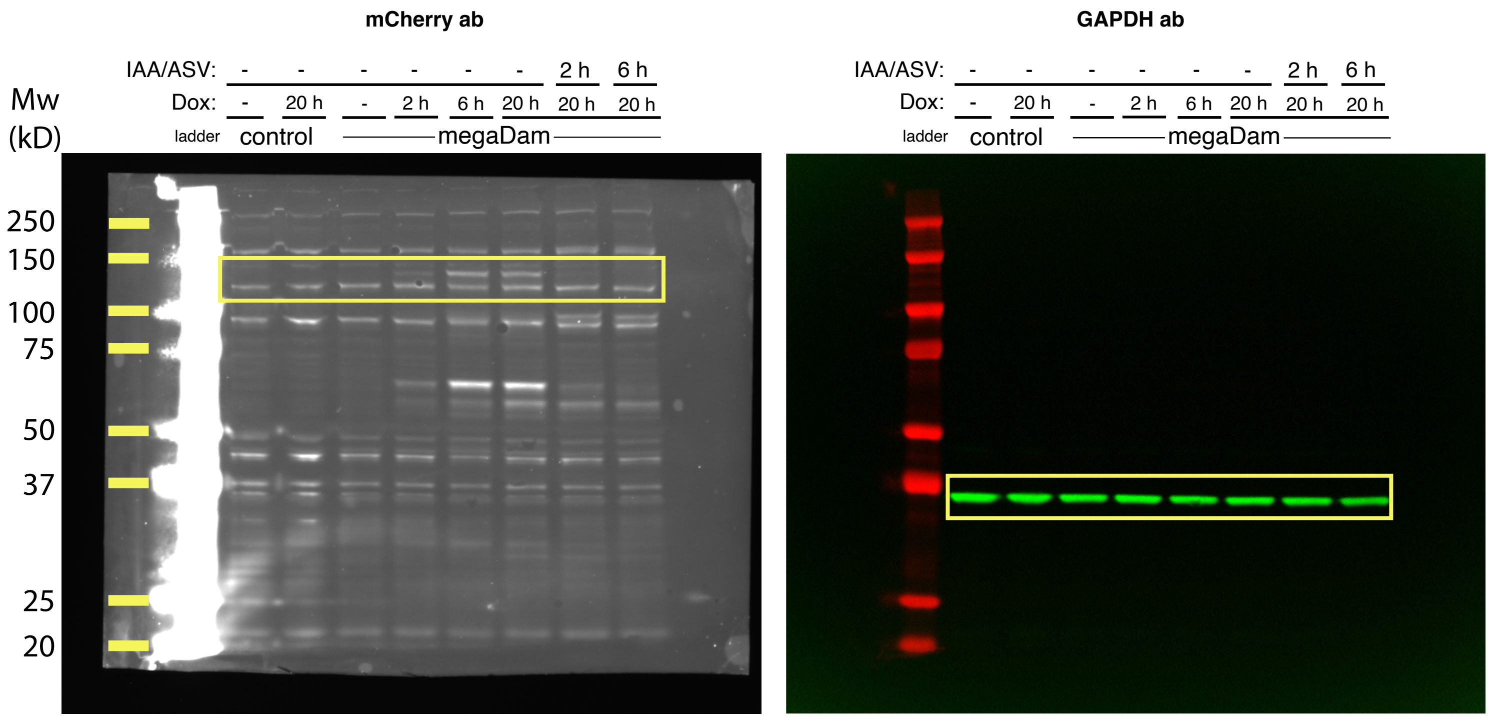

Supplementary Figure 2

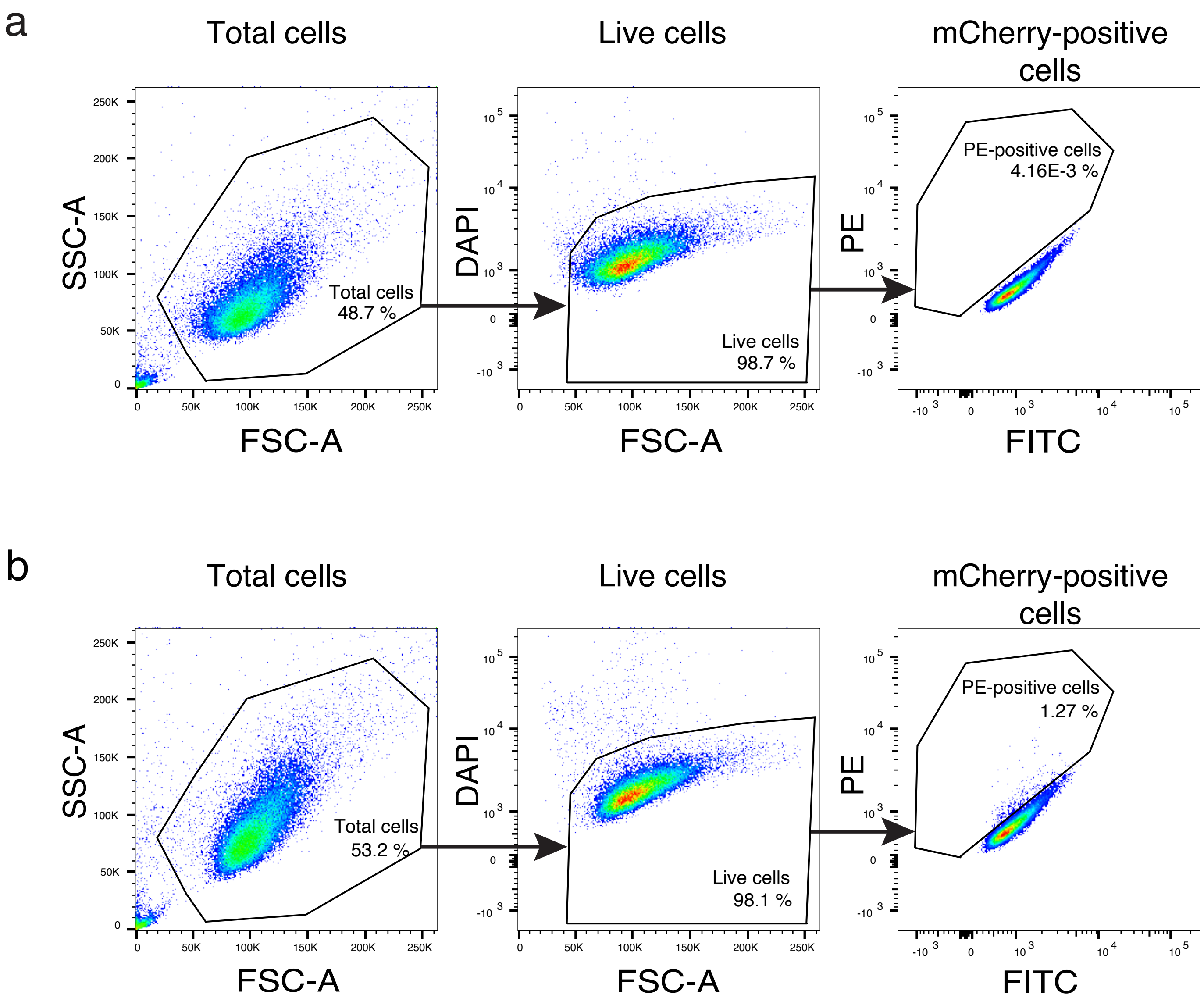

Method on the quantification of transcriptional variation

Basic definitions

$\mathrm{TPM}_i^{(\mathrm{a})}$	TPM (transcripts per million) of gene i in cell (a)
$\langle \mathrm{TPM}_i \rangle$	mean TPM of gene i in control cells
$T_i^{(\mathrm{a})} = \mathrm{TPM}_i^{(\mathrm{a})} / \langle \mathrm{TPM}_i \rangle$	TPM ratio of gene i in cell (a)
α_i	(cell-independent) weight factor for gene i in the average TPM ratio
$\mathrm{Var}(T_i)$	variance of TPM ratio in control cells
$g^{(\mathrm{a})}$	global normalization factor of TPM for cell (a)
$t_i^{(\mathrm{a})} = g^{(\mathrm{a})} T_i^{(\mathrm{a})}$	TPM ratio after global normalization
$\mathrm{AF}_i^{(\mathrm{a})}$	allelic fraction of transcripts of gene i in cell (a)
$\mathrm{Var}(\mathrm{AF}_i)$	variance of allelic fraction of gene i in control cells
β_i	weight factor for gene i in the average allelic fraction

Quantification of transcriptional changes relative to normal disomic transcription

For a list of genes with expression in a single cell (a) given by $\mathrm{TPM}_i^{(\mathrm{a})}$ and reference expression (mean expression in control cells) given by $\langle \mathrm{TPM}_i \rangle$, we consider a weighted average TPM ratio of

$$\sum_i \alpha_i \cdot \frac{\mathrm{TPM}_i^{(\mathrm{a})}}{\langle \mathrm{TPM}_i \rangle} = \sum_i \alpha_i T_i^{(\mathrm{a})}, \quad \sum_i \alpha_i = 1, \tag{1}$$

where $T_i^{(\mathrm{a})}$ is the normalized expression of gene i in cell (a), α_i is the weight for gene i. If we assume $T_i^{(\mathrm{a})}$ of different genes to be independent, the variance of this weighted TPM ratio is given by

$$\mathrm{Var}\left(\sum_i \alpha_i T_i^{(\mathrm{a})}\right) = \sum_i \alpha_i^2 \cdot \mathrm{Var}\left(T_i^{(a)}\right) = \sum_i \alpha_i^2 \cdot \mathrm{Var}(T_i). \tag{2}$$

Here $\mathrm{Var}(T_i)$ is the variance of TPM ratio for gene i that is estimated from control cells. It is straightforward to verify that the variance in Eq. (2) is minimized when

$$\alpha_i \propto \frac{1}{\mathrm{Var}(T_i)}. \tag{3}$$

We weighed individual TPM ratios $T_i^{(\mathrm{a})}$ by the inverse of its variance estimated from control RPE-1 cells (198 total). To ensure numerical stability of the weighted average, we capped the weights α_i at the 95% of all weights across the genome (excluding those from Chr.X).

We used a similar strategy to calculate the average allelic fraction over multiple genes (both in 10Mb intervals and across each chromosome) as

$$\sum_i \beta_i \cdot \mathrm{AF}_i^{(\mathrm{a})}, \quad \beta_i \propto \frac{1}{\mathrm{Var}(\mathrm{AF}_i)} \text{ and } \sum_i \beta_i = 1 \tag{4}$$

where $\mathrm{AF}_i^{(\mathrm{a})}$ denotes the allelic fraction of gene i in cell (a) and β_i is the weight. We also capped the weights β_i at the 95% of all weights across the genome (excluding those from Chr.X). For Chr.X transcripts with a predominant Xa (active X) bias, we calculated the allelic fraction as a simple average.

Cell-specific TPM normalization

As TPM represents relative transcript abundance, the TPM values in a single cell are uniformly amplified or attenuated by up- or down-regulation of one or a few highly transcribed genes that significantly alter the total mRNA content. To account for such global changes, we performed an additional global normalization of the TPM ratios in each cell:

$$t_i^{(a)} = g^{(a)} \cdot \frac{\mathrm{TPM}_i^{(a)}}{\langle \mathrm{TPM}_i \rangle} = g^{(a)} T_i^{(a)}. \tag{5}$$

The global scaling factor $g^{(a)}$ was introduced to normalize the inverse variance-weighted mean TPM ratio to unity in each cell (a), i.e.,

$$g^{(a)} \sum_i \alpha_i T_i^{(a)} = 1. \tag{6}$$

Therefore,

$$g^{(a)} = \left(\sum_i \alpha_i T_i^{(a)} \right)^{-1}, \quad \alpha_i = \mathrm{Var}(T_i)^{-1} \Big/ \sum_j \mathrm{Var}(T_j)^{-1} \tag{7}$$

We used the scaled TPM ratios t_i for the average TPM ratio calculation. For most control cells, the scaling factor $g^{(a)}$ is close to 1; we therefore did not re-calibrate the mean $\langle \mathrm{TPM}_i \rangle$ and variance $\mathrm{Var}(T_i)$ of TPM values calculated from control cells.
