# Supplementary material for: Heritable transcriptional defects from aberrations of nuclear architecture
Source: Nature. 2023 Jun 7;619(7968):184–92. doi: 10.1038/s41586-023-06157-7 (PMC10322708; doi:10.1038/s41586-023-06157-7)
Supplement: Supplementary file 2 — Reporting Summary [file 41586_2023_6157_MOESM2_ESM.pdf]

nature portfolio

Corresponding author(s): David Pellman

Last updated by author(s): Apr 3, 2023

Reporting Summary

Nature Portfolio wishes to improve the reproducibility of the work that we publish. This form provides structure for consistency and transparency in reporting. For further information on Nature Portfolio policies, see our Editorial Policies and the Editorial Policy Checklist.

Statistics

For all statistical analyses, confirm that the following items are present in the figure legend, table legend, main text, or Methods section.

n/a	Confirmed	
☐	☒	The exact sample size (n) for each experimental group/condition, given as a discrete number and unit of measurement
☒	☐	A statement on whether measurements were taken from distinct samples or whether the same sample was measured repeatedly
☐	☒	The statistical test(s) used AND whether they are one- or two-sided<br>*Only common tests should be described solely by name; describe more complex techniques in the Methods section.*
☒	☐	A description of all covariates tested
☐	☒	A description of any assumptions or corrections, such as tests of normality and adjustment for multiple comparisons
☐	☒	A full description of the statistical parameters including central tendency (e.g. means) or other basic estimates (e.g. regression coefficient) AND variation (e.g. standard deviation) or associated estimates of uncertainty (e.g. confidence intervals)
☐	☒	For null hypothesis testing, the test statistic (e.g. F, t, r) with confidence intervals, effect sizes, degrees of freedom and P value noted<br>*Give P values as exact values whenever suitable.*
☒	☐	For Bayesian analysis, information on the choice of priors and Markov chain Monte Carlo settings
☒	☐	For hierarchical and complex designs, identification of the appropriate level for tests and full reporting of outcomes
☐	☒	Estimates of effect sizes (e.g. Cohen's d, Pearson's r), indicating how they were calculated

Our web collection on statistics for biologists contains articles on many of the points above.

Software and code

Policy information about availability of computer code

Data collection	Fixed and live microscope images were captured with Metamorph 7.10.2.240 (Molecular Devices) or NIS Elements 4.30 AR or newer versions (Nikon Instruments).<br>Western images were visualized using a ChemiDoc MP Imaging System (BioRad). FACS data were recorded using the FACSDiva 8.0 (BD) software.
Data analysis	Fixed and live microscope images were analyzed using ImageJ/FIJI using custom macros and a Python-based custom imaging analysis pipeline. A detailed description of the FIJI/ImageJ macros and the Python-based analysis pipeline are described in the Methods section. Scripts and pipelines used for all sequencing data analysis and for image analysis are available at github on-line repository (https://github.com/chengzhongzhangDFCI/nature2023 and https://github.com/stambio/MNbody_scripts). Sequencing data and image analyses are described in detail in the Methods section. Graphical data from the imaging analysis were plotted and statistical analysis was performed using Graphpad Prism 9.4.0 (Graphpad Software). FlowJo 10.7.1 (BD) was used for the FACS data analysis.

For manuscripts utilizing custom algorithms or software that are central to the research but not yet described in published literature, software must be made available to editors and reviewers. We strongly encourage code deposition in a community repository (e.g. GitHub). See the Nature Portfolio guidelines for submitting code & software for further information.

Data

Policy information about availability of data

All manuscripts must include a data availability statement. This statement should provide the following information, where applicable:

- Accession codes, unique identifiers, or web links for publicly available datasets
- A description of any restrictions on data availability
- For clinical datasets or third party data, please ensure that the statement adheres to our policy

The data supporting the findings of this study are available within the paper and its Supplemental Information files. Sequencing data are available from the Sequencing Read Archive (SRA) under BioProjects PRJNA602546 and PRJNA867730. The raw data and all other data sets generated in this study are available from the corresponding authors upon reasonable request.

Field-specific reporting

Please select the one below that is the best fit for your research. If you are not sure, read the appropriate sections before making your selection.

☒ Life sciences ☐ Behavioural & social sciences ☐ Ecological, evolutionary & environmental sciences

For a reference copy of the document with all sections, see nature.com/documents/nr-reporting-summary-flat.pdf

Life sciences study design

All studies must disclose on these points even when the disclosure is negative.

Sample size	We did not compute statistical analyses to predetermine sample sizes prior to performing each individual experiment. Our sample sizes were chosen according the standards of our lab and based on similar studies (eg. Zhang et al., 2015 Nature; Liu et al. 2018, Nature).
Data exclusions	For image analysis of MDC1-labeled MN-bodies (Fig 3 and Extended Data Fig. 8), only cells within the middle 50% of the field of views were analyzed to minimize the uneven illumination due to the large size of the camera, as described in the Methods section.<br>For image analysis of m6T-labeled MN-bodies, after automated image analysis, we manually examined the outliers to estimate the detection accuracy of our automated image analysis pipeline. Among these outliers, we identified ~25% of cells with incorrectly segmented MN-bodies, which were micronuclei adjacent to the primary nuclei that are difficult to separate in our analysis pipeline. We excluded these images containing the incorrect segmentation in our final analysis. One or a few data points were excluded from the plots for presentation purposes, but all data points were included in the analysis (one data point in Fig.1e, Fig.3c, ExtFig.5a, ExtFig.5c, ExtFig.5f, ExtFig6d, ExtFig.10e, ExtFig.10g; three data points in ExtFig.10f, Fig.3d; six data points in ExtFig.5i; eleven data points in Fig.1e). Details on the exclusions applied in the scRNAseq analysis are provided in the Methods.
Replication	All experiments had biological replicates and the majority of experiments were replicated at least two times, where the replicates were technically successful. The details of the replicate numbers are provided for each experiment. When the number of replicates are reported, these are all biological replicates.
Randomization	For the imaging analysis experiments, the cells quantified in each experiment were randomly sampled from the total population of cells on the coverslips. Other random allocation was not relevant because cells from different conditions were assumed to be similar except for the conditions that were tested. The allocation into experimental groups was done by the user based on the experimental conditions of each sample analyzed. All different experimental conditions were performed in separate wells (imaging coverslips).
Blinding	Investigators were not blinded for the experimental groups for the bulk sequencing and the live- and fixed- imaging experiments, but the analysis or data acquisition were performed in an unbiased manner (users acquired data without knowing the results). The majority of the images were analyzed in an automated unbiased manner using our custom image analysis pipelines. For the single-cell RNA sequencing experiments (Look-Seq2) the analysis was blinded.

Reporting for specific materials, systems and methods

We require information from authors about some types of materials, experimental systems and methods used in many studies. Here, indicate whether each material, system or method listed is relevant to your study. If you are not sure if a list item applies to your research, read the appropriate section before selecting a response.

Materials & experimental systems

n/a	Involved in the study
☐	☒ Antibodies
☐	☒ Eukaryotic cell lines
☒	☐ Palaeontology and archaeology
☒	☐ Animals and other organisms
☒	☐ Human research participants
☒	☐ Clinical data
☒	☐ Dual use research of concern

Methods

n/a	Involved in the study
☒	☐ ChIP-seq
☐	☒ Flow cytometry
☒	☐ MRI-based neuroimaging

Antibodies

Antibodies used

The following antibodies were used for indirect immunofluorescence in this study: phospho gammaH2AX (Ser139) (Millipore #05-636-I, 1:400), H3K27ac (Active Motif #39133, 1:200), MDC1 (Abcam #ab11171, 1:1000), MDC1 (Sigma-Aldrich # M2444, 1:1000), phospho RNA PolII S5 (Millipore #MABE954, clone 1H4B6, 1:400), Cdk9 (Cell Signaling #2316, 1:10), Cdk12 (Abcam #ab246887, 1:400), 53BP1 (Santacruz #22760S, 1:100), H3K27me3 (Thermo Fisher #MA511198, 1:1000), H3K9ac (Cell Signaling #9649S, 1:400), H3K9me2 (Cell Signaling #9753S, 1:400), POM121 (Proteintech 15645-1-AP, 1:200), phospho H3T3 (Millipore #07-424, 1:12000), phospho H3S10 (Abcam #ab47297, 1:200) and Fibrillarin (Abcam # ab4566, 1:500). For western blots, the following primary antibodies were used: The primary antibodies and dilutions used were anti-mCherry rabbit 1:1000 (ab167453, Abcam) and anti-GAPDH mouse 1:5000 (ab9485, Abcam). The fluorescent secondary antibodies are IRDye 680RD Donkey anti-rabbit 1:5000 (926-68073, LICOR Biosciences) and IRDye 800CW Donkey anti-mouse 1:5000 (926-32212, LICOR Biosciences).

Validation

gammaH2AX (Ser139) (Millipore #05-636-I) antibody was previously used and validated by irradiation in Crasta et al., Nature 2012.
RNA PolII S5 (Millipore #MABE954) was used in Lin et al., EMBO J 2018
H3K27ac (Active Motif #39133) was used in Alekseyenko et al., Genes & Development 2015,
MDC1 (Abcam #ab11171) and MDC1 (Sigma-Aldrich # M2444) were used in Lukas et al., Nature 2011
53BP1 (Santacruz #22760S) was used in Passerini et al., Nature Communications 2016,
H3K9ac (Cell Signaling #9649S) was used in Weinert et al., Cell 2018,
H3K27me3 (Thermo Fisher #MA511198) was used in Beuzelin et al., Front Physiol 2020,
Fibrillarin (Abcam # ab4566) was used in Wang et al., Cell 2018,
mCherry (Abcam #ab167453-100ul) was used in Lattao et al., Dev Cell 2021,
Cdk9 (Cell Signaling #2316) was recommended by the R. Young lab (MIT) and was used in Verma et al., Mol Cell Biol 2019,
Cdk12 (Abcam #ab24688) was recommended by the R. Young lab (MIT) and was used in Liu et al., Cancer Gene Ther 2022,
phospho H3T3 (Millipore #07-727, 1:12000) was validated by signal enrichment on mitotic chromosomes and was used in Hadders et al., J Cell Biol 2020,
phospho H3S10 (Abcam #ab47297, 1:200) was validated by signal enrichment on mitotic chromosomes and was used in Pelham-Webb et al., Mol Cell 2021.

Eukaryotic cell lines

Policy information about cell lines

Cell line source(s)

U2OS and hTERT RPE-1 were purchased from ATCC or obtained from other laboratories as described in the Methods section. The 2-6-3 U2OS cell line was a gift from the David Spector lab. The RPE-1 TRF2-DN cells were obtained from T. de Lange lab.

Authentication

For RPE-1 cells, authentication was provided by the RNA and DNA sequencing analysis, as well as by their characteristic morphology. For U2OS and U2OS-derived cell lines authentication was performed based on their characteristic morphology.

Mycoplasma contamination

All cell lines used were regularly checked for mycoplasma contamination and no contamination was found. All cells used for experiments were stained with DAPI and examined under X60 or X100 1.4 NA objective lens and no contamination was found.

Commonly misidentified lines
(See ICLAC register)

No commonly misidentified cell lines were used in this study.

Flow Cytometry

Plots

Confirm that:

☒ The axis labels state the marker and fluorochrome used (e.g. CD4-FITC).

☒ The axis scales are clearly visible. Include numbers along axes only for bottom left plot of group (a 'group' is an analysis of identical markers).

☒ All plots are contour plots with outliers or pseudocolor plots.

☒ A numerical value for number of cells or percentage (with statistics) is provided.

Methodology

Sample preparation	RPE-1 cells were trypsinized, washed with PBS and resuspended in 2% FBS containing PBS with for FACS analysis.
Instrument	LSR Fortessa Flow Cytometer (BD)
Software	FACSDiva 8.0 Software (BD) for the recording of the data and FlowJo 10.7.1 (BD) for the analysis were used.
Cell population abundance	50,000 cells were recorded by FACS. Cells were >80% viable and percentages of mCherry positive cells are indicated in figures.
Gating strategy	Cells were gated for FSC height versus area to exclude doublets. Dead cells were excluded using DAPI staining. DAPI negative live cells were analyzed for their percentage of mCherry positive cells.

☒ Tick this box to confirm that a figure exemplifying the gating strategy is provided in the Supplementary Information.
